# Direct use of 1,3-dienes for the allylation of ketones *via* catalytic hydroindation†

**DOI:** 10.1039/d0ra00853b

**Published:** 2020-02-06

**Authors:** Itaru Suzuki, Kensuke Yagi, Shinji Miyamoto, Ikuya Shibata

**Affiliations:** Research Center for Environmental Preservation, Osaka University 2-4 Yamadaoka Suita Osaka 565-0871 Japan shibata@epc.osaka-u.ac.jp; Division of Applied Chemistry, Graduate School of Engineering, Osaka University 2-1, Yamadaoka Suita Osaka 565-0871 Japan

## Abstract

In this study, *in situ* catalytically generated allylic indium from 1,3 dienes and InCl_2_H was developed for use in the allylation of ketones. This protocol resulted in the unprecedented establishment of a successive combining of quaternary C–C bonds, which could then be applied to many types of ketones. Other branched 1,3 dienes and vinyl cyclopropanes, could also be coupled with ketones in a reaction where CuH would not be applicable.

## Abstract 2

In this study, *in situ* catalytically generated allylic indium from 1,3 dienes and InCl_2_H was developed for use in the allylation of ketones.

## Graphical abstract

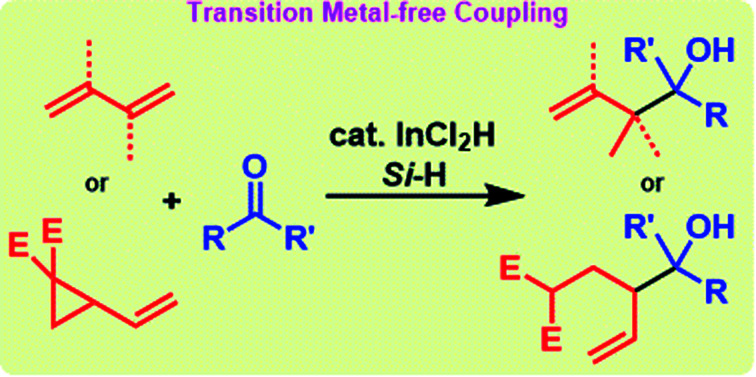

Homoallylic alcohols are useful building blocks in the synthesis of bioactive natural compounds and pharmaceuticals. For these syntheses, the preparation of tertiary compounds has remained challenging regardless of whether or not they are given asymmetrically.^1^ The allylation of ketones with allylic reagents is a typical method for the preparation of these compounds (Scheme 1a).^2^ This method, however, cannot avoid wasteful steps such as the transmetalation between Grignard reagents and B, Si or Sn sources and the reductive generation between allylic halides and low-valent metals. Although this method can be applied to highly stereocontrolled reactions, the wasteful steps are cumbersome and more practical reaction methods are required.

**Scheme 1:**
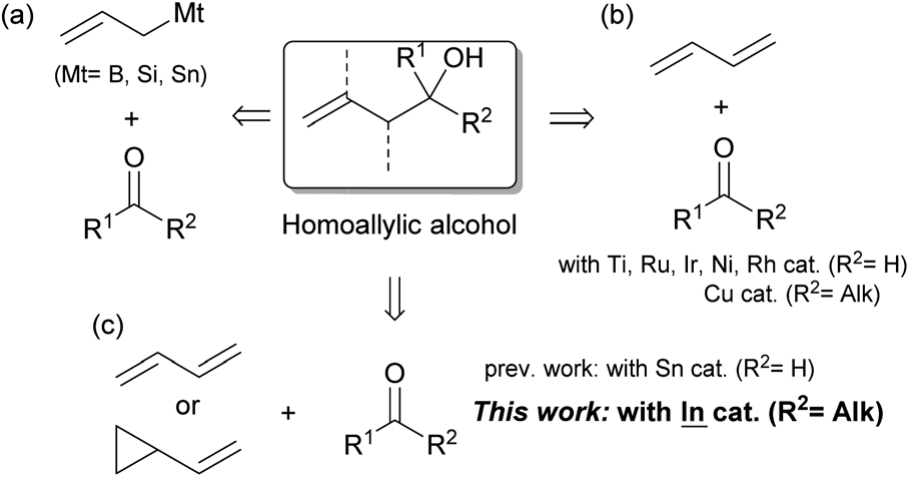
Synthesis of homoallylic alcohols from allylation reagents with ketones.

For this process, 1,3-dienes are an important industrial feedstock that is produced on a massive scale *via* either the cracking of ethylenes or the transformation of biomass. Easily available dienes have recently replaced the conventional allylation of aldehydes with the aid of transition metal catalysts (Scheme 1b).^3^ After the first application using a Ti catalyst by Gendre and Moïse,^4^ Krische has expanded the field with the introduction of Ru-catalyzed stereocontrolled reactions.^5^ Other transition metals such as Ni,^6^ Ir,^7^ and Rh^8^ have contributed to improvements in coupling. A recent adoption of ketones as viable substrates was achieved by Liu and Buchwald *via* proficient Cu–H chemistry.^9^ The scope of possible substrates could be expanded even further,^10^ however, particularly with the use of 1,3-dienes and ketones that possess a variety of functional groups.

Our group has explored the hydrostannylation^11^ or indation^12^ of unsaturated bonds in the preparation of reactive organostannanes or indiums that could be applied to further transformations, although stoichiometric amounts of Sn or In sources must be added to the reaction systems. Recently, a transition metal-free reductive coupling of 1,3-dienes,^13^ or their derivatives such as vinyl cyclopropanes,^14^ with aldehydes catalyzed by Bu_2_SnXH has been developed, but the method would not allow the use of ketones due to the low reactivity of the reaction intermediate, allylic stannanes (Scheme 1c). On the other hand, our group has already developed a process for the hydroindation of 1,3-dienes with a stoichiometric amount of InX_2_H to give allylic indiums followed by the allylation of ketones.^12*b*,12*d*^ Herein, we report the catalytic coupling of 1,3-dienes or vinyl cyclopropanes with ketones through the generation of allylic indiums *via* the hydroindation of 1,3-dienes with a catalytic amount of InX_2_H (Scheme 1c).

We initiated the optimization of the reaction conditions by combining 1,3-butadiene (1a) and acetophenone (2a) in a sealed test tube (Table 1). The gaseous diene 1a was liquefied and weighed before addition to the reaction. Based on our previous reports, InCl_2_OMe generated from InCl_3_/NaOMe and a silane were chosen as indium and hydride sources, respectively.^13^ The desired product 3aa was obtained in a 92% yield as a mixture of the diastereomers (entry 1). The yield was lowered either when no MeOH was used or when the reaction time was cut by half (entries 2 and 3). A screening of the silanes showed that MePhSiH_2_ was the optimal hydride source (entries 4–7). We found that the reaction was finished in 3 h when the reaction temperature was raised to 60 °C (entry 8). Replacing the solvent with MeCN, Et_2_O or toluene did not improve the reaction yield (entries 9–11). It was necessary to add NaOMe to the reaction system for a facile generation of InCl_2_H (entry 12).^16^ It was important to add InCl_3_ to the reaction (entry 13). A radical scavenger, TEMPO, suppressed the progress of the reaction, which implied that this reaction contains a radical process (entry 14). The reaction yield was decreased when the lower amount of the catalyst was employed (entry 15).

**Table 1:** Optimization of the reaction conditions^a^

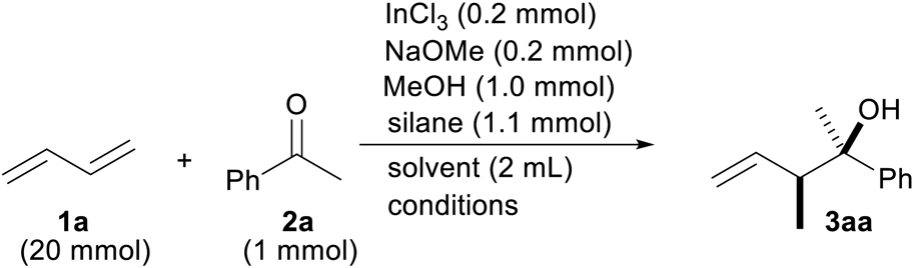				
Entry	Silane	Solvent	Conditions	Yield (%)3aa (*syn* : *anti*)^b^
1	MePhSiH_2_	THF	25 °C, 48 h	92 (80 : 20)
2			25 °C, 48 h w/o MeOH	42 (88 : 12)
3			25 °C, 24 h w/o MeOH	54 (76 : 24)
4	Et_3_SiH	THF	25 °C, 24 h	12^c^
5	Ph_3_SiH	THF	25 °C, 24 h	0
6	Ph_2_SiH_2_	THF	25 °C, 24 h	32 (83 : 17)
7	PhSiH_3_	THF	25 °C, 24 h	54 (83 : 17)
8	MePhSiH_2_	THF	60 °C, 3 h	92 (80 : 20)
9		MeCN		38 (80 : 20)
10		Et_2_O		71 (76 : 42)
11		Toluene		9^c^
12		THF	w/o NaOMe	Trace
13			w/o InCl_3_	0
14			With TEMPO (0.2 mmol)	0
15			With InCl_2_OMe (0.1 mmol)	51 (78 : 22)

a The yields were determined by ^1^H NMR.

b Stereochemistry, see: ref. 15.

c dr could not be determined because of complex of the reaction mixture.

With the optimal reaction conditions in hand (Table 1, entry 8), the scope of the ketones was investigated (Table 2). Electron-deficient substitution on the phenyl ring had a small effect on the reaction yield, but an electron-rich substitution decreased it appreciably (entries 2–6). The efficiency was also attenuated by steric hindrance around the C=O moiety of propiophenone (2g) and butyrophenone (2h) (entries 7 and 8). α-Cyano and -bromo acetophenone 2i and 2j, respectively, reacted sufficiently (entries 9 and 10). The tolerance to reduction of the C–Br bond by InX_2_H under reductive conditions is a characteristic of coupling (entries 3 and 10).^17^ On the other hand, α-methoxy one was unsatisfactory as a reactant probably due to chelation between the OMe and C=O groups with the catalyst that would have promoted a reduction in the ketone 2k (entry 11). β-Keto ester 2l was a good partner even though a similar chelation involving two C=O groups could have happened (entry 12). Both acyclic and cyclic aliphatic ketones were allylated (entries 13–14). Other aromatic rings such as naphthalenes were introduced into the products 3ao and 3ap (entries 15 and 16).

**Table 2:** Scope of ketones^a^

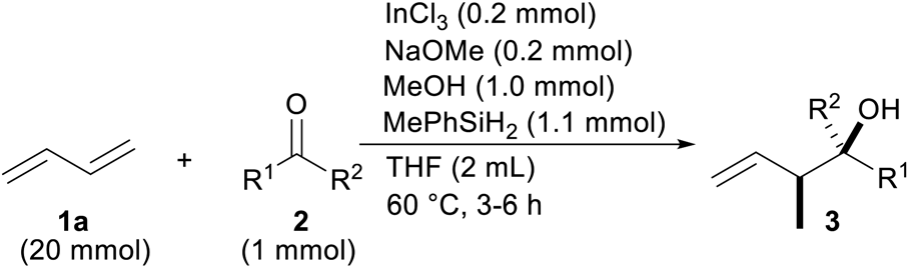					
Entry	R^1^	R^2^	Ketone	Product	Yield (%) (dr)
1	C_6_H_5_	Me	2a	3aa	92 (80 : 20)
2	*p*-ClC_6_H_4_		2b	3ab	83 (79 : 21)
3	*p*-BrC_6_H_4_		2c	3ac	88 (80 : 20)
4	*p*-CNC_6_H_4_		2d	3ad	79 (79 : 21)
5	*p*-MeC_6_H_4_		2e	3ae	53 (75 : 25)
6	*p*-OMeC_6_H_4_		2f	3af	58 (75 : 25)
7	C_6_H_5_	Et	2g	3ag	69 (84 : 16)
8		^*n*^Pr	2h	3ah	66 (84 : 16)
9		CH_2_CN	2i	3ai	75 (82 : 18)
10		CH_2_Br	2j	3aj	74 (84 : 16)
11		CH_2_OMe	2k	3ak	22 (80 : 20)
12		CH_2_CO_2_Et	2l	3al	59 (80 : 20)
13^b^	PhCH_2_CH_2_–(CH_2_)_5_–	Me	2m	3am	73 (57 : 43)
14			2n	3an	75
15	1-Np	Me	2o	3ao	77 (75 : 25)
16	2-Np	Me	2p	3ap	80 (76 : 24)

a The yields were determined by ^1^H NMR.

b Reaction time was 24 h.

Reductive coupling was then applied to other dienes (Scheme 2). In the case of isoprene (1b), two different products, 3ba and 3ba′, were formed even though the reaction was very slow (eqn (1)). The regioselectivity derived from the different structures of the allylic indiums. To our delight, diene 1c made it possible to establish contiguous quaternary C–C bonds with ketones 2a–2c (eqn (2)). To date, construction of contiguous quaternary C–C bonds with a catalyst remains a challenging task in organic synthesis,^18^ and the task has never been realized by the same type of reductive coupling that is catalyzed by transition metal catalysts.

**Scheme 2:**
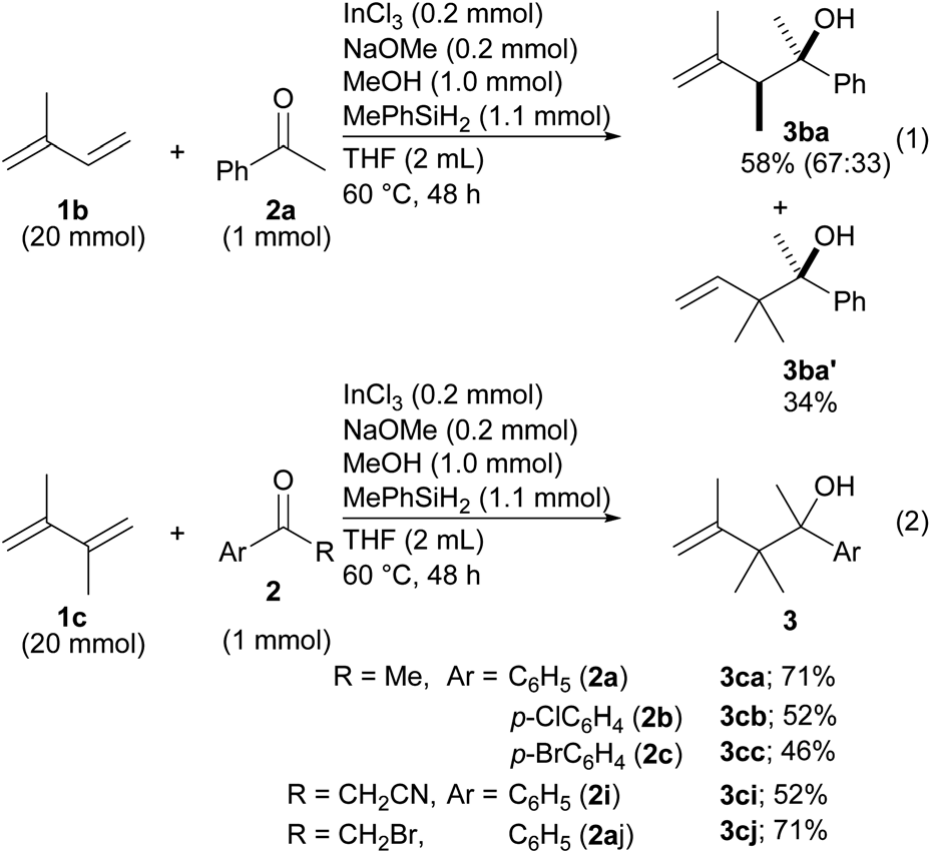
Application of substituted 1,3-butadienes to the coupling.

Our proposal of the reaction mechanism is described in Scheme 3. Initially, prepared InCl_2_(OMe) is reduced by MePhSiH_2_ to give HInCl_2_. The indium radical is formed in the presence of tiny amounts of O_2_ and adds to diene 1a.^17^ The stable allylic radical A extracts hydrogen from InCl_2_H to afford allylic indium B, which regenerates the indium radical. Following the allylation of ketone 2, the generated indium alkoxide 3′ is protonated by CH_3_OH to give the product 3 and InCl_2_(OMe). The reaction mechanism was investigated using a deuterated silane, Ph_2_SiD_2_ (Scheme 4). We found that the product 3aa-CH_2_D was afforded without any other deuterated compound. The product 3aa-CH_2_D is formed from δ-deuterated intermediate B-*d*_1_ as shown in Scheme 4. Further investigation on the reaction mechanism is ongoing in our laboratory.

**Scheme 3:**
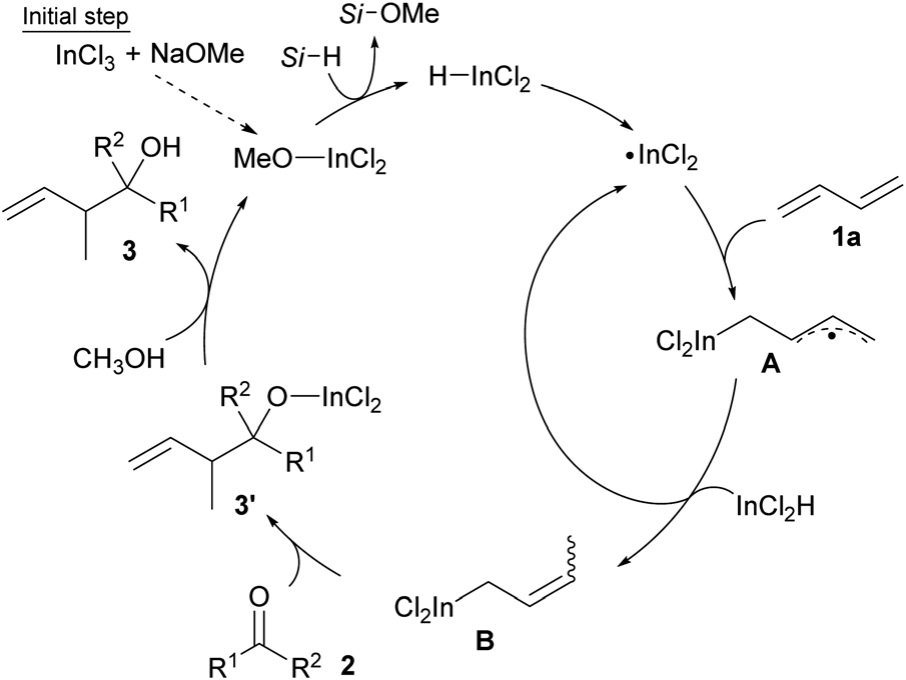
A plausible catalytic cycle.

**Scheme 4:**
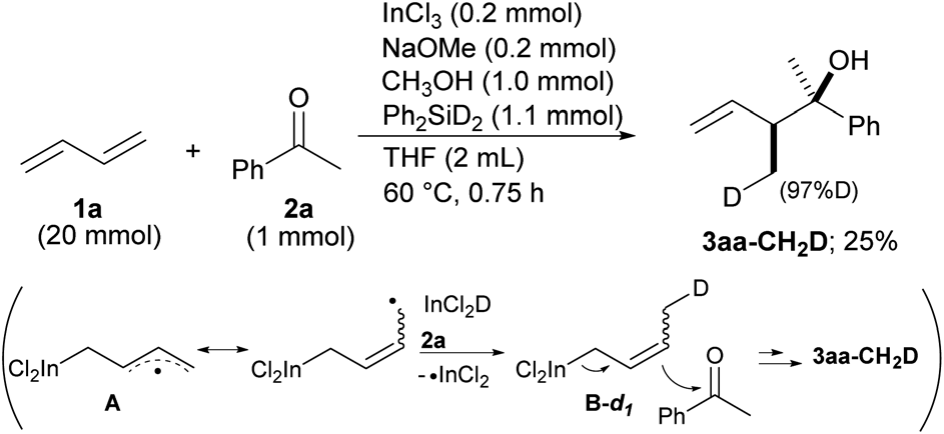
Deuterated experiments of allylation of ketone 2a.

Finally, vinyl cyclopropane 4, a diene derivative, was tested for reductive coupling with ketones.^19^ The desired product 5a was produced in the presence of a radical initiator, V-70L, even though diastereoselectivity was not achieved (Scheme 5, eqn (1)). This could have been caused by low *E*/*Z* selectivity of the allylic indiums. The seminal work developed by Buchwald that was related to a Cu–H catalyzed reaction did not allow the use of vinyl cyclopropane as a reactant because their method has no process for a radical opening of the cyclopropane ring (Scheme 5, eqn (2)).^9^ Our method expands the scope of the dienes by allowing use of their derivatives.

**Scheme 5:**
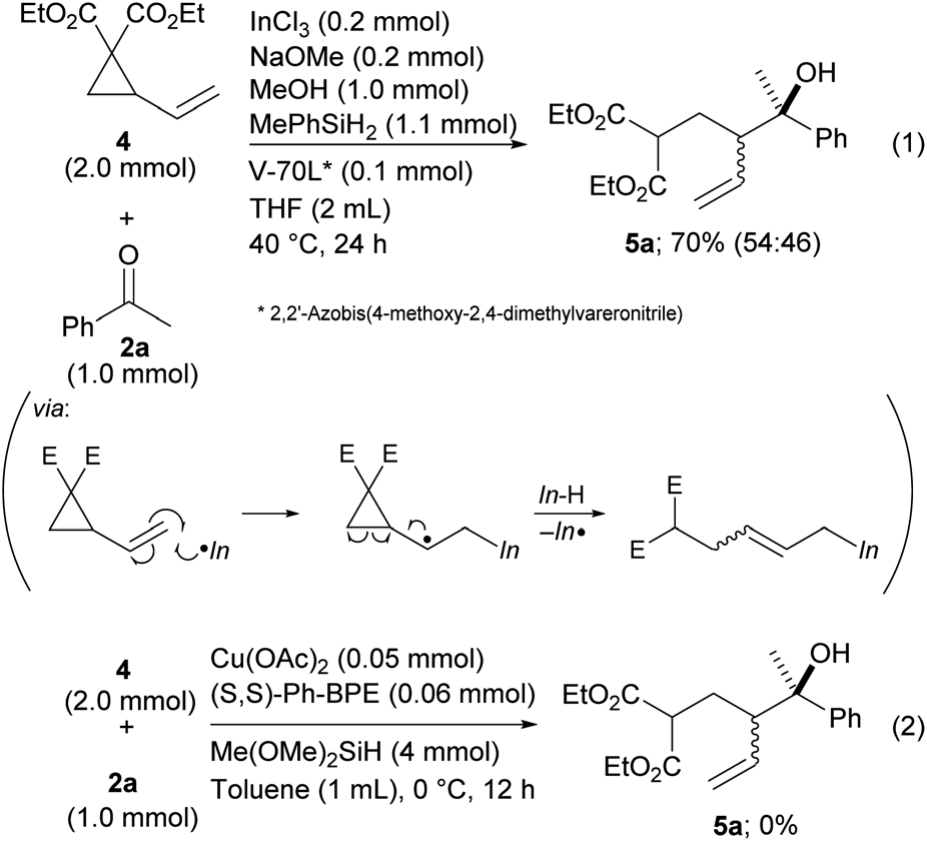
Coupling of vinyl cyclopropanes with ketones.

In summary, we developed a process whereby the reductive coupling of 1,3-dienes with various ketones could be sufficiently catalyzed by HInCl_2_. This approach allowed the introduction of functional groups into homoallylic alcohols, which generated sequential C_*tert*_–C_*tert*_ bonds with expansion to vinyl cyclopropane 4. Application to an asymmetric version of the coupling and improvement of the diastereoselectivity are underway.

## Conflicts of interest

There are no conflicts to declare.

## Supplementary Material

RA-010-D0RA00853B-s001
