# Supplementary material for: Direct use of 1,3-dienes for the allylation of ketones via catalytic hydroindation
Source: RSC Adv. 2020 Feb 6;10(10):6030–4. doi: 10.1039/d0ra00853b (PMC9049578; doi:10.1039/d0ra00853b)
Supplement: RA-010-D0RA00853B-s001 [file RA-010-D0RA00853B-s001.pdf]

Electronic Supplementary Material (ESI) for RSC Advances.
This journal is © The Royal Society of Chemistry 2020

Direct Use of 1,3-Dienes for Allylation of Ketones via Catalytic Hydroindation

Supporting Information

Itaru Suzuki,[1] Kensuke Yagi[1,2] Shinji Miyamoto, and Ikuya Shibata*[1]

[1]Research Center for Environmental Preservation, Osaka University, 2-4 Yamadaoka, Suita, Osaka 565-0871, Japan.

[2]Division of Applied Chemistry, Graduate School of Engineering, Osaka University, 2-1 Yamadaoka, Suita, Osaka 565-0871, Japan

e-mail: shibata@epc.osaka-u.ac.jp

Tel: +81-6-6879-8974

1. Analysis

FTIR spectra were recorded as a thin film on a Nicolet IS5 spectrometer. All ^{1}H and ^{13}C NMR spectra were recorded with a JEOL JMTC-400/54/SS (400 and 100 MHz, respectively) in deuteriochloroform ($CDCl_3$) containing 0.03% (w/v) of tetramethylsilane as an internal standard. Yields were determined by ^{1}H NMR using 1,1,1,2-tetrachloroethane or 1,1,2,2-tetrachloroethane as an internal standard. Mass spectra were recorded on a JEOL JMS-DS-303 spectrometer. Flash column chromatography was performed by Yamazen YFLC-AI-580 using Hi-Flash Silica gel 2L Hi-Flash Column 20-3-mL/min eluted by Hexane/EtOAc with gradation mode changing from 9/1 to 3/7 depending on R_f values of each compound. Bulb-to-Bulb distillation (Kugelrohr) was accomplished at the oven temperature and pressure indicated. Purification of products by recycling preparative HPLC was performed by Japan Analytical Industry Co., Ltd. LC-5060.

2. Materials

Super-dehydrated tetrahydrofuran (THF) acetonitrile (MeCN) were purchased from commercial sources and used through GC MINI Solvent Dispensing System (Nikko Hansen & Co., LTD). Dehydrated diethyl ether (Et_2O) and toluene were bought from commercial sources and used as obtained.

Silanes such as Et_3SiH, Ph_3SiH, Ph_2SiH_2, $PhSiH_3$ and $MePhSiH_2$ were purchased from chemical resources and used as obtained.

1,3-Butadiene (**1a**) was purchased from TCI stored in a cylinder and we installed a valve and 20 cm needle to the cylinder. The gas was introduced from the needle into a capped 10 mL graduated cylinder cooled at −40 °C and measured (20 mmol, ca. 1.6 mL). Then it was quickly poured into the reaction system filled with Ar. Other dienes (**1b** and **1c**) were bought from commercial sources and used as obtained.

Vinyl cyclopropane **4** was prepared by our previously reported method.[1]

Other materials we employed throughout this work were purchased from chemical resources and used without any purification.

3. Experimental Procedures

Table 1. In a sealed tube with a septum rubber, $InCl_3$ (0.2 mmol, 44.2 mg) and NaOMe (0.2 mmol, 10.8 mg) were introduced and dried under reduced pressure with heating by a heat-gun followed by refilling with Ar. Then a solvent (2 mL) was poured into the tube and the mixture was stirred at room temperature for 30 min. After a silane (1.1 mmol) was added, the mixture was cooled at −40 °C and liquefied 1,3-butadiene (**1a**, 20 mmol) was poured into the mixture. Then acetophenone (**2a**, 1.0 mmol, 0.120 g) and MeOH (1.0 mmol) were added and the tube was sealed and stirred at indicative temperature for several time. The reaction was quenched with water (10 mL) and extracted with Et_2O (3 x 20 mL). The collected organic layer was dried over $MgSO_4$. After filtration, the filtrate was evaporated in vacuo to give a residue, which was dissolved in $CDCl_3$ and analyzed by ^{1}H NMR.

Table 2. In a sealed tube with a septum rubber, $InCl_3$ (0.2 mmol, 44.2 mg) and NaOMe (0.2 mmol, 10.8 mg) were introduced and dried under reduced pressure with heating by a heat-gun followed by refilling with Ar.

Then THF (2 mL) was poured into the tube and the mixture was stirred at room temperature for 10 min. After $MePhSiH_2$ (1.1 mmol, 0.134 g) was added, the mixture was cooled at −40 °C and liquefied 1,3-butadiene (**1a**, 20 mmol) was poured into the mixture. Then MeOH (1.0 mmol) and ketone **2** (1.0 mmol) were added and the tube was sealed and stirred at 60 °C for 3-6 hours. The reaction was quenched with water (10 mL) and extracted with Et_2O (3 x 20 mL). The collected organic layer was dried over $MgSO_4$. After filtration, the filtrate was evaporated in vacuo to give a residue, which was dissolved in $CDCl_3$ and analyzed by ^1H NMR. Purification was carried out by a silica-gel chromatography to give a pure product. Further purification was performed by distillation under reduced pressure and recycling preparative HPLC to separate stereoisomers.

Scheme 2, ep1. In a sealed tube with a septum rubber, $InCl_3$ (0.2 mmol, 44.2 mg) and NaOMe (0.2 mmol, 10.8 mg) were introduced and dried under reduced pressure with heating by a heat-gun followed by refilling with Ar. Then THF (2 mL) was poured into the tube and the mixture was stirred at room temperature for 10 min. After $MePhSiH_2$ (1.1 mmol, 0.134 g) was added, isoprene (**1b**, 20 mmol) was poured into the mixture. Then MeOH (1.0 mmol) and acetophenone (**2a**, 1.0 mmol, 0.120 g) were added and the tube was sealed and stirred at 60 °C for 48 hours. The reaction was quenched with water (10 mL) and extracted with Et_2O (3 x 20 mL). The collected organic layer was dried over $MgSO_4$. After filtration, the filtrate was evaporated in vacuo to give a residue, which was dissolved in $CDCl_3$ and analyzed by ^1H NMR. Further purification was performed by distillation under reduced pressure and recycling preparative HPLC to separate stereoisomers.

Scheme 2, ep2. In a sealed tube with a septum rubber, $InCl_3$ (0.2 mmol, 44.2 mg) and NaOMe (0.2 mmol, 10.8 mg) were introduced and dried under reduced pressure with heating by a heat-gun followed by refilling with Ar. Then THF (2 mL) was poured into the tube and the mixture was stirred at room temperature for 10 min. After $MePhSiH_2$ (1.1 mmol, 0.134 g) was added, 2,3-dimethyl-1,3-butadiene (**1c**, 20 mmol) was poured into the mixture. Then MeOH (1.0 mmol) and ketone **2** (1.0 mmol) were added and the tube was sealed and stirred at 60 °C for 48 hours. The reaction was quenched with water (10 mL) and extracted with Et_2O (3 x 20 mL). The collected organic layer was dried over $MgSO_4$. After filtration, the filtrate was evaporated in vacuo to give a residue, which was dissolved in $CDCl_3$ and analyzed by ^1H NMR. Further purification was performed by distillation under reduced pressure and recycling preparative HPLC to separate stereoisomers.

Scheme 4, ep1. In a sealed tube with a septum rubber, $InCl_3$ (0.2 mmol, 44.2 mg) and NaOMe (0.2 mmol, 10.8 mg) were introduced and dried under reduced pressure with heating by a heat-gun followed by refilling with Ar. Then THF (2 mL) was poured into the tube and the mixture was stirred at room temperature for 10 min. After $MePhSiH_2$ (1.1 mmol, 0.134 g) was added, 1,3-butadiene (**1a**, 20 mmol) was poured into the mixture. Then CD_3OD or CH_3OH (1.0 mmol) and acetophenone (**2a**, 1.0 mmol, 0.120 g) were added and the tube was sealed and stirred at 60 °C for 30 min. The reaction was quenched with water (10 mL) and extracted with Et_2O (3 x 20 mL). The collected organic layer was dried over $MgSO_4$. After filtration, the filtrate was evaporated in vacuo to give a residue, which was dissolved in $CDCl_3$ and analyzed by ^1H NMR (**3aa-OD**; 25%, **3aa**; 27%).

Scheme 4, ep2. In a sealed tube with a septum rubber, $InCl_3$ (0.2 mmol, 44.2 mg) and NaOMe (0.2 mmol, 10.8 mg) were introduced and dried under reduced pressure with heating by a heat-gun followed by refilling with Ar. Then THF (2 mL) was poured into the tube and the mixture was stirred at room temperature for 10 min. After Ph_2SiD_2 or Ph_2SiH_2 (1.1 mmol) was added, 1,3-butadiene (**1a**, 20 mmol) was poured into the mixture. Then MeOH (1.0 mmol) and acetophenone (**2a**, 1.0 mmol, 0.120 g) were added and the tube was sealed and stirred at 60 °C for 45 min. The reaction was quenched with water (10 mL) and extracted with Et_2O (3 x 20 mL). The collected organic layer was dried over $MgSO_4$. After filtration, the filtrate was evaporated in vacuo to give a residue, which was dissolved in $CDCl_3$ and analyzed by ^1H NMR. The reaction batch was performed three times and the yields were calculated on average (**3aa-CH$_2$D**; 25%, **3aa**; 34%).

The amount of deuterium into the product **3aa-CH$_2$D** was determined by ^1H NMR as shown below. The number of H in f of the product **3aa** is 3. The product **3aa-CH$_2$D** has 2.03 in f. This indicates 0.97D was captured in f.

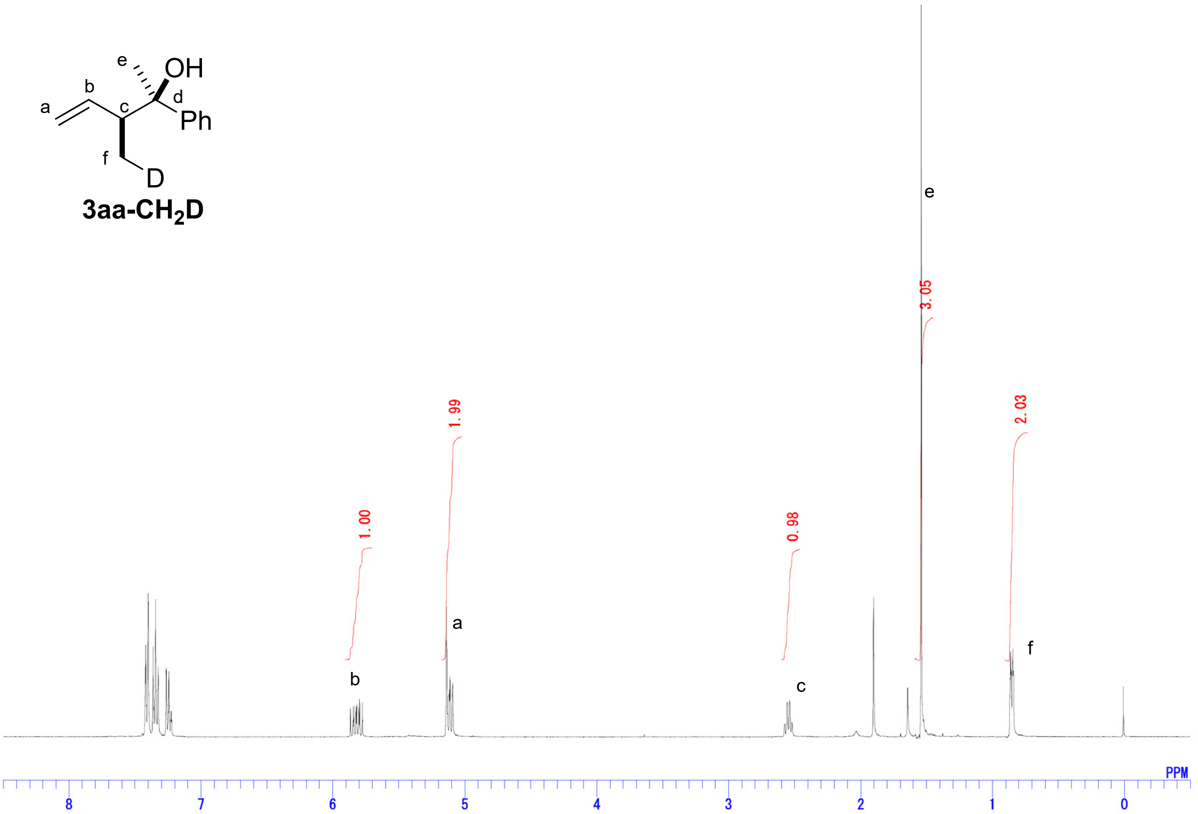

Scheme 5, ep1. In a sealed tube with a septum rubber, $InCl_3$ (0.2 mmol, 44.2 mg) and NaOMe (0.2 mmol, 10.8 mg) were introduced and dried under reduced pressure with heating by a heat-gun followed by refilling with Ar. Then THF (2 mL) was poured into the tube and the mixture was stirred at room temperature for 10 min. After $MePhSiH_2$ (1.1 mmol, 0.134 g) was added, vinyl cyclopropane **4** (2.0 mmol, 0.425 g) was added to the mixture. Then acetophenone (**2a**, 1.0 mmol, 0.120 g), MeOH (1.0 mmol), and V-70 L (0.1 mmol, 30.8 mg) were added and the tube was sealed and stirred at 40 °C for 24 hours. The reaction was quenched with water (10 mL)

and extracted with Et_2O (3 x 20 mL). The collected organic layer was dried over $MgSO_4$. After filtration, the filtrate was evaporated in vacuo to give a residue, which was dissolved in $CDCl_3$ and analyzed by ^1H NMR.

Scheme 5, ep2. According to Buchwald's previous work,[2] $Cu(OAc)_2$ (9.2 mg, 0.05 mmol) and (*S*,*S*)-Ph-BPE (30.4 mg, 0.06 mmol) was charged in a sealed tube and dissolved in toluene (0.3 mL). Then $Me(OMe)_2SiH$ (4.0 mmol, 0.425 g) and vinyl cyclopropane **4** (2.0 mmol, 0.425 g) was added. After cooling at 0 °C, to the mixture was added dropwise acetophenone (**2a**, 1.0 mmol, 0.120 g) dissolved in toluene (1 mL) using a syringe pump and stirred for 12 hours. The reaction was quenched with NH_4F in MeOH (10 mL) and water (10 mL) and extracted with Et_2O (3 x 20 mL). The collected organic layer was dried over $MgSO_4$. After filtration, the filtrate was evaporated in vacuo to give a residue, which was dissolved in $CDCl_3$ and analyzed by ^1H NMR.

4. Product Data

3-Methyl-2-phenylpent-4-en-2-ol (3aa)[3]

Isolated as mixture of diastereomers

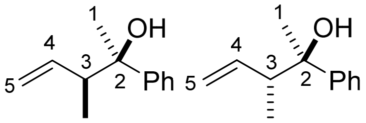

Shape: colorless liquid

^{1}H NMR ($CDCl_3$, 400 MHz) δ7.45-7.40 (m, syn+anti, 2H, Ph), 7.36-7.32 (m, syn+anti, 2H, Ph), 7.26-7.22 (m, syn+anti, 1H, Ph), 5.86-5.77 (m, syn, 1H, $H_2C=C\underline{H}$), 5.74-5.67 (m, anti, 1H, $H_2C=C\underline{H}$)*, 5.14-5.08 (m, syn+anti, 2H, $\underline{H_2}C=CH$), 2.62-2.52 (m, 1H, $H_2C=CHC\underline{H}$), 1.98 (s, anti, 1H, OH)*, 1.87 (s, 1H, syn, OH), 1.53 (s, syn+anti, 3H, $C\underline{H_3}COH$), 0.97 (d, J = 6.8 Hz, anti, 3H, $H_2C=CHCC\underline{H_3}$)*, 0.86 (d, J = 7.0 Hz, syn, 3H, $H_2C=CHCHC\underline{H_3}$)

^{13}C NMR ($CDCl_3$, 100 MHz) δ146.9, 146.8*, 139.8 (syn+anti), 127.9, 127.8*, 126.5*, 126.4, 125.4*, 125.2, 116.4*, 116.3, 75.8, 75.6*, 48.9, 48.6*, 28.5, 25.8*, 14.7, 14.0*

* Mark corresponds to anti isomer.

MS (CI, 200 eV) m/z 159 (521), 160 (72), 121 (41)

HRMS calcd for $[C_{12}H_{16}O\text{-}OH]^+$: 159.1168, found: m/z 159.1173 (CI)

^{1}H NMR (syn isomer)

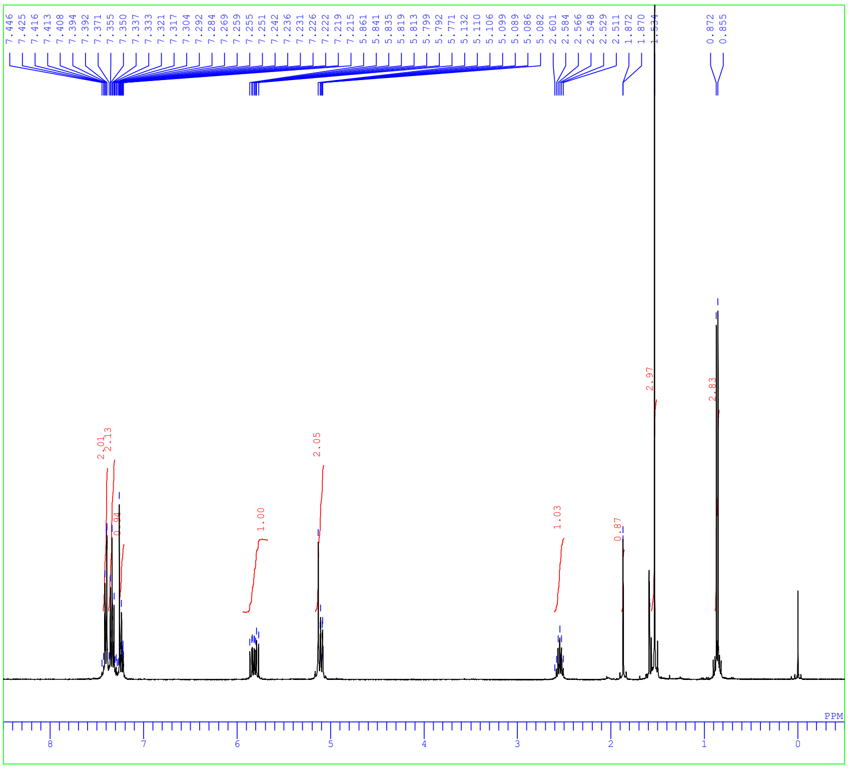

^{13}C NMR (syn isomer)

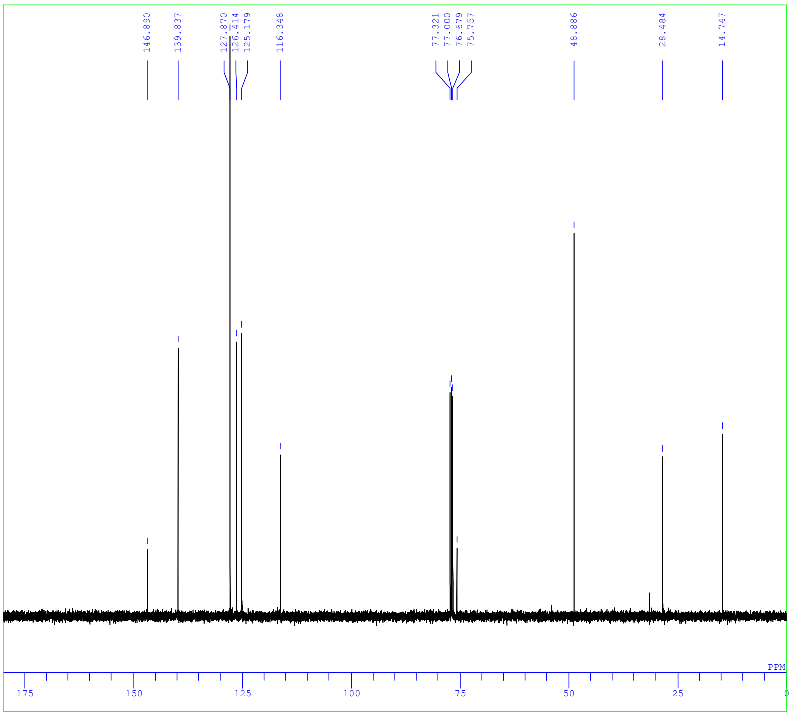

^{1}H NMR (syn+anti isomer)

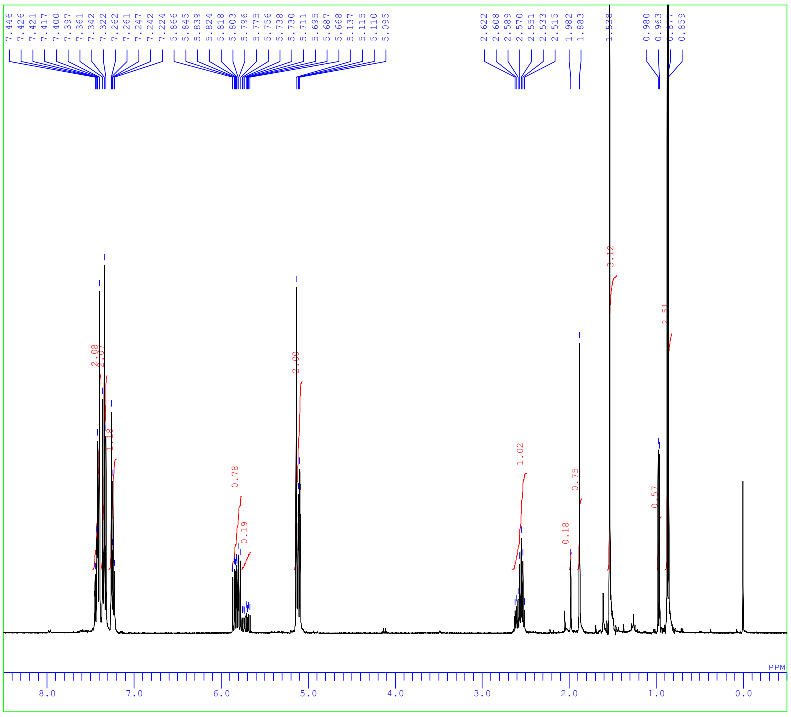

^{13}C NMR (syn+anti isomer)

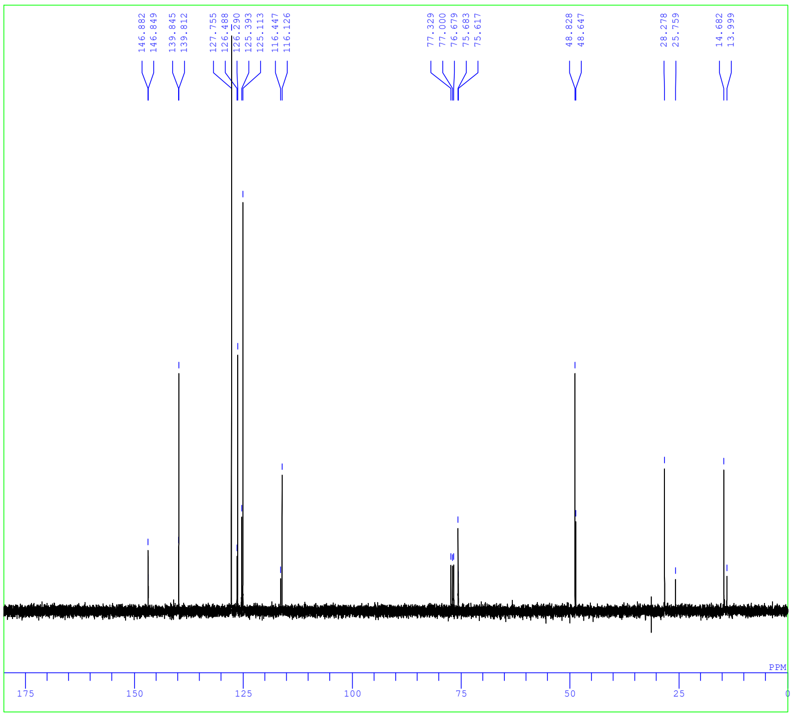

2-(4-Chlorophenyl)-3-methylpent-4-en-2-ol (3ab)[4]

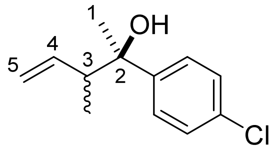

known compound

^{1}H NMR (CDCl$_3$, 400 MHz) δ7.37-7.28 (m, syn+anti, 4H, Ph), 5.84-5.75 (m, syn, 1H, H$_2$C=C*H*), 5.72-5.63 (m, anti, 1H, H$_2$C=C*H*)*, 5.15-5.08 (m, syn+anti, 2H, *H*$_2$C=CH), 2.58-2.47 (m, syn+anti, 1H, CH$_3$C*H*), 1.97 (s, anti, 1H, OH)*, 1.85 (s, 1H, syn, OH), 1.51 (s, syn+anti, 3H, C*H*$_3$COH), 0.96 (d, *J*= 7.0 Hz, anti, 3H, C*H*$_3$CH)*, 0.85 (d, *J*= 6.8 Hz, syn, 3H, C*H*$_3$CH)

^{13}C NMR (CDCl$_3$, 100 MHz) δ145.4, 145.3*, 139.4, 139.3*, 132.2, 132.0*, 127.9 (syn+anti), 127.0*, 126.7, 116.8*, 116.6, 75.5, 75.4*, 48.8 (syn+anti), 28.4, 25.7*, 14.6, 14.0*

MS (CI, 200 eV) m/z 193 (645), 195 (200), 194 (76), 155 (42), 196 (30)

HRMS calcd for [$C_{12}H_{15}OCl$-OH]$^+$: 193.0779, found: m/z 193.0787 (CI)

^{1}H NMR (major isomer)

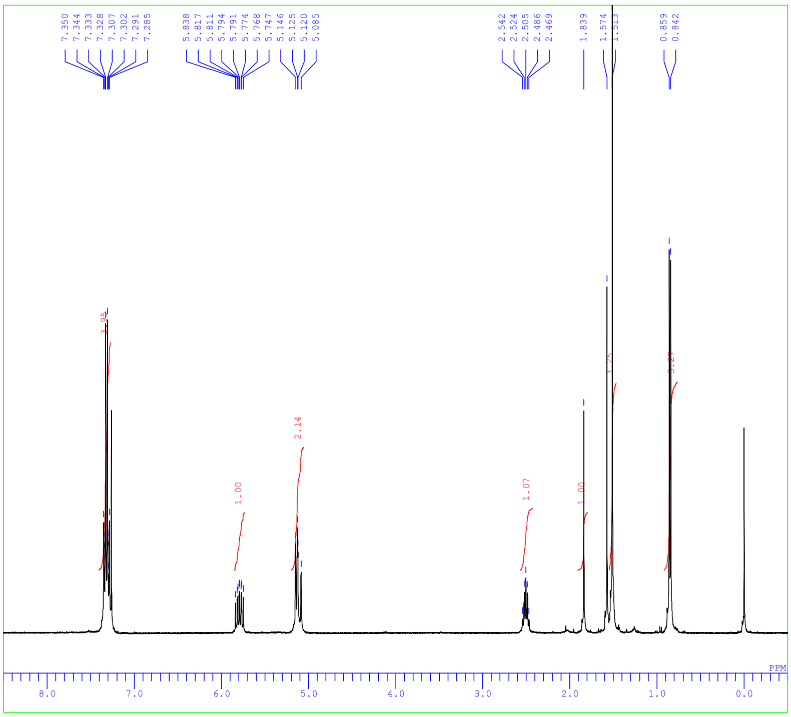

^{1}H NMR (major+minor isomer)

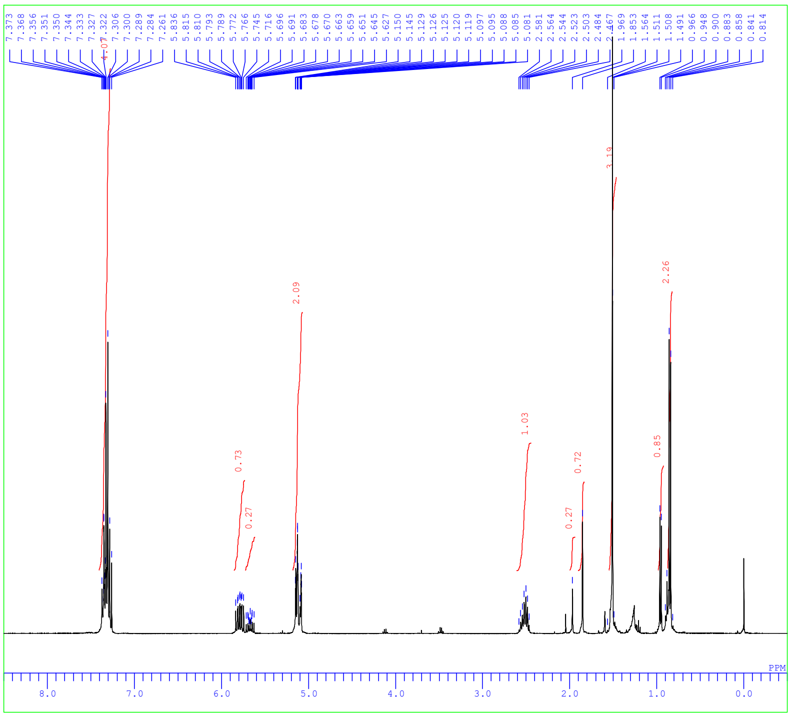

^{13}C NMR (major isomer)

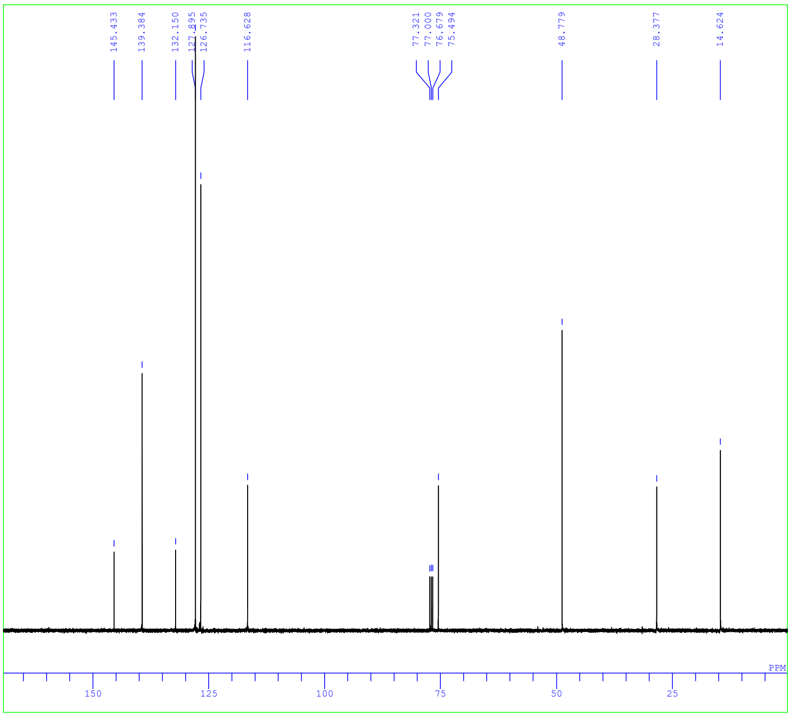

^{13}C NMR (major+minor isomer)

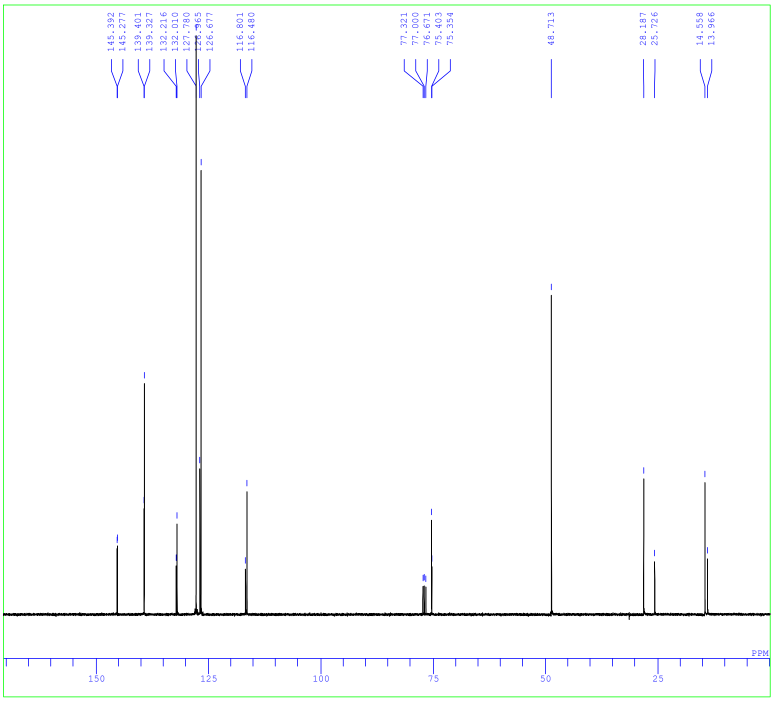

2-(4-Bromophenyl)-3-methylpent-4-en-2-ol (3ac)[5]

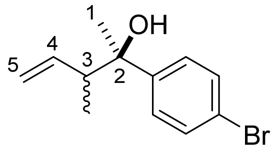

^{1}H NMR ($CDCl_3$, 400 MHz) δ7.46-7.26 (m, 4H, Ph, syn+anti), 5.84- 5.75 (m, 1H, H_2C=C*H*, syn), 5.70- 5.62 (m, 1H, *H_2*C=CH, anti)*, 5.15-5.01 (m, 2H, *H_2*C=CH, syn+anti), 2.59- 2.46 (m, 1H, CH_3C*H*, syn+anti), 1.97 (br, 1H, OH, anti)*, 1.87 (br, 1H, OH, syn), 1.50 (s, 3H, C*H_3*COH, syn+anti), 0.96 (d, J = 6.9 Hz, 3H, C*H_3*CH, anti)*, 0.85 (d, J = 7.0 Hz, 3H, C*H_3*CH, syn)

^{13}C NMR ($CDCl_3$, 100 MHz) δ146.0, 139.4, 130.9, 127.4*, 127.1, 120.6*, 120.4, 117.1*, 116.8, 75.5, 48.7, 28.5, 25.8*, 14.6, 14.0* (Five peaks overlap)

* Mark corresponds to anti isomer.

MS (CI, 200 eV) m/z 237 (325), 239 (306), 240 (41), 201 (21)

HRMS calcd for $[C_{12}H_{15}OBr\text{-}OH]^+$: 237.0273, found: m/z 237.0273 (CI)

^{1}H NMR (syn isomer)

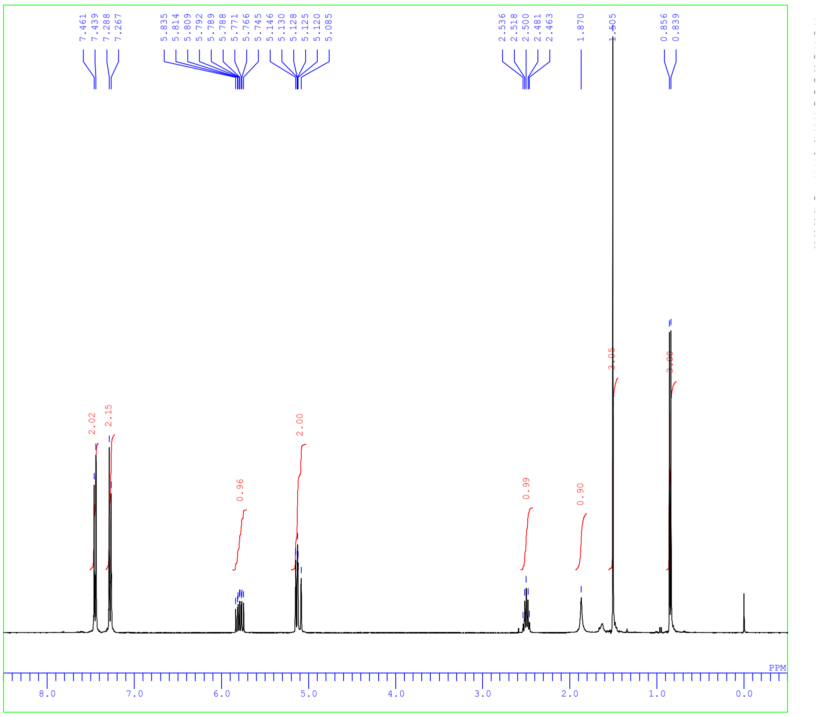

^{1}H NMR (syn+anti isomer)

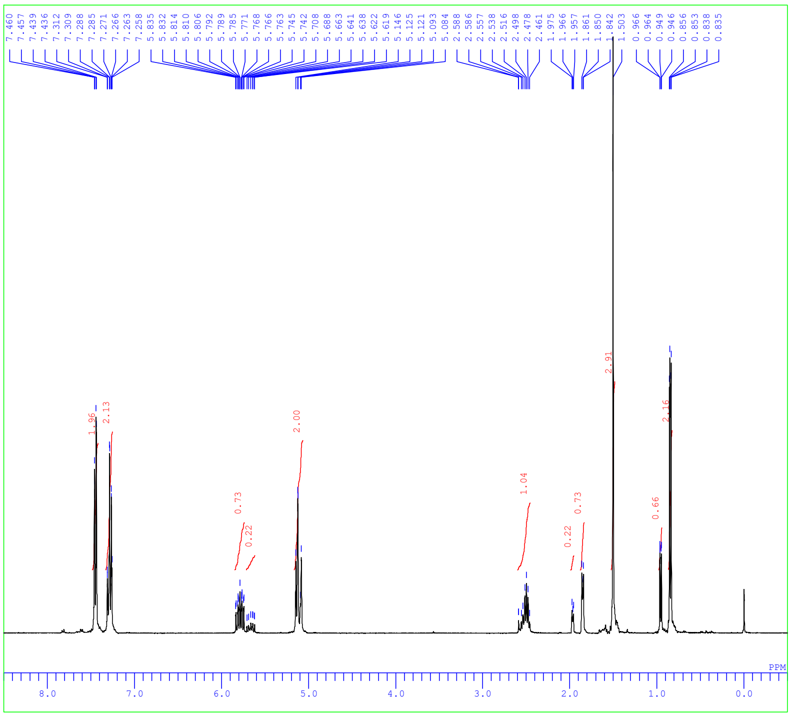

^{13}C NMR (syn isomer)

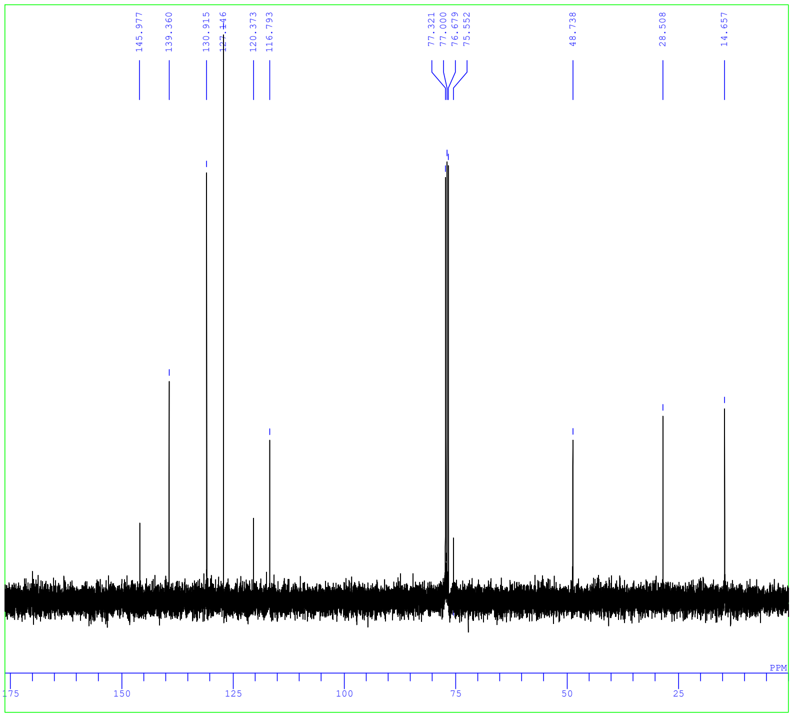

^{13}C NMR (syn+anti isomer)

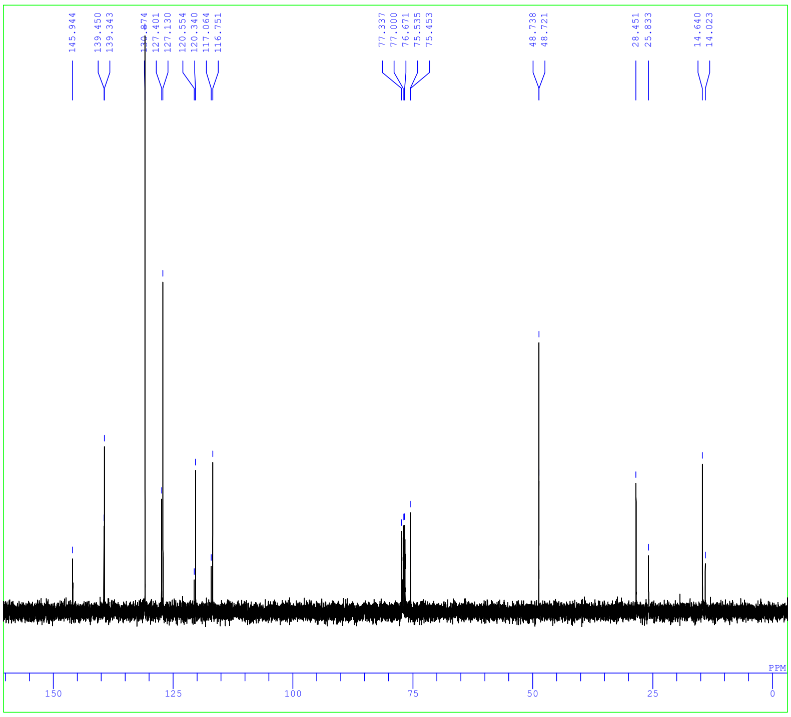

4-(2-Hydroxy-3-methylpent-4-en-2-yl)benzonitrile (3ad)

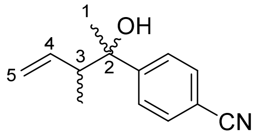

major isomer

^{1}H NMR ($CDCl_3$, 400 MHz) δ7.63 (d, J = 8.7 Hz, 2H, Ph), 7.53 (d, J = 8.7 Hz, 2H, Ph), 5.86-5.77 (m, 1H, $H_2C=C\underline{H}$), 5.19-5.11 (m, 2H, $\underline{H_2}C=CH$), 2.57-2.50 (m, 1H, $CH_3C\underline{H}$), 1.89 (s, 1H, OH), 1.54 (s, 3H, $C\underline{H_3}COH$), 0.83 (d, J = 7.0 Hz, 3H, $C\underline{H_3}CH$)

^{13}C NMR ($CDCl_3$, 100 MHz) : δ152.5, 138.8, 131.7, 126.1, 118.9, 117.2, 110.2, 75.7, 48.6, 28.5, 14.5

bp: 110 °C /0.29 torr

minor isomer

^{1}H NMR (CDCl3, 400 MHz) δ7.63 (d, J = 8.7 Hz, 2H, Ph), 7.55 (d, J = 8.7 Hz, 2H, Ph), 5.67-5.58 (m, 1H, $H_2C=C\underline{H}$), 5.12-5.07 (m, 2H, $\underline{H_2}C=CH$), 2.60-2.52 (m, 1H, $CH_3C\underline{H}$), 2.10 (s, 1H, OH), 1.54 (s, 3H, $C\underline{H_3}COH$),0.99 (d, J = 6.8 Hz, 3H, $C\underline{H_3}CH$)

^{13}C NMR ($CDCl_3$, 100 MHz) δ152.5, 138.9, 131.7, 126.4, 118.9, 117.5, 110.4, 75.6, 48.7, 26.0, 13.9

Properties of mixture of diastereomers: IR (neat) 3491 cm^{-1} (OH), 2228 cm^{-1} (CN)

MS (CI, 200 eV) m/z 202 (M+1), 205, 193

HRMS: (CI+, 200 eV) Calculated ($C_{13}H_{16}NO$) 202.1232 (M+1) Found: 202.1235

^{1}H NMR (major isomer)

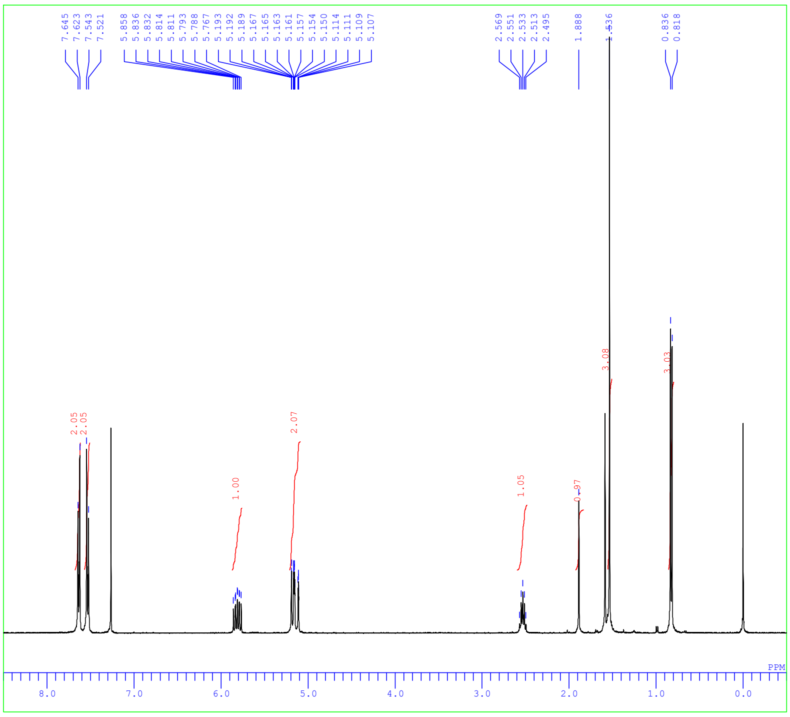

^{13}C NMR (major isomer)

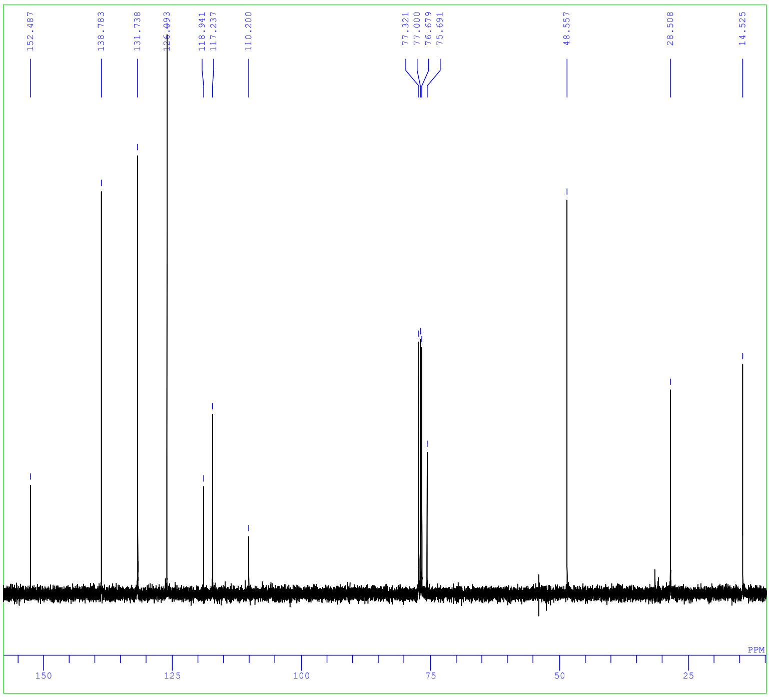

^{1}H NMR (minor isomer)

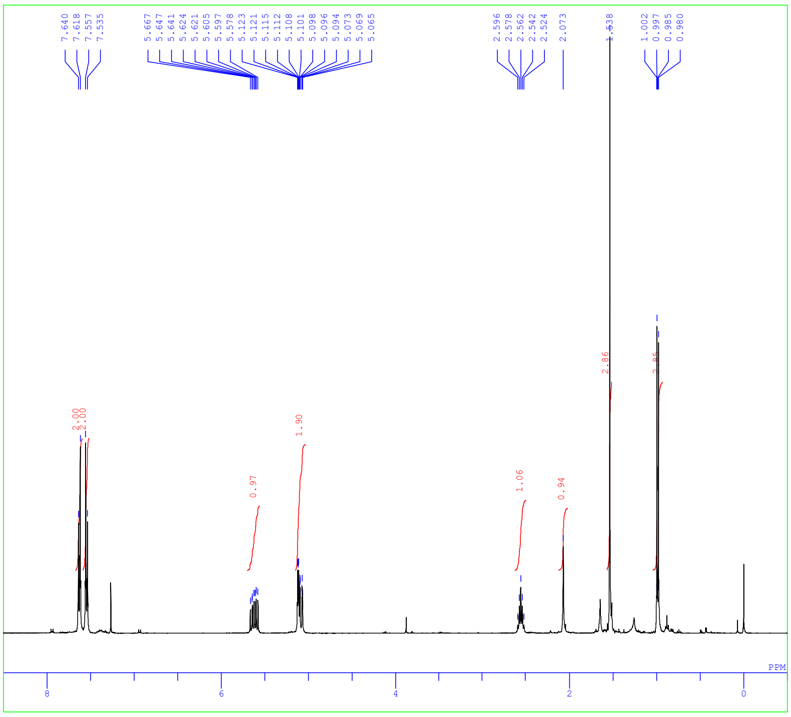

^{13}C NMR (minor isomer)

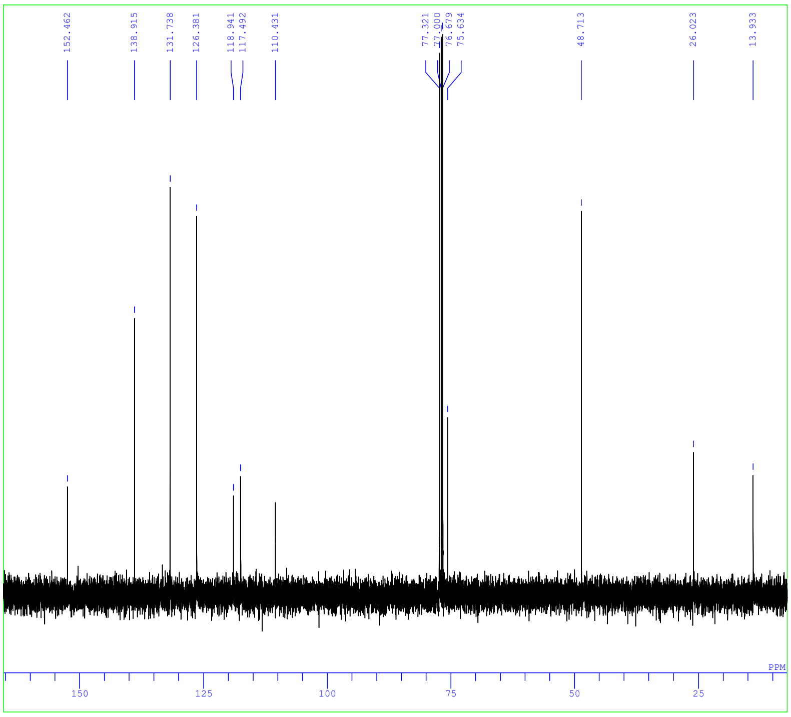

3-Methyl-2-(p-tolyl)pent-4-en-2-ol (3ae)

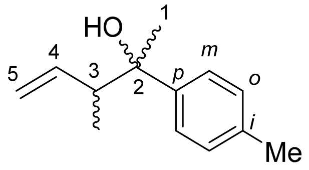

colorless liquid

IR (neat) 3471 cm^{-1} (OH), bp: 105 °C /0.25 torr

MS (CI, 200 eV) m/z 191 (M + 1), 173 (100)

HRMS: (CI+, 200 eV) Calculated ($C_{13}H_{19}O$) 191.1436 (M +1) Found: 191.1436

Anal. calcd for $C_{13}H_{18}O$: C, 82.06; H, 9.54 found: C, 81.71; H, 9.55;

^{1}H NMR ($CDCl_3$, 400 MHz) : δ7.33–7.26 (m, major+minor, 2H, Ph), 7.15-7.13 (m, major+minor , 2H, Ph), 5.85-5.67 (m, major+minor, 1H, $H_2C{=}C\underline{H}$), 5.13-5.07(m, major+minor, 2H, $\underline{H}_2C{=}CH$), 2.62-2.49 (m, major+minor, 1H, $CH_3C\underline{H}$), 2.34 (s, major+minor, 3H, $C_6H_4C\underline{H}_3$), 1.94 (s, minor, OH), 1.83 (s, 1H, OH), 1.51 (s, major+minor, 3H,$C\underline{H}_3COH$), 0.96 (d, minor, J = 6.8 Hz, 3H, $C\underline{H}_3CH$), 0.87 (d, J = 7.0 Hz, 3H, $C\underline{H}_3CH$)

^{13}C NMR ($CDCl_3$, 100 Hz): δ144.0 (major+minor), 140.0 (major+minor), 136.0*, 135.8, 128.5 (major+minor), 125.4*, 125.1, 116.4*, 116.1, 75.6 (major+minor) , 50.0, 48.7*, 28.4, 25.9*, 20.9 (major+mino r), 14.8, 14.1*

^{1}H NMR (major isomer)

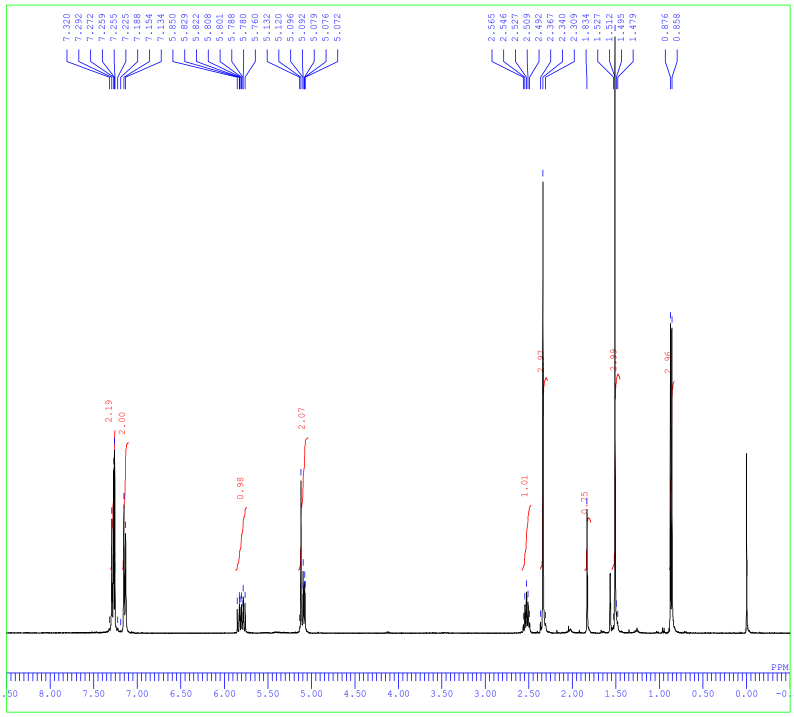

^{13}C NMR (major isomer)

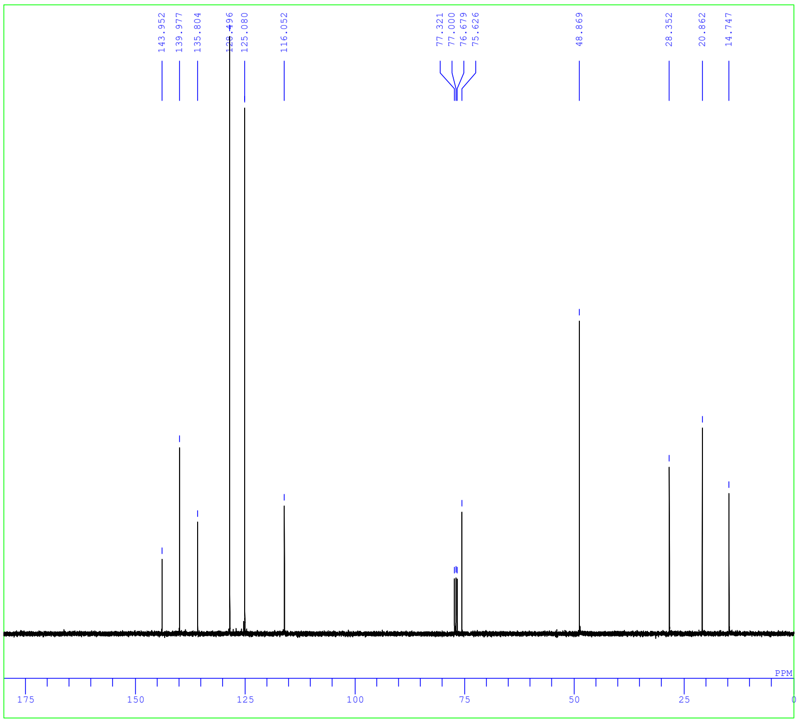

^{1}H NMR (major+minor isomer)

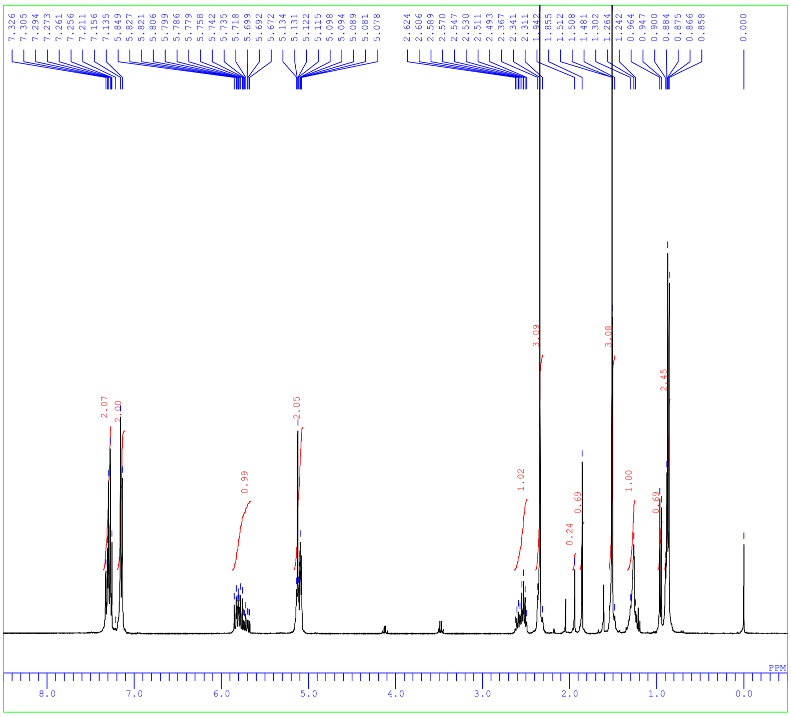

^{13}C NMR (major+minor isomer)

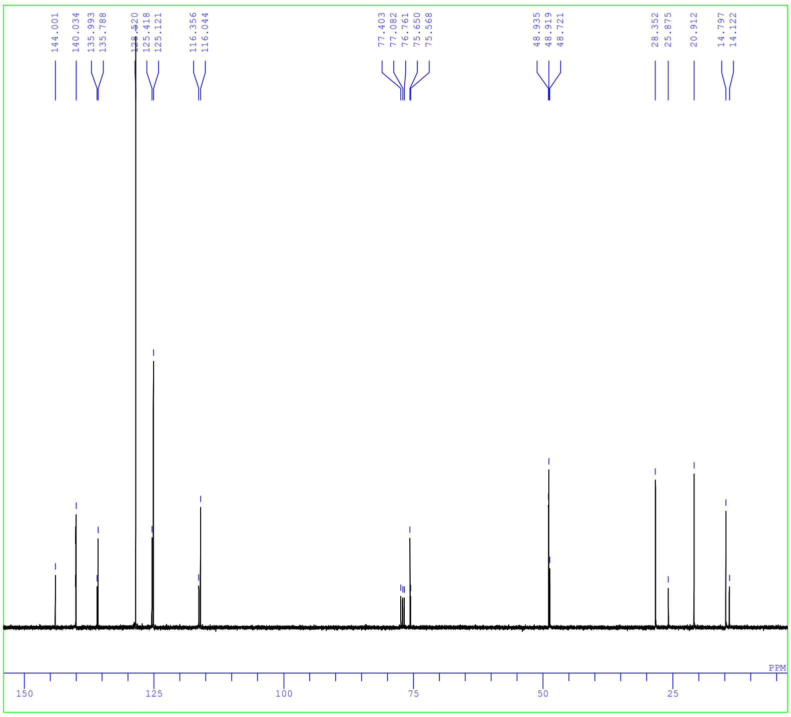

2-(4-Methoxyphenyl)-3-methylpent-4-en-2-ol (3af)[4]

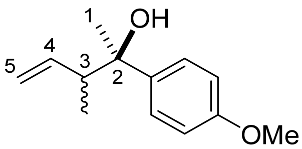

major isomer

^{1}H NMR ($CDCl_3$, 400 MHz) δ 7.33-7.30 (m, 2H, Ph), 6.89-6.86 (m, 2H, Ph), 5.83-5.74 (m, 1H, $H_2C{=}C\underline{H}$), 5.11-5.01 (m, 2H, $\underline{H}_2C{=}CH$), 3.81 (s, 3H, OMe), 2.55-2.48 (m, 1H, $C\underline{H}_3CH$), 1.85 (s, 1H, OH), 1.51 (s, 3H, $C\underline{H}_3COH$), 0.88 (d, J = 6.8 Hz, 3H, $C\underline{H}_3CH$)

^{13}C NMR ($CDCl_3$,100 Hz) δ158.0, 140.0, 139.0, 126.4, 116.2, 113.1, 75.4, 55.2, 49.0, 28.3, 14.8

minor isomer

^{1}H NMR ($CDCl_3$, 400 MHz) δ7.37-7.33 (m, 2H, Ph), 6.89-6.85 (m, 2H, Ph), 5.72-5.68 (m, 1H, $H_2C{=}C\underline{H}$), 5.15-5.01 (m, 2H, $\underline{H}_2C{=}CH$), 3.81 (s, 3H, OMe), 2.60-2.53 (m, 1H, $CH_3C\underline{H}$), 1.93 (s, 1H, OH), 1.51 (s, 3H, $C\underline{H}_3COH$), 0.94 (d, J = 6.8 Hz, 3H, $C\underline{H}_3CH$)

^{13}C NMR ($CDCl_3$, 100 MHz) δ158.1, 140.1, 139.1, 126.6, 116.5, 113.1, 75.4, 55.1, 49.0, 25.7, 14.3

MS (CI, 200 eV) m/z 189 (250), 245 (38), 190 (37)

HRMS calcd for $[C_{13}H_{18}O_2\text{-}OH]^+$: 189.1274, found: m/z 189.1280 (CI)

^{1}H NMR (major isomer)

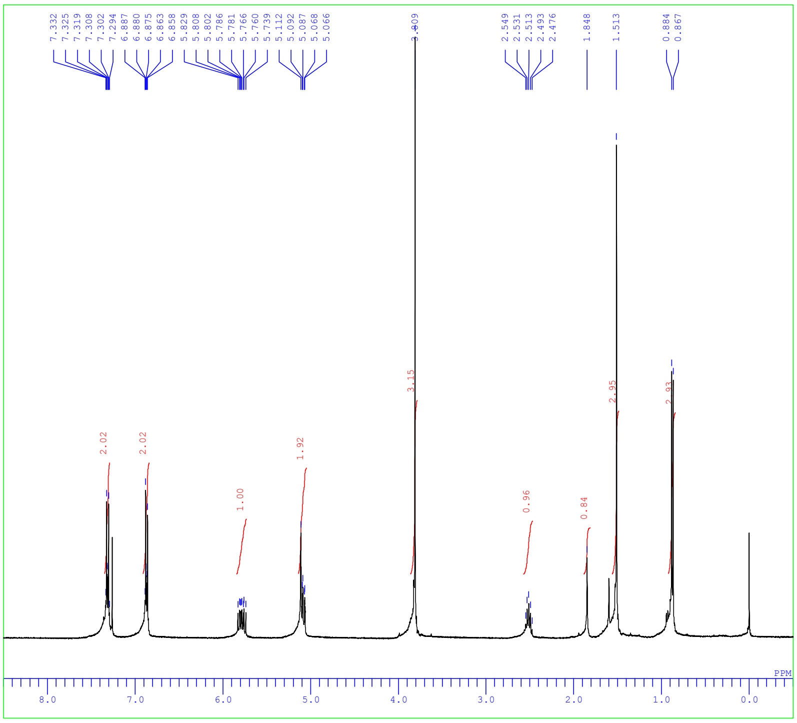

^{13}C NMR (major isomer)

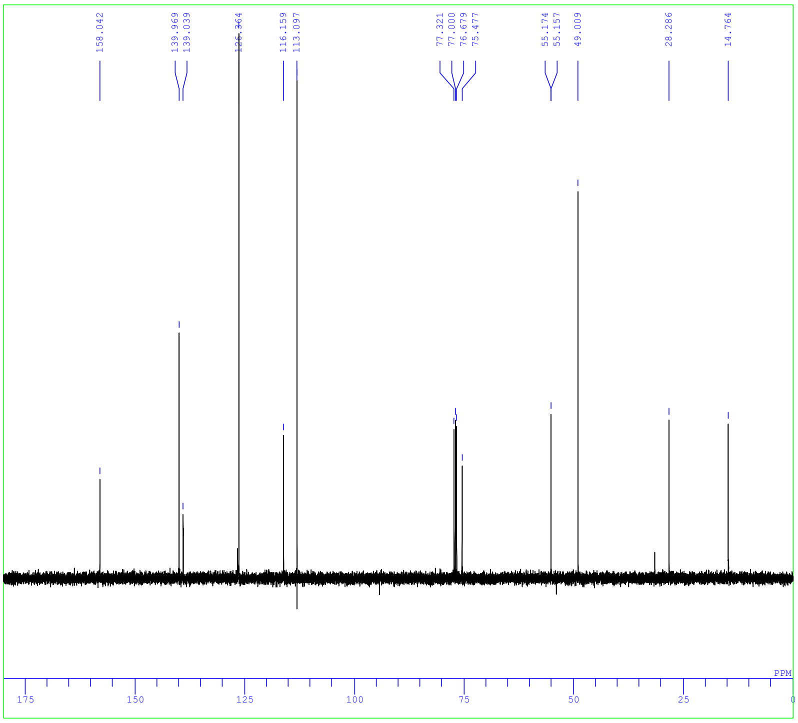

^{1}H NMR (minor isomer)

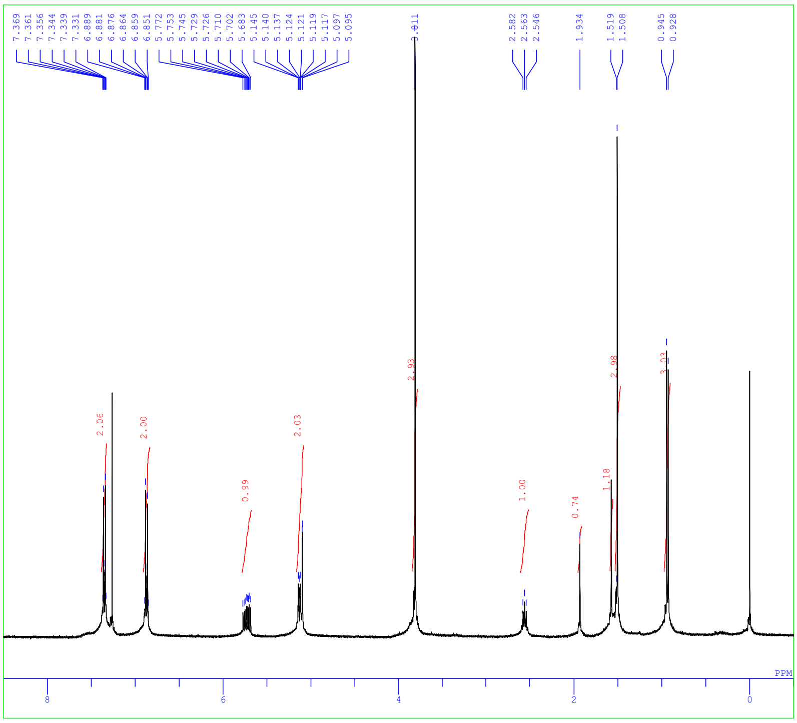

^{13}C NMR (minor isomer)

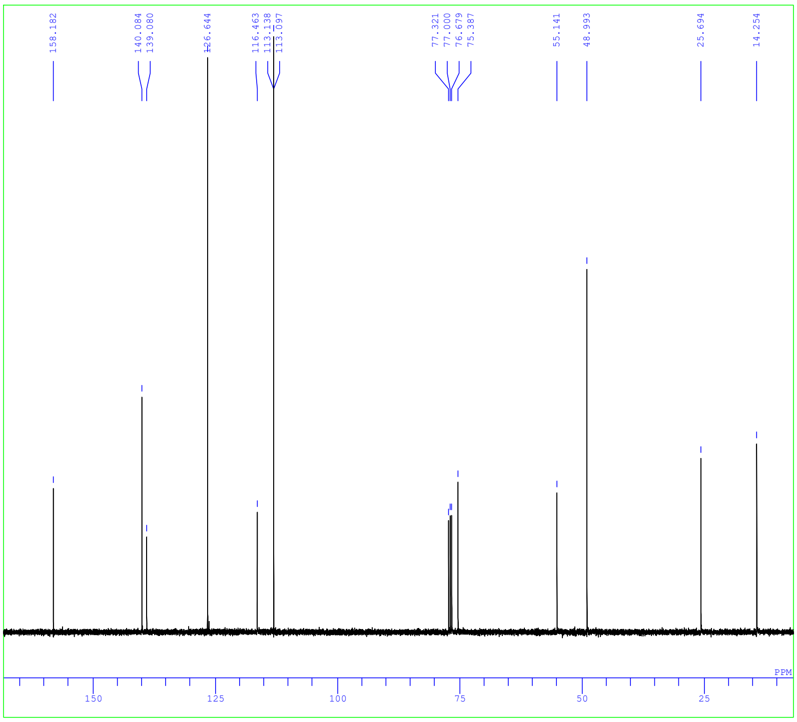

4-Methyl-3-phenylhex-5-en-3-ol (3ag)[6]

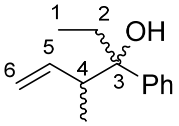

major isomer

^{1}H NMR ($CDCl_3$, 400 MHz) δ7.36-7.31 (m, 4H, Ph), 7.25-7.21 (m, 1H, Ph), 5.89-5.80 (m, 1H, $H_2C{=}C\underline{H}$), 5.16-5.11 (m, 2H, $\underline{H}_2C{=}CH$), 2.61-2.54 (m, 1H, $H_2C{=}CHC\underline{H}$), 1.95- 1.82 (m, 2H, $CH_3C\underline{H}_2$), 1.80 (s, 1H, OH), 0.81 (d, J = 7.0 Hz, 3H, $H_2C{=}CHCHC\underline{H}_3$), 0.68 (t, J = 7.4 Hz, 3H, $C\underline{H}_3CH_2$)

^{13}C NMR ($CDCl_3$, 100 MHz) δ144.3, 140.0, 127.7, 126.1, 125.7, 116.2, 78.3, 48.2, 33.3, 14.9, 7.7

minor isomer

^{1}H NMR ($CDCl_3$, 400 MHz) δ7.39-7.31 (m, 4H, Ph), 7.26-7.21 (m, 1H, Ph), 5.67-5.58 (m, 1H, $H_2C{=}C\underline{H}$), 5.08-5.03 (m, 2H, $\underline{H}_2C{=}CH$), 2.69-2.62 (m, 1H, $H_2C{=}CHC\underline{H}$), 2.02- 1.93 (m, 1H, $CH_3C\underline{H}_2$), 1.88- 1.79 (m, 1H, $CH_3C\underline{H}_2$), 1.81 (s, 1H, OH), 1.03 (d, J = 6.8 Hz, 3H, $H_2C{=}CHCHC\underline{H}_3$), 0.73 (t, J = 7.4 Hz, 3H, $C\underline{H}_3CH_2$)

^{13}C NMR ($CDCl_3$, 100 MHz) δ144.6, 140.0, 127.7, 126.3, 126.1, 116.3, 78.1, 47.5, 31.3, 13.3, 7.8

MS (CI, 200 eV) m/z 173 (622), 174 (80), 135 (39)

HRMS calcd for $[C_{13}H_{18}O\text{-}OH]^+$: 173.1325, found: m/z 173.1331 (CI)

^{1}H NMR (major isomer)

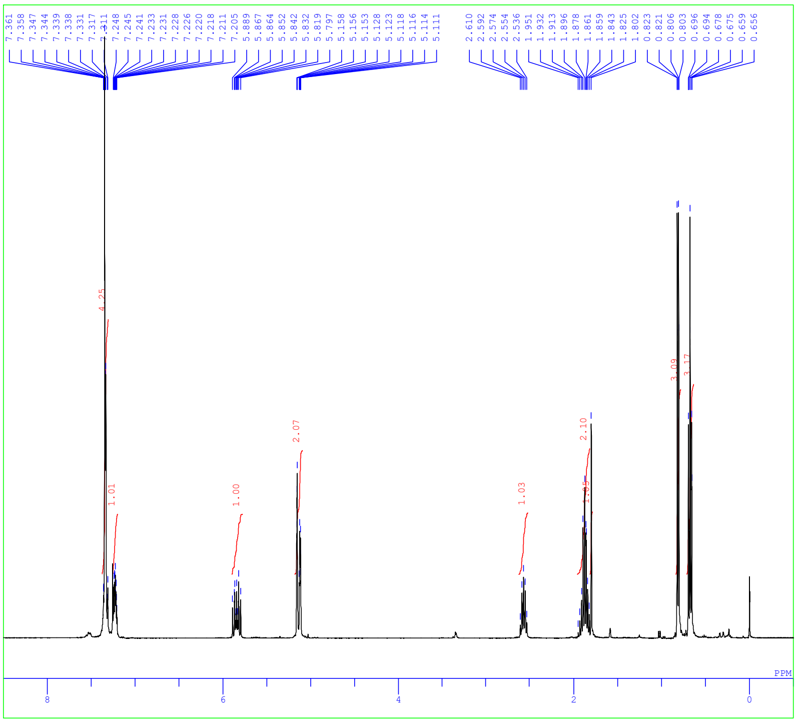

^{13}C NMR (major isomer)

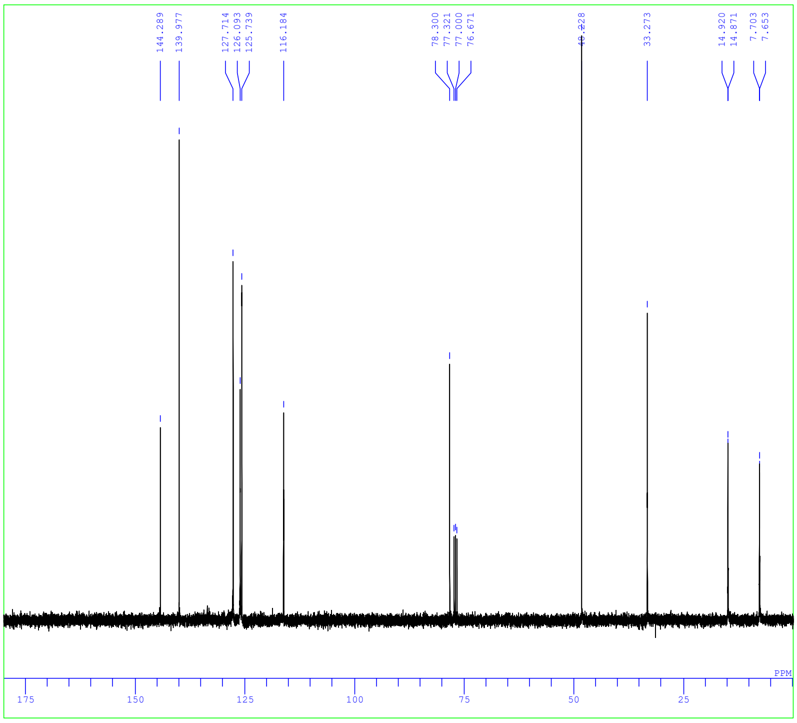

^{1}H NMR (minor isomer)

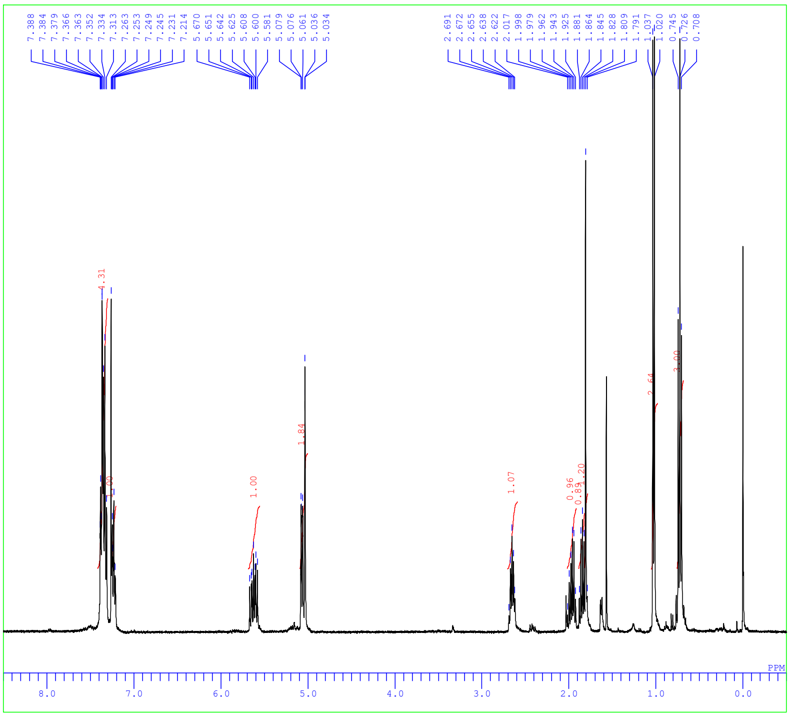

^{13}C NMR **(**minor isomer)

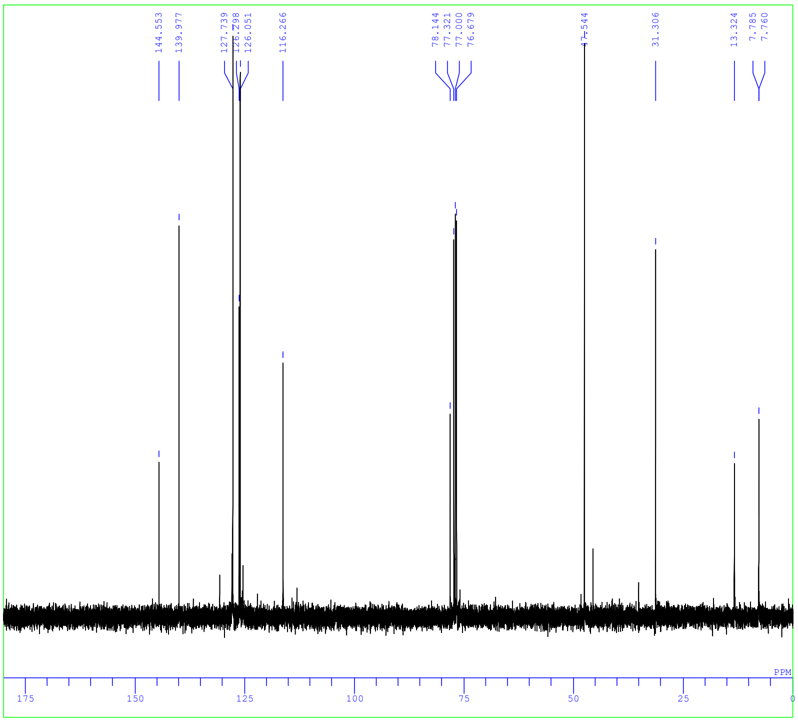

3-Methyl-4-phenylhept-1-en-4-ol (3ah)

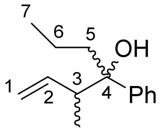

Isolated as mixture of diastereomers

colorless liquid

IR (neat) 3507 cm^{-1} (OH) bp: 90 °C /0.23 torr

^{1}H NMR ($CDCl_3$, 400 MHz) δ7.37-7.31 (m, majo+minor, 4H, Ph), 7.26-7.19 (m, major+minor, 1H, Ph), 5.89-5.80 (m, major, 2H, $H_2C{=}C\underline{H}$), 5.66-5.57 (m, minor, 1H, $H_2C{=}C\underline{H}$)*, 5.16-5.12 (m, major, 2H, $\underline{H}_2C{=}CH$), 5.07-5.03 (m, minor, 2H, $\underline{H}_2C{=}CH$)*, 2.68-2.63 (m, minor, 1H, $H_2C{=}CHC\underline{H}$)*, 2.60-2.52 (m, major, 1H, $H_2C{=}CHC\underline{H}$), 1.93-1.72 (m, major+minor, 2H+1H, $CH_3CH_2C\underline{H}_2$ or $CH_3C\underline{H}_2$+OH), 1.32-1.18 (m, major+minor, 1H, $CH_3CH_2C\underline{H}_2$ or $CH_3C\underline{H}_2$+OH), 1.03 (d, J = 7.0 Hz, 1H, minor, $C\underline{H}_3CH$), 0.99-0.89 (m, major+minor, 1H, $COHC\underline{H}_2$ or $CH_3C\underline{H}_2$), 0.87-0.80 (m, major+minor, 6H, $C\underline{H}_3CH_2$+$C\underline{H}_3CH_2$)

^{13}C NMR ($CDCl_3$, 100 MHz) δ145.0*, 144.8, 140.0 (major+minor), 127.8, 127.7*, 126.2*, 126.1, 125.8*, 125.6, 116.2 (major+minor), 78.1, 77.9*, 48.5, 47.7*, 43.2, 41.2*, 16.7 (major+minor), 14.8, 14.4 (major+minor), 13.2*

* Mark corresponds to minor isomer.

HRMS calcd for $C_{14}H_{21}O$: 205.1592, found: m/z 205.1589 (CI, M^++1, +0.3 mmu)

MS: (CI+, 70 eV) m/z 204 (M^++1), 187 (100)

^{1}H NMR (major isomer)

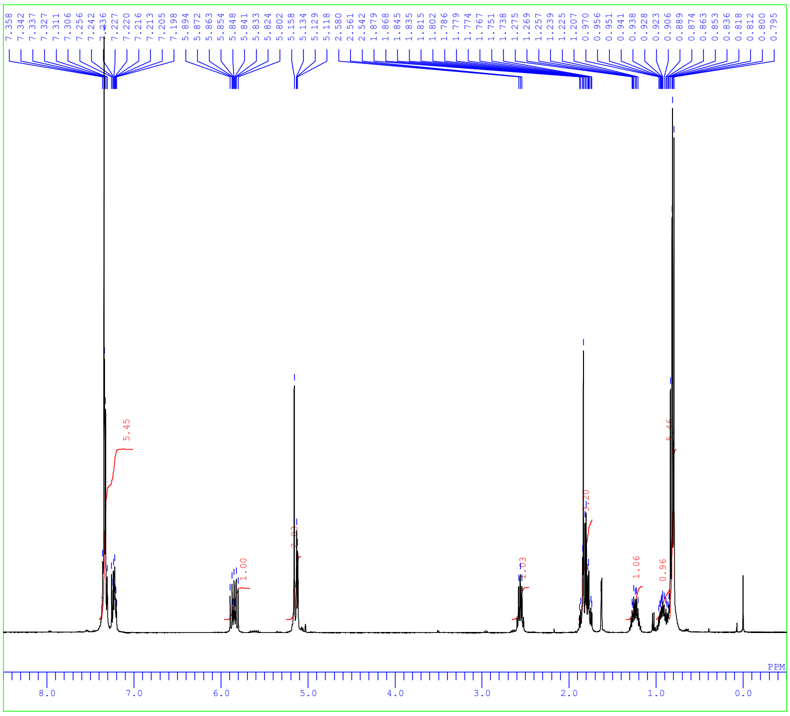

^{1}H NMR (major+minor isomer)

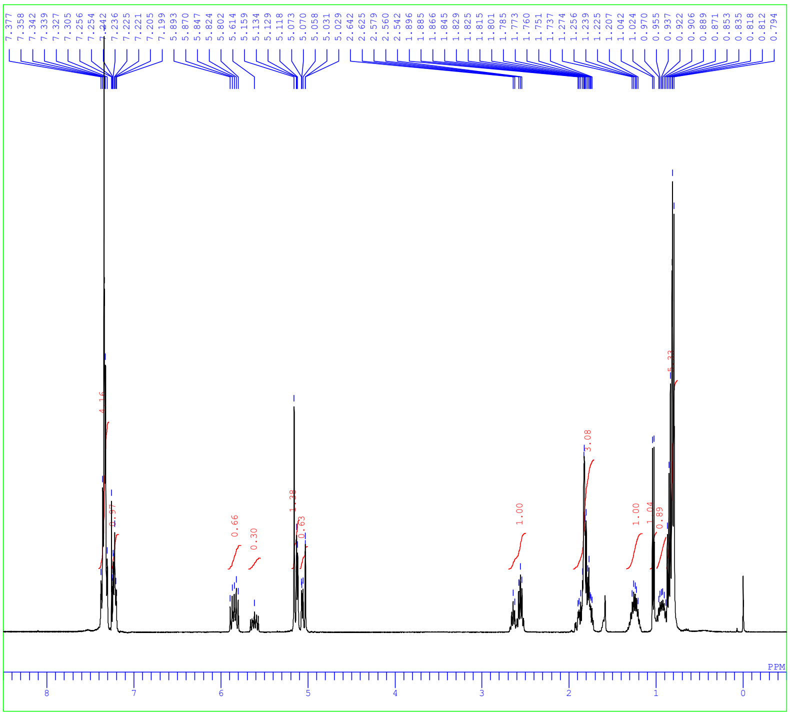

^{13}C NMR (major isomer)

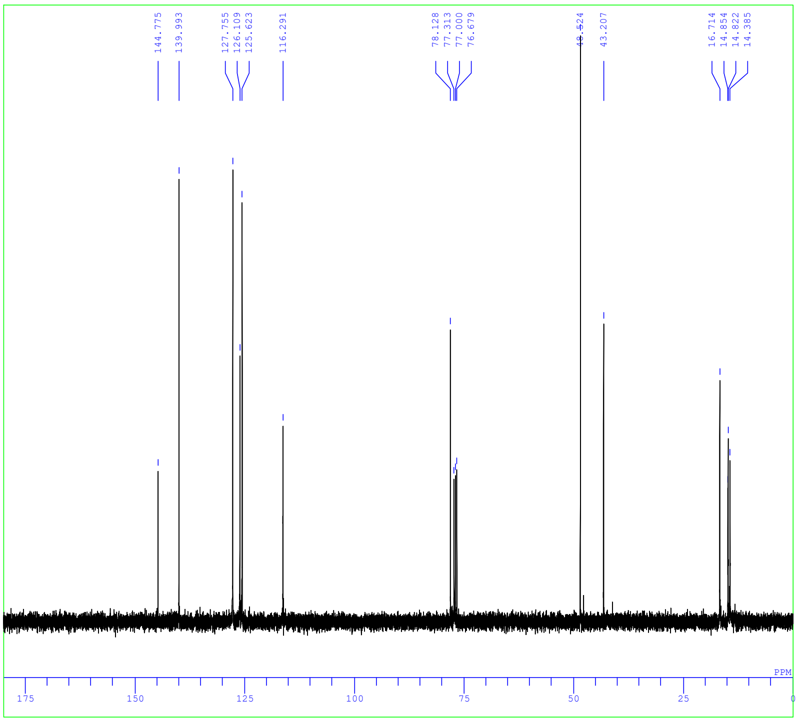

^{13}C NMR (major+minor isomer)

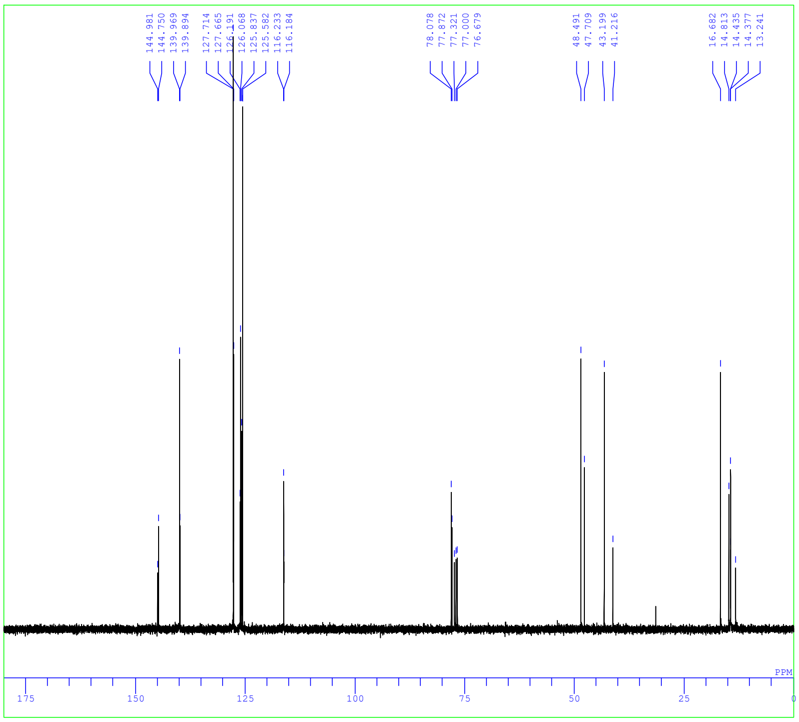

3-Hydroxy-4-methyl-3-phenyl-hex-5-enenitrile (3ai)

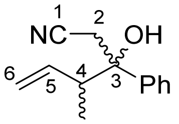

Isolate as mixture of diastereomers

colorless liquid

IR (neat) 2255 cm^{-1} (CN), 3548 cm^{-1} (OH)

MS (CI, 200eV) m/z 202 (M^{+}+1, 655), bp: 116 °C /1.4 torr

HRMS calcd for $C_{13}H_{15}NO$: 201.1154, found: m/z 202.1234 (CI, M^{+}+1, +0.2 mmu)

^{1}H NMR ($CDCl_3$, 400 MHz) δ7.43–7.31 (m, 5H, Ph, major+minor), 5.84- 5.75 (m, 1H, H_2C=C$\underline{H}$, major), 5.60-5.51 (m, 1H, H_2C=C$\underline{H}$, minor)*, 5.28-5.23 (m, 2H, $\underline{H_2}$C=CH, major), 5.18- 5.14 (m, 2H, $\underline{H_2}$C=CH, minor)*, 2.94 (m, 1H, C$\underline{H_2}$CN, minor), 2.89 (m, 1H, C$\underline{H_2}$CN, major), 2.80-2.71 (m, 1H, CH_3C$\underline{H}$, major+minor), 2.51-2.49 (br, 1H, OH, minor)*, 2.36 (s, 1H, OH, major), 0.99 (d, *J*=7.0 Hz, 3H, CH_3, minor)*, 0.88 (d, *J*=7.0 Hz, 3H, CH_3, major)

^{13}C NMR ($CDCl_3$, 100 MHz) δ142.1, 138.2*, 138.0, 128.5, 128.2*, 127.8*, 127.8, 125.5*, 125.1, 118.2, 117.8*, 117.6*, 117.3, 76.2, 76.1*, 47.3, 47.0*, 31.1, 29.7*, 14.8, 13.6*

* Mark corresponds to minor isomer.

^{1}H NMR (major isomer)

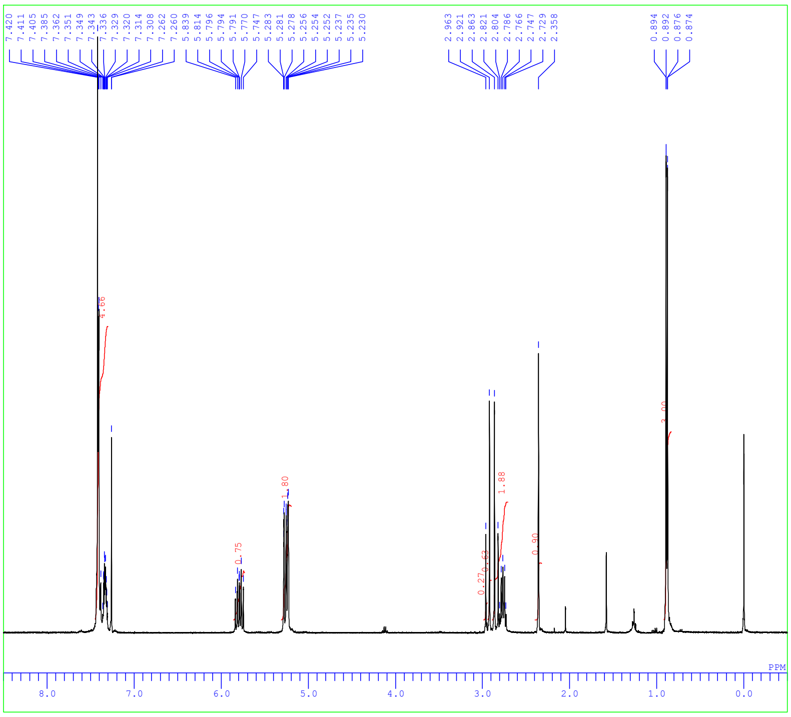

^{1}H NMR (major+minor isomer)

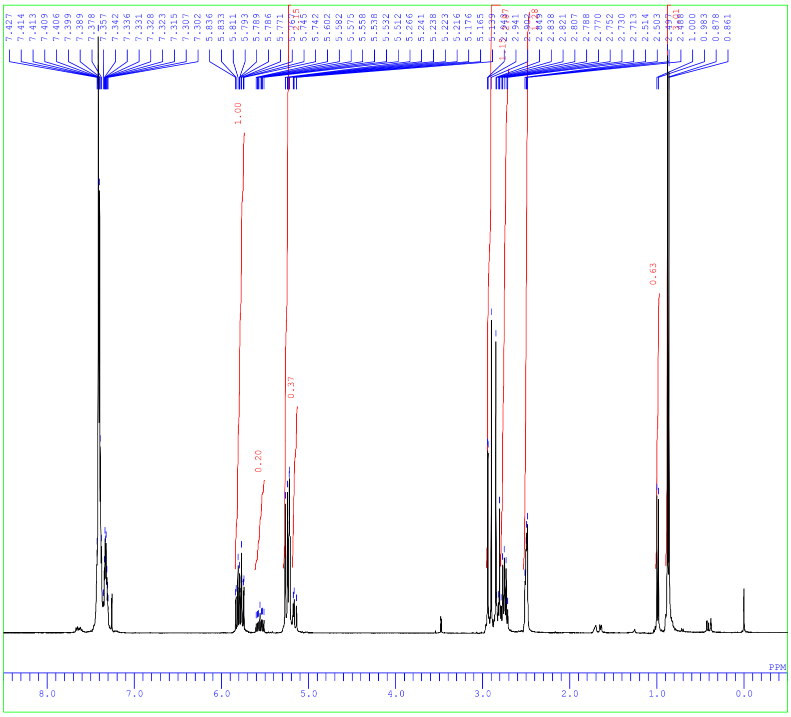

^{13}C NMR (major isomer)

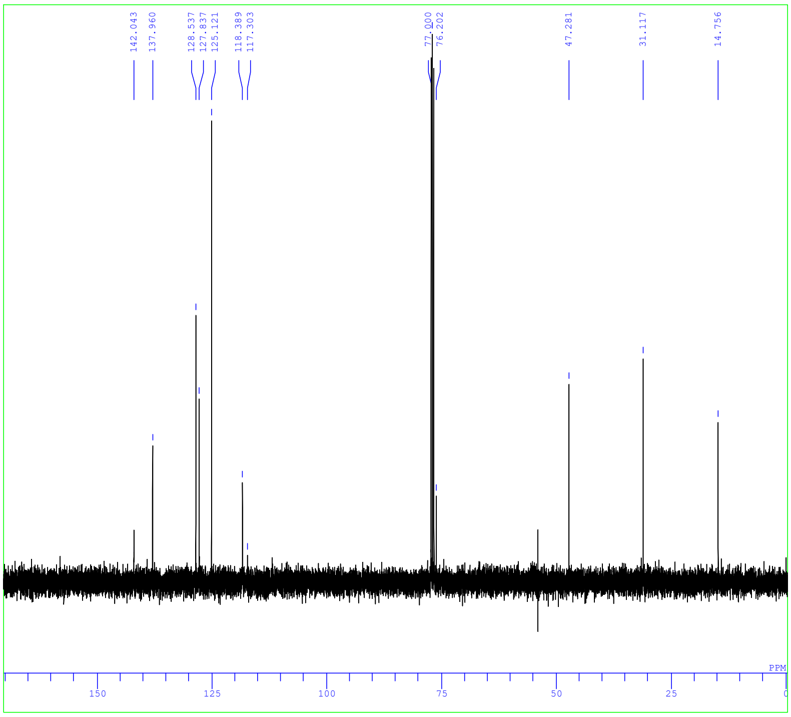

^{13}C NMR (major+minor isomer)

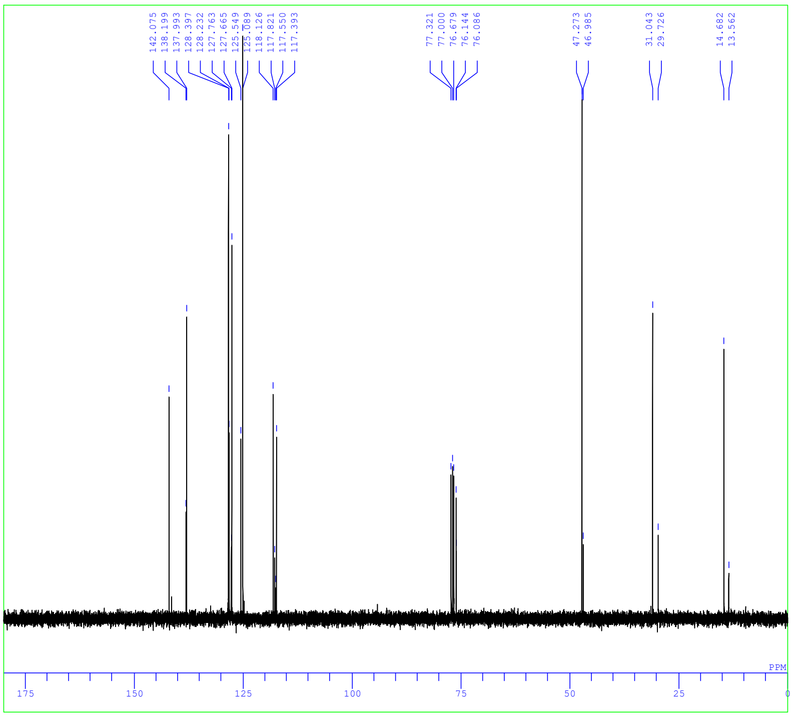

1-Bromo-3-methyl-2-phenyl-pen-4-tene-2-ol (3aj)

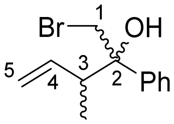

Isolate as a mixture of diastereomers

colorless liquid

IR (neat) 3548 cm^{-1} (OH)

MS (CI, 200eV) m/z 255 (M^++1, 4.30), bp: 110 °C /0.12 torr

HRMS calcd for $C_{12}H_{15}BrO$: 254.0306, found: m/z 255.0392 (CI, M^++1, +0.7 mmu)

^{1}H NMR ($CDCl_3$, 400 MHz) δ7.41-7.25 (m, 5H, Ph, major+minor), 5.91-5.82 (m, 1H, H_2C=C*H*, major), 5.65-5.56 (m, 1H, H_2C=C*H*, minor)*, 5.15-5.10 (m, 2H, *H*$_2$C=CH, major+minor), 3.99-3.85 (m, 2H, C*H*$_2$Br, minor)*, 3.92 (d, *J* =10.6 Hz, 1H, C*H*HBr, major), 3.86 (d, *J* = 10.6 Hz, 1H, CH*H*Br, major), 2.74 (m, 1H, CH_3C*H*, minor)*, 2.64 (m, 1H, CH_3C*H*, major), 2.55 (s, 1H, OH, minor)*, 2.50 (s, 1H, OH, major), 0.95 (d, *J* = 6.8 Hz, 3H, CH_3, minor)*, 0.89 (d, *J* = 7.0 Hz, 3H, CH_3, major)

^{13}C NMR ($CDCl_3$, 100 MHz) δ142.9, 139.1, 139.0*, 128.1, 127.8*, 127.3*, 127.2, 126.3*, 125.6, 116.7*, 116.6, 76.9, 47.6, 46.8*, 45.2, 44.5*, 15.9, 14.5*

* Mark corresponds to minor isomer.

^{1}H NMR (major isomer)

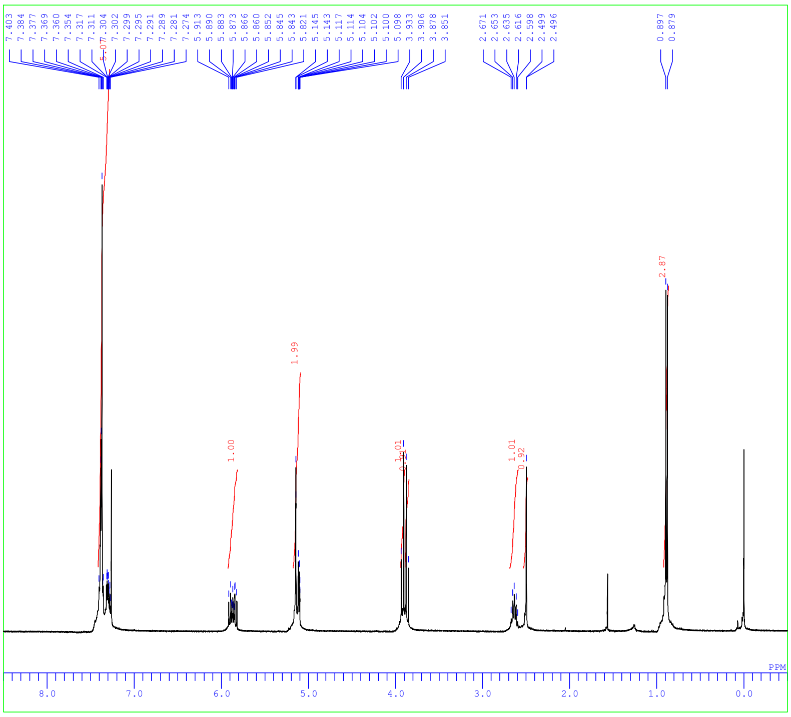

^{1}H NMR (major+minor isomer)

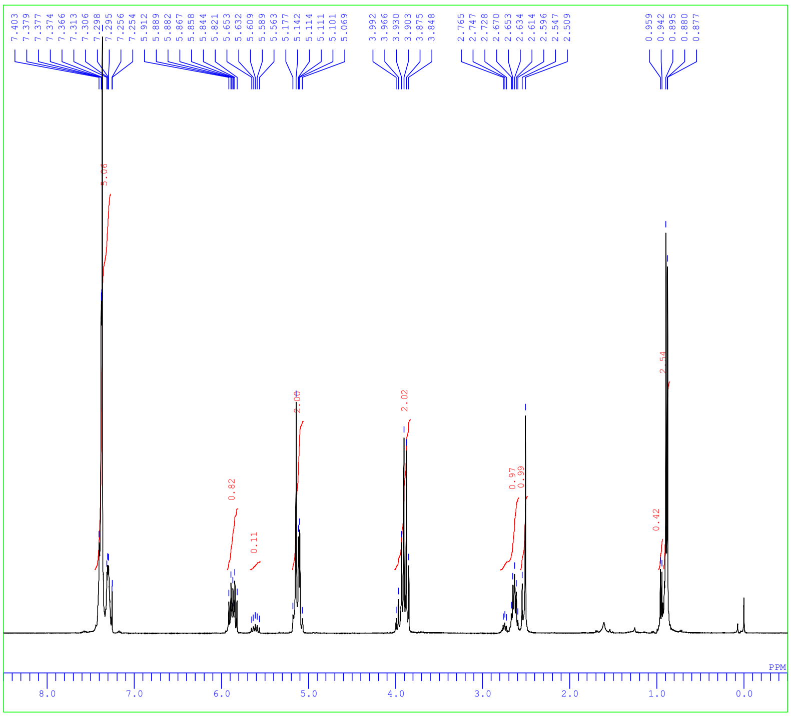

^{13}C NMR (major isomer)

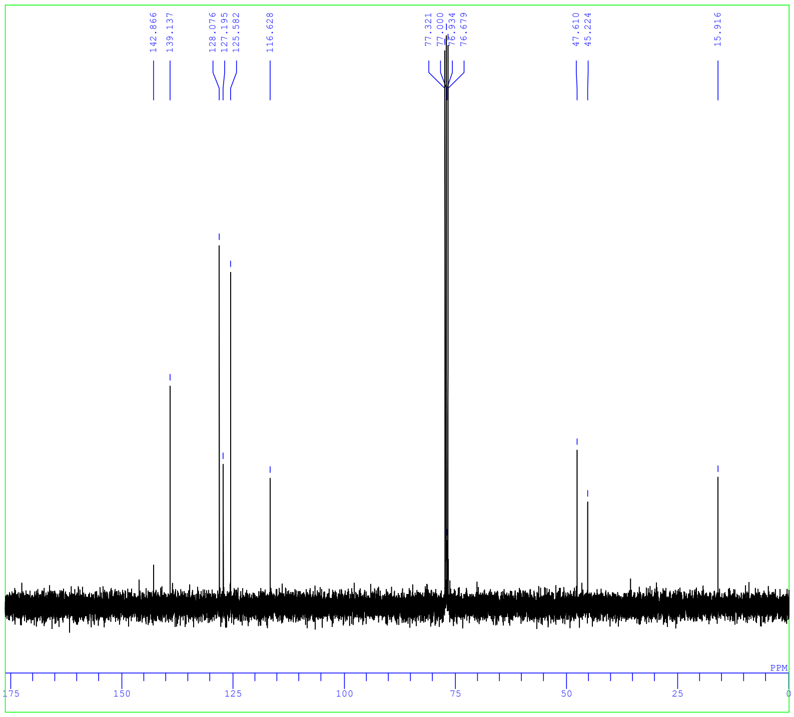

^{13}C NMR (major+minor isomer)

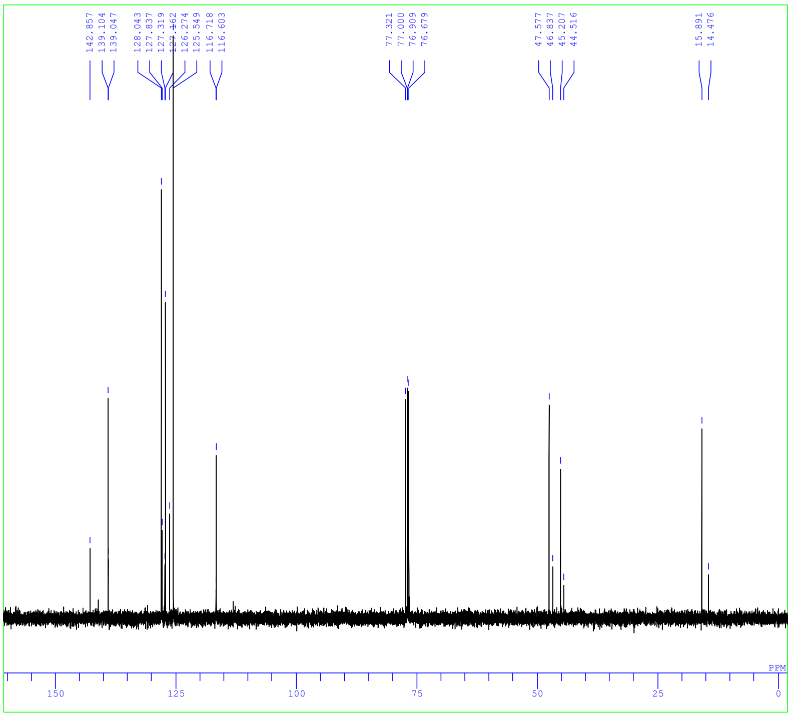

1-Methoxy-3-methyl-2-phenylpent-4-en-2-ol (3ak)

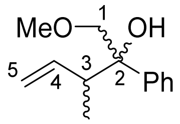

colorless liquid

IR (neat) 3555 cm^{-1} (OH), bp: 210 °C /1.0 torr

MS (CI, 200 eV) m/z 207 (M^{+}+1), 189 (100)

HRMS calcd for $C_{13}H_{18}O_2$: 206.1307, found: m/z 207.1386 (CI, M^{+}+1, +0.1 mmu)

^{1}H NMR ($CDCl_3$, 400 MHz) δ7.42 (m, 2H, Ph, major+minor), 7.35 (m, 2H, Ph, major+minor) 7.24 (m, 1H, Ph, major+minor), 5.94-5.86 (m, 1H, H_2C=C*H*, major), 5.67-5.58 (m, 1H, H_2C=C*H*, minor), 5.08-5.01 (m, 2H, *H_2*C=CH, major+minor), 3.76 (d, *J*= 9.2 Hz, 1H, C*H*HOMe, minor)*, 3.73 (d, *J* = 9.4 Hz, 1H, CH*H*OMe, major), 3.68 (d, *J* = 9.2 Hz, 1H, C*H*HOMe, minor)* 3.64 (d, *J*= 9.4 Hz, 1H, CH*H*OMe, major), 3.36 (s, 3H, OMe, minor)*, 3.30 (s, 3H, OMe, major), 2.86 (s, 1H, OH, minor)*, 2.78 (s, 1H, OH, major), 2.70-2.62 (m, 1H, CH_3C*H*, minor)*, 2.57-2.50 (m, 1H, CH_3C*H*, major), 0.92 (d, *J*=7.0 Hz, 3H, C*H_3*CH, minor)*, 0.82 (d, *J* =7.0 Hz, 3H, C*H_3*CH, major)

^{13}C NMR ($CDCl_3$, 100 MHz) δ143.8, 142.3*, 140.0, 139.8*, 127.9, 127.6*, 126.8*, 126.7, 126.1*, 125.6, 115.7*, 115.3, 78.6, 77.3, 77.0*, 59.3, 46.0, 45.9*, 14.8, 13.9* (Two peaks overlap)

^{1}H NMR (major isomer)

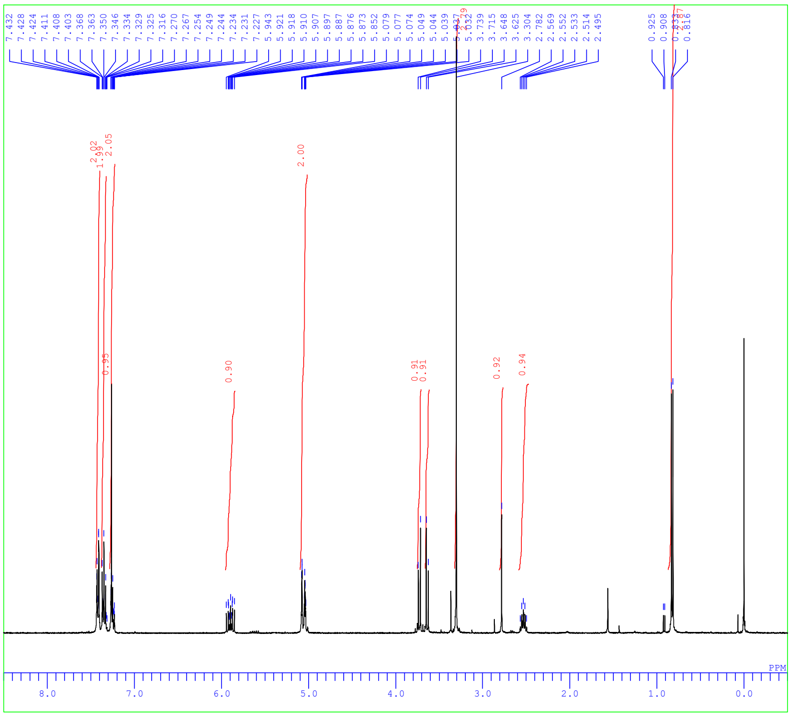

^{1}H NMR (major+minor isomer)

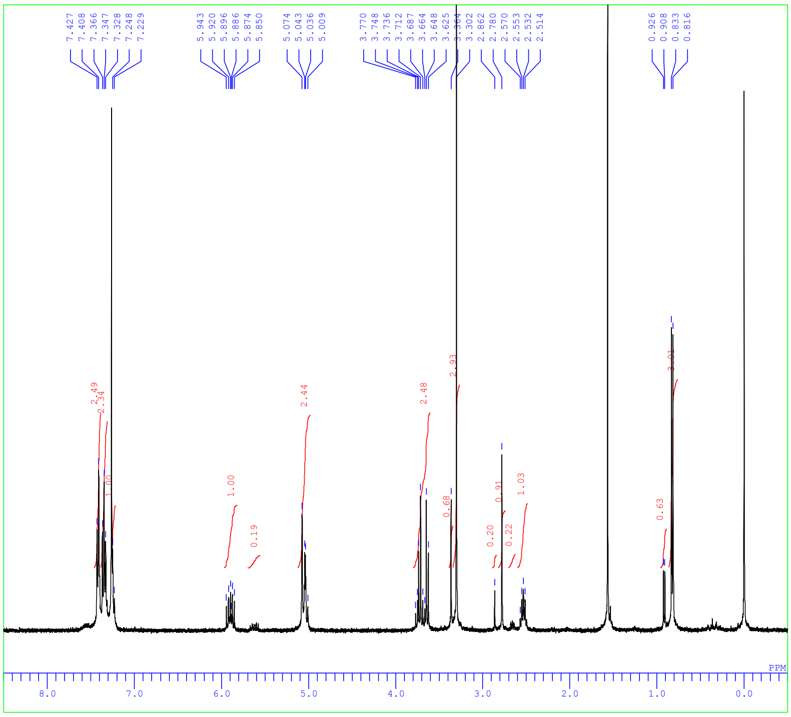

^{13}C NMR (major isomer)

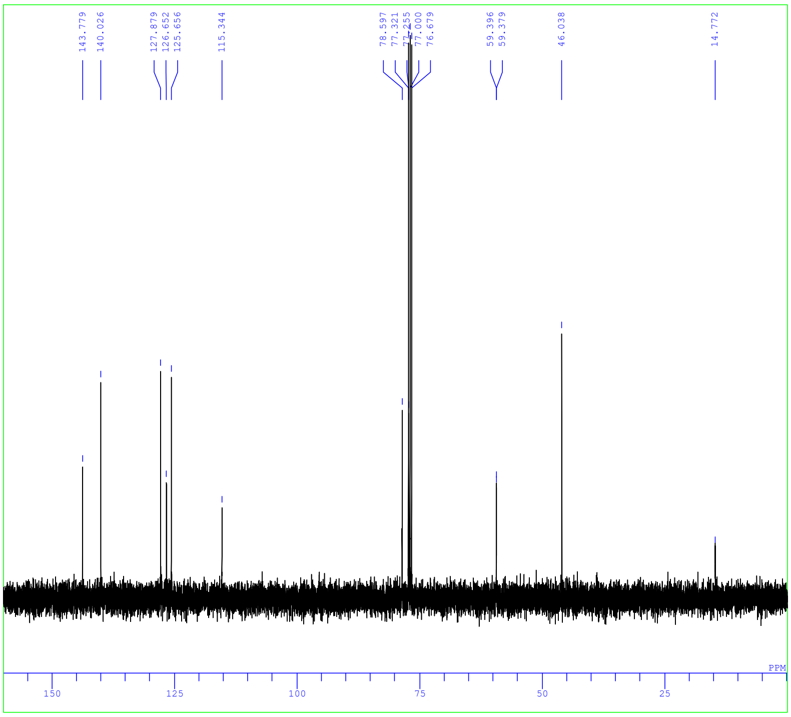

^{13}C NMR (major+minor isomer)

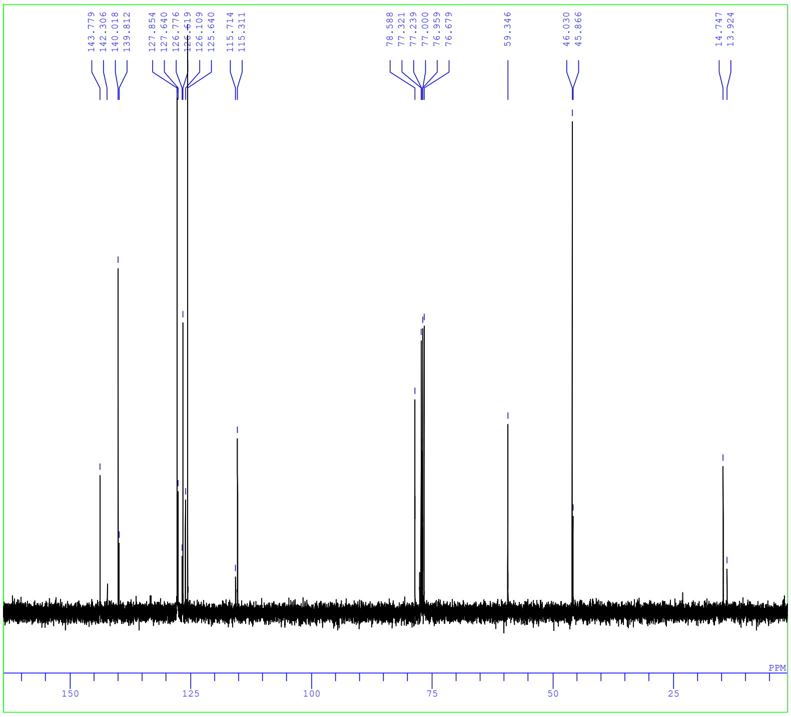

Ethyl 3-hydroxy-4-methyl-3-phenylhex-5-enoate (3al)

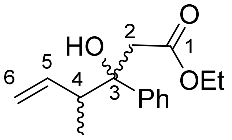

Major isomer

colorless liquid

IR (neat) 3487 cm^{-1} (OH), 1707 cm^{-1} (C=O) bp: 150 °C /2.5 torr

^{1}H NMR ($CDCl_3$, 400 MHz) δ7.41-7.38 (d, 2H, Ph), 7.34-7.30 (m, 2H, Ph), 7.24-7.20 (m, 1H, Ph), 5.96-5.87 (m, 1H, $H_2C{=}C\underline{H}$), 5.10-5.05 (m, 2H, $\underline{H}_2C{=}CH$), 4.41 (d, 1H, OH), 3.93 (m, 2H, OCH_2), 2.88 (s, 2H, $C\underline{H}_2C{=}O$), 2.44-2.36 (m, 1H, $CH_3C\underline{H}$), 1.00 (t, J = 7.1 Hz, 3H, $OCH_2C\underline{H}_3$), 0.82 (d, J = 6.8Hz, 3H, $C\underline{H}_3CH$)

^{13}C NMR ($CDCl_3$, 100 MHz) δ173.4, 144.7, 140.1, 127.9, 126.7, 125.5, 115.9, 76.5, 60.6, 49.4, 44.1, 14.7, 13.8

Anal. calcd for $C_{15}H_{20}O_3$: C, 72.55; H, 8.12, found: C, 72.35; H, 8.16

Minor isomer

colorless liquid

IR (neat) 3493 cm^{-1} (OH), 1710 cm^{-1} (C=O)

^{1}H NMR ($CDCl_3$, 400 MHz) δ7.39-7.36 (m, 2H, Ph), 7.33-7.29 (m, 2H, Ph), 7.25-7.21 (m, 1H, Ph), 5.70-5.61 (m, 1H, $H_2C{=}C\underline{H}$), 5.04-4.97 (m, 2H, $\underline{H}_2C{=}CH$), 4.46 (s, 1H, OH), 4.00 (q, J = 7.2 Hz, 2H, OCH_2), 3.12 (d, J = 15.9 Hz, 1H, $C\underline{H}HC{=}O$), 2.80 (d, J = 15.9 Hz, 2H, $CH\underline{H}C{=}O$), 2.57-2.50 (m, 1H, $CH_3C\underline{H}$), 1.08 (t, J = 7.1 Hz, 3H, $OCH_2C\underline{H}_3$), 0.92 (d, J = 6.8Hz, 3H, $C\underline{H}_3CH$)

^{13}C NMR ($CDCl_3$, 100 MHz) δ173.2, 143.6, 139.6, 127.7, 126.8, 126.0, 115.9, 76.5, 60.6, 48.6, 42.5, 14.0, 13.9

Anal. calcd for $C_{15}H_{20}O_3$: C, 72.55; H, 8.12, found: C, 72.52; H, 8.41

Properties for a mixture of diastereomer

HRMS calcd for $C_{15}H_{21}O_3$: 249.1491, found: m/z 249.1486 (CI, M^++1, -0.5 mmu)

MS (CI, 200 eV) m/z 105 (143), 193 (86), 147 (23)

^{1}H NMR (major isomer)

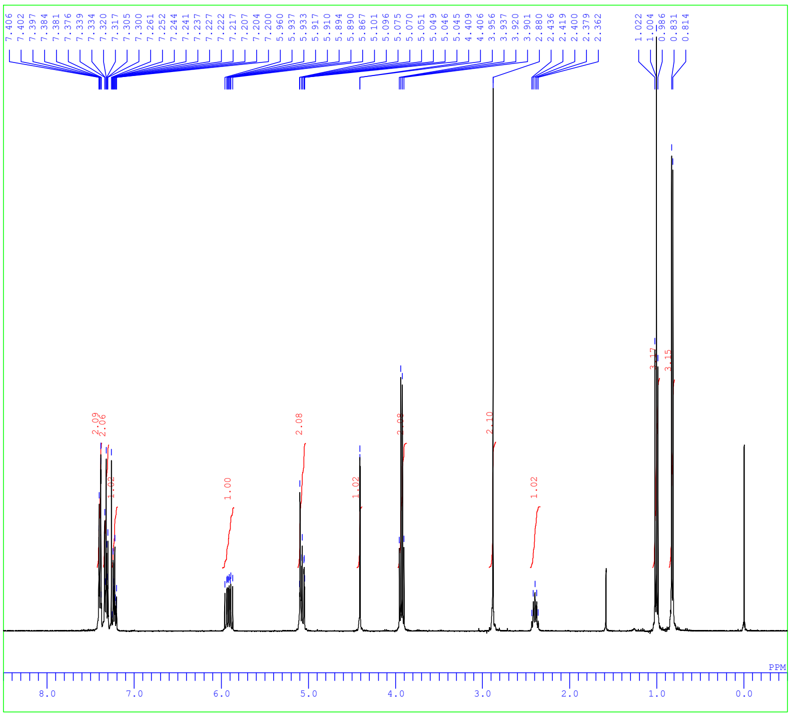

^{1}H NMR (minor isomer)

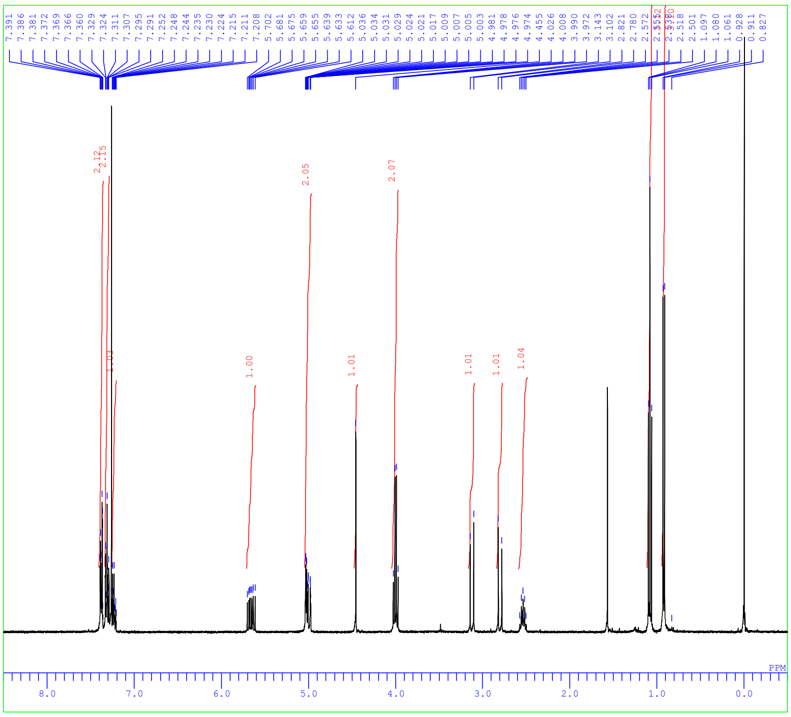

^{13}C NMR (major isomer)

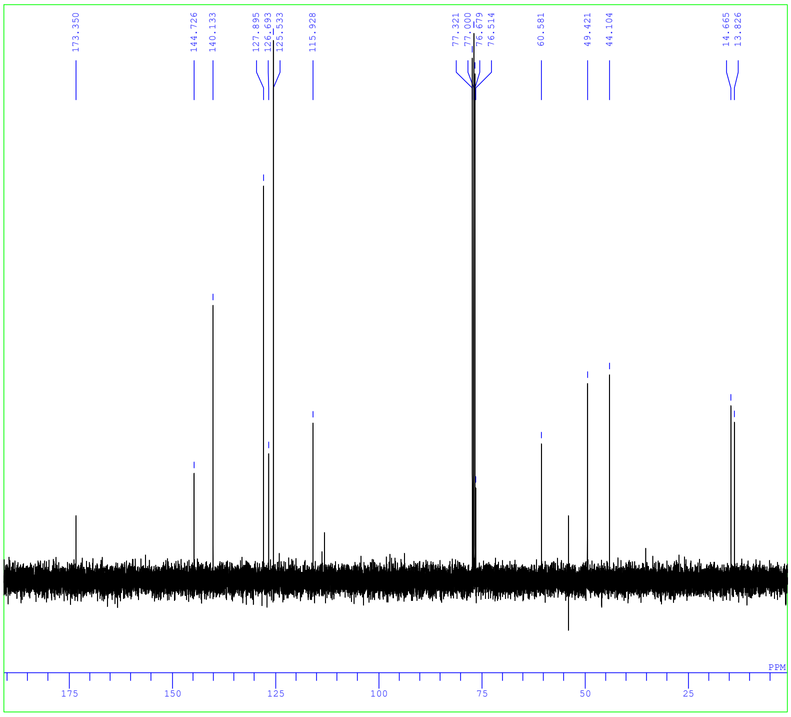

^{13}C NMR (minor isomer)

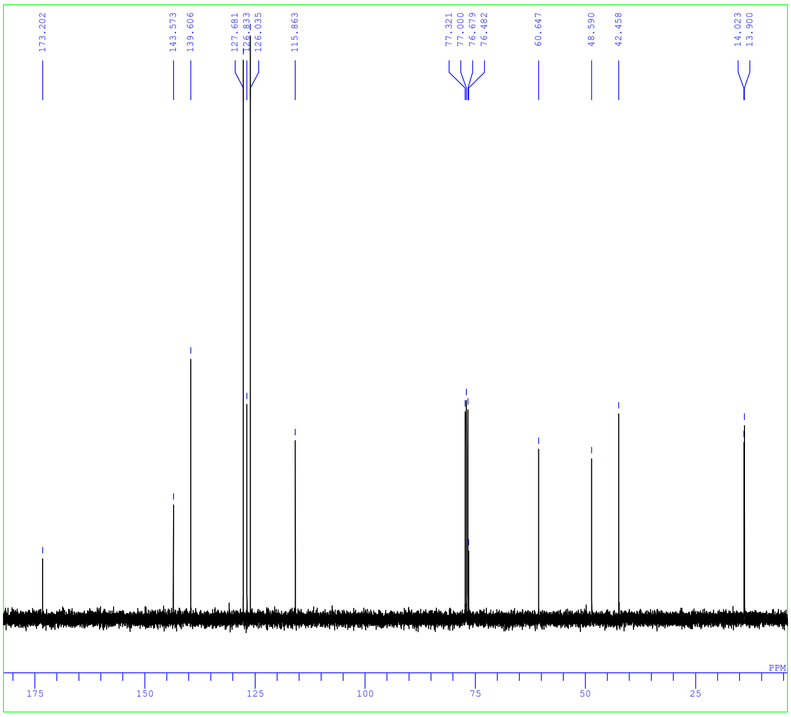

3,4-Dimethyl-1-phenylhex-5-en-3-ol (3am)[7]

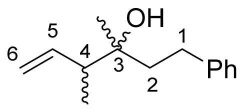

Major isomer

^{1}H NMR (CDCl$_3$, 400 MHz) : δ7.30–7.26 (m, 2H, Ph), 7.22–7.16 (m, 3H, Ph), 5.91-5.81 (m, 1H, H$_2$C=C*H*), 5.17-5.12 (m, 2H, *H*$_2$C=CH), 2.74-2.69 (m, 2H, C*H*$_2$CH$_2$Ph), 2.37-2.30 (m, 1H, CH$_3$C*H*), 1.81-1.76 (m, 2H, C*H*$_2$Ph), 1.55 (s, 1H, OH), 1.18 (s, 3H, C*H*$_3$COH), 1.05 (d, *J* =6.8 Hz, 3H, C*H*$_3$CH),

^{13}C NMR (CDCl$_3$,100 Hz): δ142.7, 140.1, 128.3, 125.6, 116.6, 73.5, 47.4, 42.0, 29.8, 23.5, 14.9

MS (CI, 200 eV) m/z 187 (225), 131 (86), 188 (35), 117 (33)

HRMS calcd for [$C_{14}H_{20}O$-OH]$^+$: 187.1481, found: m/z 187.1484 (CI)

Minor isomer

^{1}H NMR (CDCl$_3$, 400 MHz) : δ7.30–7.26 (m, 2H, Ph), 7.20–7.16 (m, 3H, Ph), 5.84-5.75 (m, 1H, H$_2$C=C*H*), 5.14-5.01 (m, 2H, *H*$_2$C=CH), 2.80-2.61 (m, 2H, C*H*$_2$CH$_2$Ph), 2.36-2.28 (m, 1H, CH$_3$C*H*), 1.82-1.66 (m, 2H, C*H*$_2$Ph), 1.49 (s, 1H, OH), 1.21 (s, 3H, C*H*$_3$CH), 1.06 (d, *J* =7.0 Hz, 3H, C*H*$_3$CH),

^{13}C NMR (CDCl$_3$,100 Hz): δ142.8, 140.2, 128.4, 128.3, 125.7, 116.3, 73.7, 47.5, 42.2, 29.8, 23.8, 14.6

^{1}H NMR (major isomer)

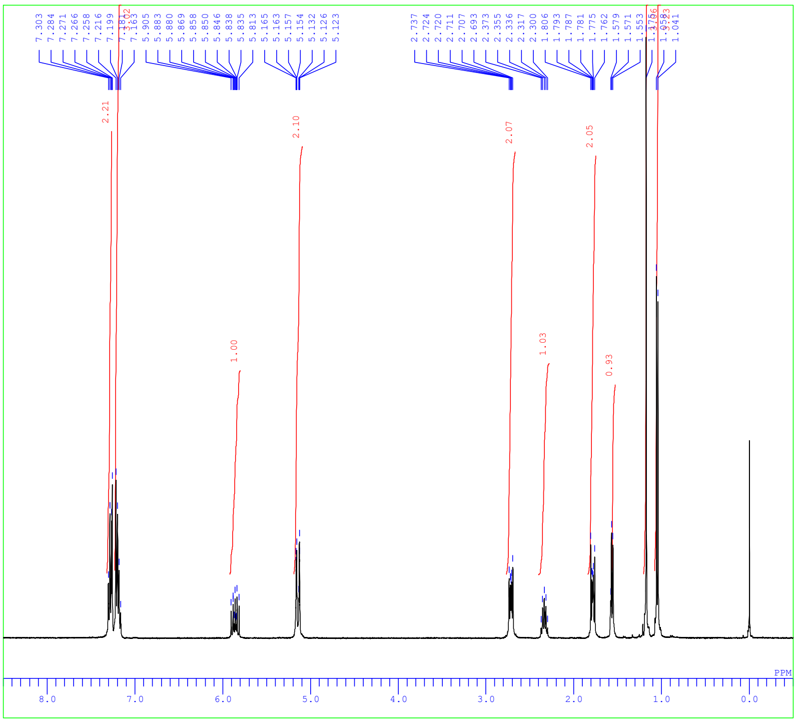

^{1}H NMR (minor isomer)

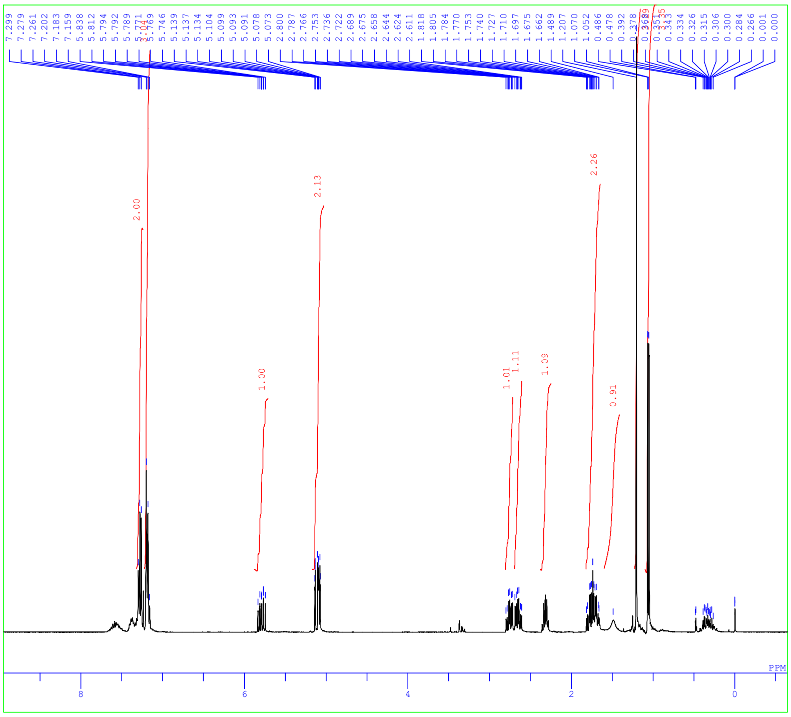

^{13}C NMR (major isomer)

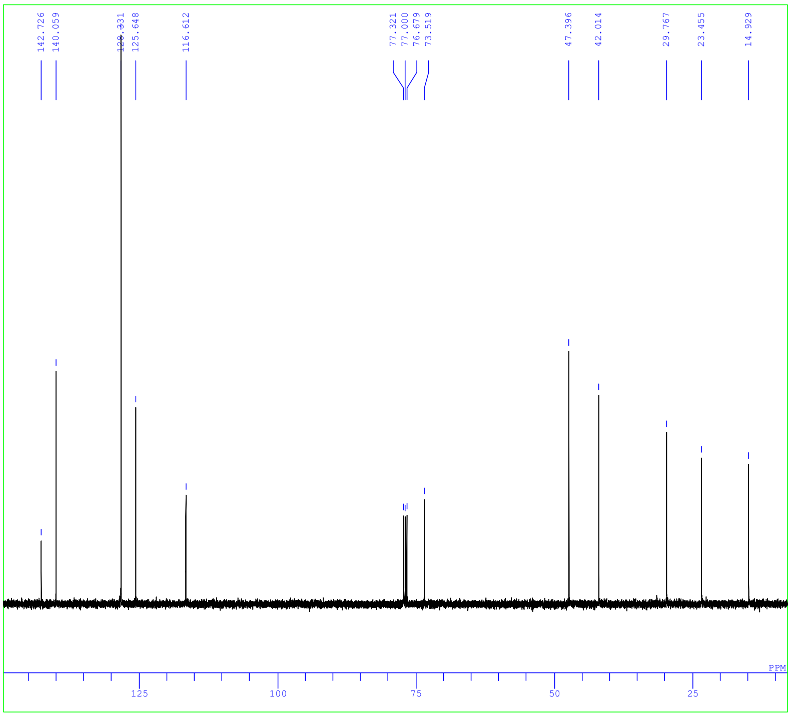

^{13}C NMR (minor isomer)

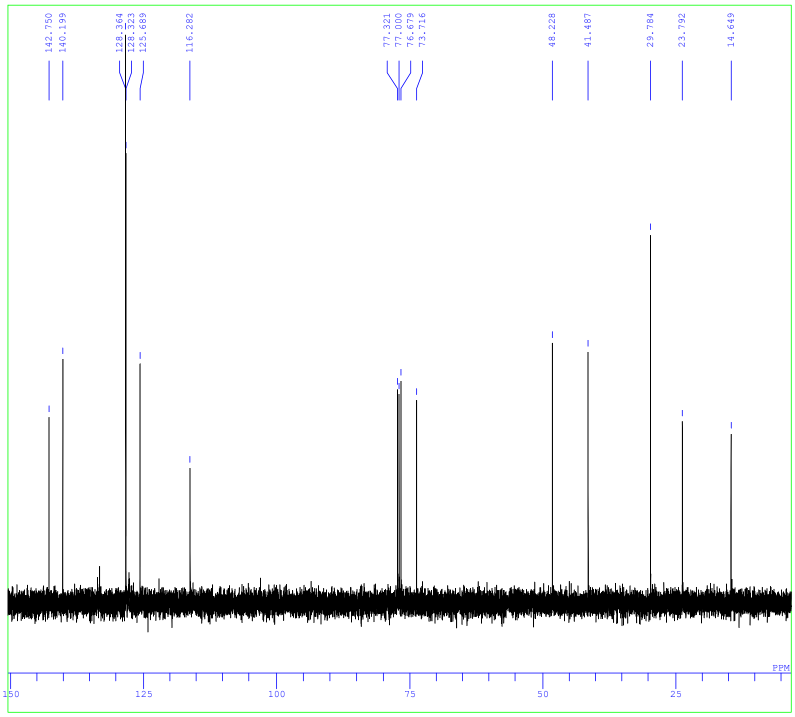

1-(But-3-en-2-yl)cyclohexan-1-ol (3an)[3]

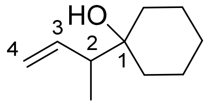

^{1}H NMR ($CDCl_3$, 400 MHz) δ5.88-5.79 (m, 1H, $H_2C{=}C\underline{\textit{H}}$), 5.11-5.04 (m, 2H, $\underline{\textit{H}_2}C{=}CH$), 2.21-2.14 (m, 1H, $CH_3C\underline{\textit{H}}$), 1.61-1.35 (m, 10H, Cy), 1.22-1.12 (m, 1H, Cy or OH), 1.02 (d, *J* = 6.8 Hz, 3H, $C\underline{\textit{H}_3}CH$)

^{13}C NMR ($CDCl_3$, 100 MHz) δ140.4, 115.8, 72.4, 48.3, 34.9, 34.3, 25.8, 21.8, 14.1

MS (CI, 200 eV) m/z 137 (297), 138 (38), 99 (21)

HRMS calcd for $[C_{10}H_{18}O\text{-}OH]^+$: 137.1325, found: m/z 137.1332 (CI+)

^{1}H NMR

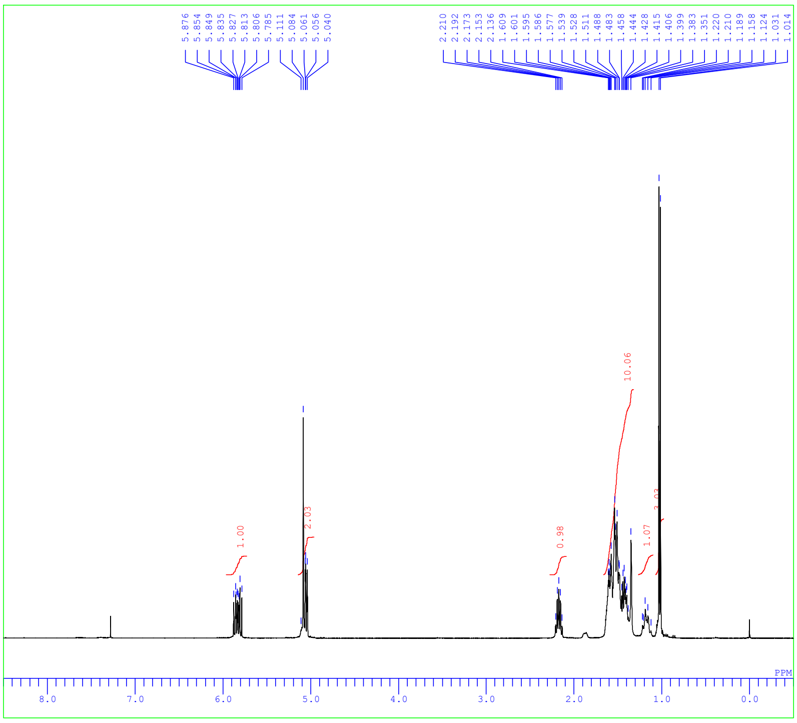

^{13}C NMR

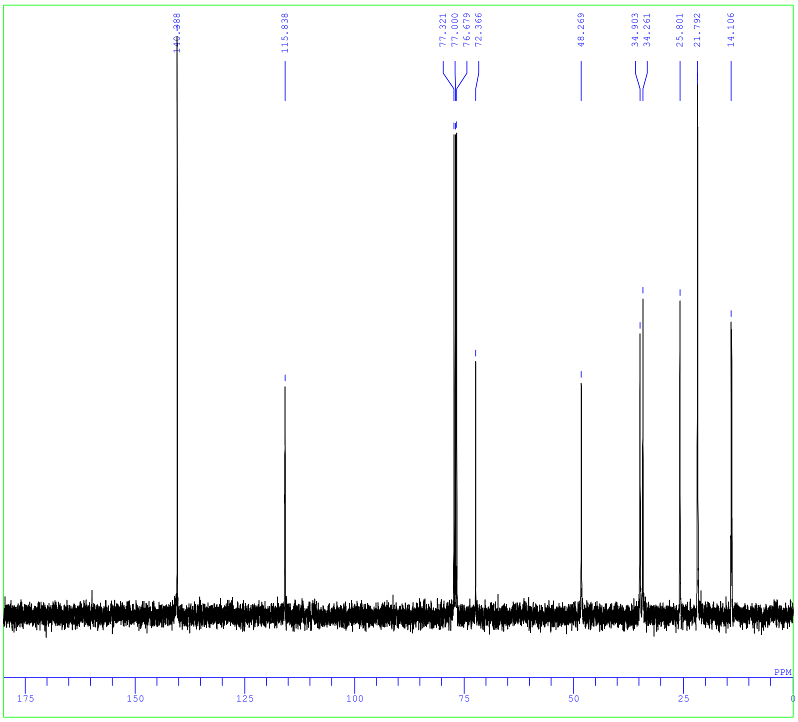

140.388
115.838
77.321
77.000
76.679
72.366
48.269
34.903
34.261
25.801
21.792
14.106
PPM
175
150
125
100
75
50
25
0

3-Methyl-2-(1-naphthyl)-4-pentene-2-ol (3ao)

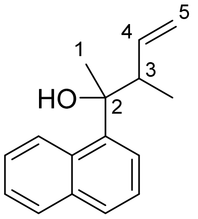

Isolated as mixture of diastereomers

^{1}H NMR ($CDCl_3$, 400 MHz) δ8.86 (m, 1H, Ar, major,), 8.64 (m, 1H, Ar, minor,)*, 7.88-7.39 (m, major+minor, 6H, Ar), 5.97-5.89 (m, major, 1H, H_2C=C*H*), 5.87-5.78 (m, minor, 1H, H_2C=C*H*)*, 5.28-5.21 (m, major, 2H, *H*$_2$C=CH), 5.04-4.97 (m, minor, 2H, *H*$_2$C=CH)*, 3.44-3.30 (m, major+minor, 1H, CH_3C*H*), 2.29 (s, major, 1H, OH), 2.28 (s, minor, 1H, OH)*, 1.77 (s, 3H, minor, C*H*$_3$COH)*, 1.74 (s, major, 3H, C*H*$_3$COH), 0.99 (d, J = 6.8 Hz, minor, 3H, C*H*$_3$CH)*, 0.85 (d, J = 6.8 Hz, major, 3H, C*H*$_3$CH)

^{13}C NMR ($CDCl_3$, 100 MHz) δ142.4*, 141.7, 140.0*, 139.7, 134.9, 134.8*, 130.7, 130.5*, 129.1 (major+minor), 128.5, 128.3*, 127.1, 126.5*, 125.1 (major+minor), 125.0, 124.9*, 124.7 (major+minor), 124.4, 124.3*, 116.8, 115.5*, 78.0, 77.5*, 46.0*, 45.9, 27.1*, 25.0, 14.9, 14.0* 140.0, 127.8, 127.7*, 126.2*, 126.1, 125.8*, 125.6, 116.2 (major+minor), 78.1, 77.9*, 48.5, 47.7*, 43.2, 41.2*, 16.7 (major+minor), 14.8, 14.4 (major+minor), 13.2*

* Mark corresponds to minor isomer.

MS (CI, 200 eV) m/z 171 (180), 172 (23)

HRMS calcd for $[C_{16}H_{18}O+1]^+$: 227.1436, found: m/z 224.1437 (CI)

^{1}H NMR (major isomer)

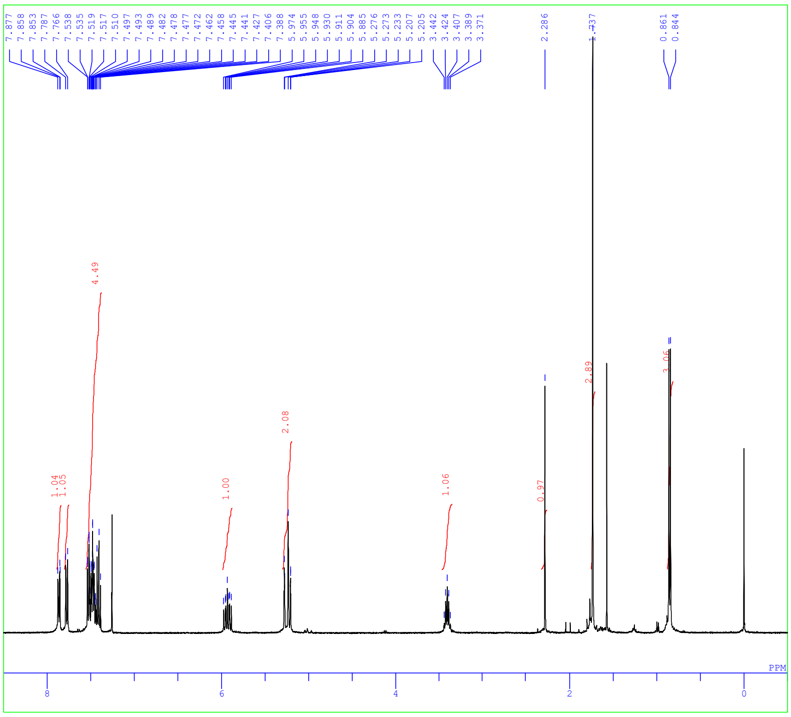

^{13}C NMR (major isomer)

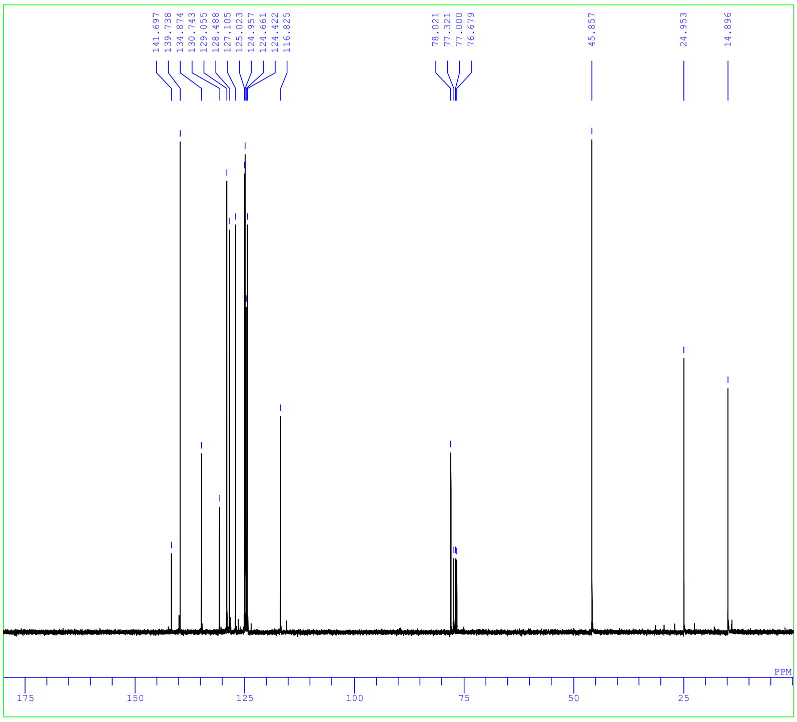

^{1}H NMR (major+minor isomer)

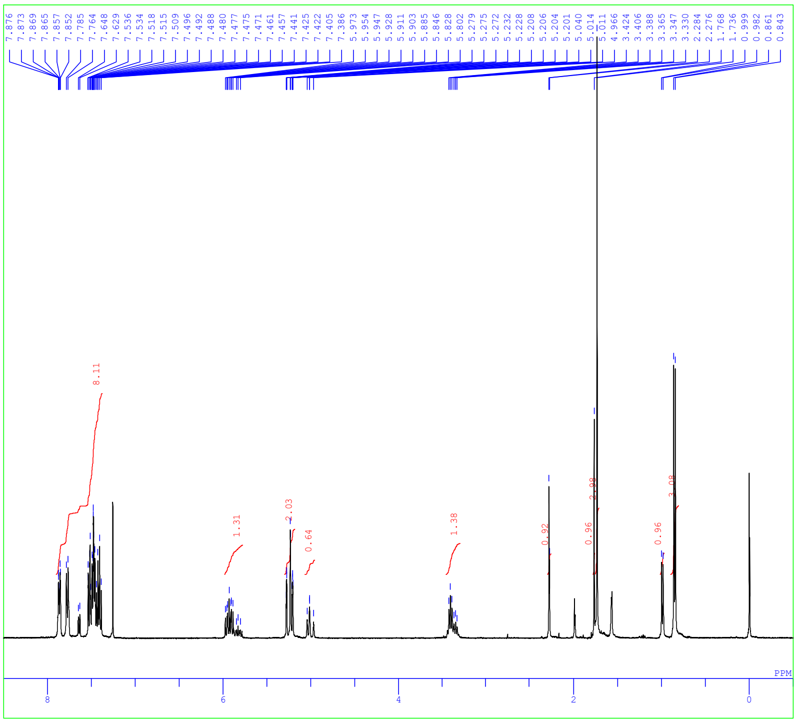

^{13}C NMR (major+minor isomer)

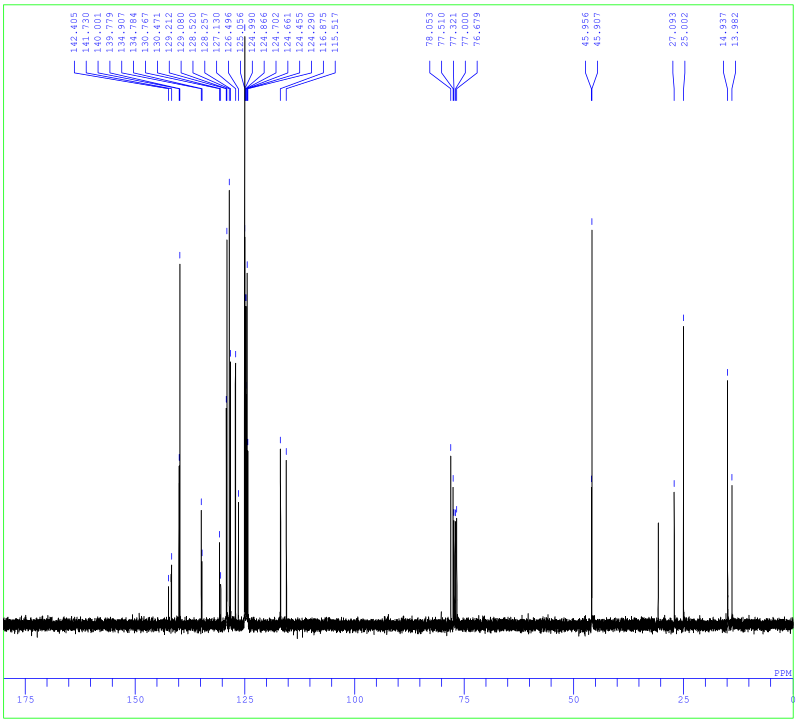

3-Methyl-2-(naphthalen-1-yl)pent-4-en-2-ol (3ap)

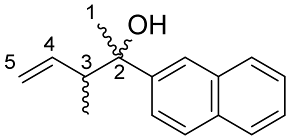

Major isomer

^{1}H NMR ($CDCl_3$, 400 MHz) : δ7.89–7.79 (m, 4H, Ar), 7.50-7.43 (m, 3H, Ar), 5.91-5.82 (m, 1H, H_2C=C*H*), 5.16-5.10 (m, 2H, *H*$_2$C=CH), 2.70-2.62 (m, 1H, CH_3C*H*), 2.03 (br, 1H, OH), 1.60 (s, 3H, C*H*$_3$COH), 0.87 (d, *J* = 7.0 Hz, 3H, C*H*$_3$CH)

^{13}C NMR ($CDCl_3$, 100 Hz):δ144.4, 139.7, 133.0, 132.1, 128.1, 127.5, 127.4, 125.9, 125.6, 123.8, 123.7, 116.4, 75.9, 48.6, 28.6, 14.8

MS (CI, 200 eV) m/z 209 (299), 210 (49), 171 (29)

HRMS calcd for $[C_{16}H_{18}O\text{-}OH]^+$: 209.1328, found: m/z 209.1330 (CI)

Minor isomer

^{1}H NMR ($CDCl_3$, 400 MHz) : δ7.87–7.80 (m, 4H, Ar), 7.57-7.42 (m, 3H, Ar), 5.77-5.68 (m, 1H, H_2C=C*H*), 5.15-5.07 (m, 2H, *H*$_2$C=CH), 2.76-2.68 (m, 1H, CH_3C*H*), 2.12 (br, 1H, OH), 1.61 (s, 3H, C*H*$_3$COH), 1.00 (d, *J* = 7.0 Hz, 3H, C*H*$_3$CH)

^{13}C NMR ($CDCl_3$, 100 Hz): δ144.6, 139.8, 133.0, 132.2, 128.1, 127.5, 127.4, 125.9, 125.6, 124.1, 124.0, 116.7, 75.8, 48.4, 25.9, 14.0

MS (CI, 200 eV) m/z 209 (1083), 210 (203), 171 (65), 365 (57), 211 (21)

HRMS calcd for $[C_{16}H_{18}O\text{-}OH]^+$: 209.1325, found: m/z 209.1330 (CI)

^{1}H NMR (major isomer)

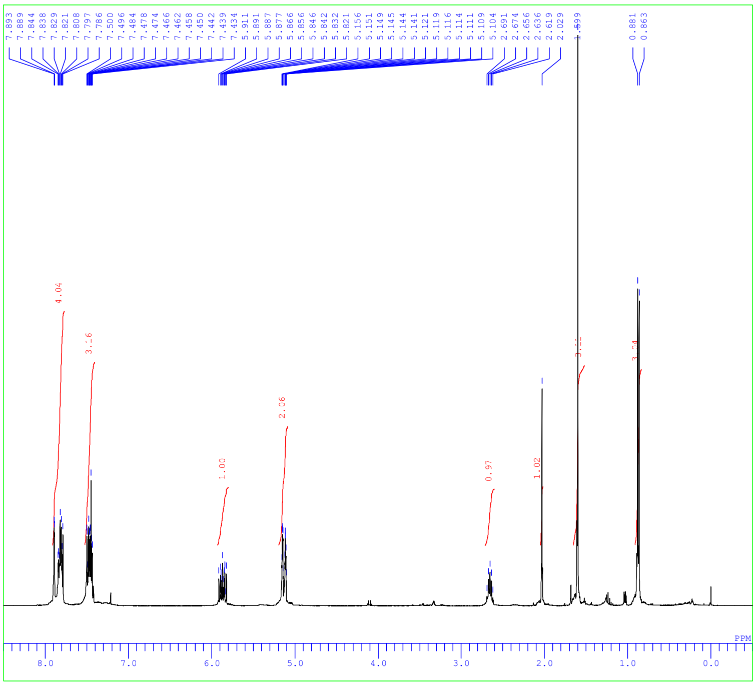

^{13}C NMR (major isomer)

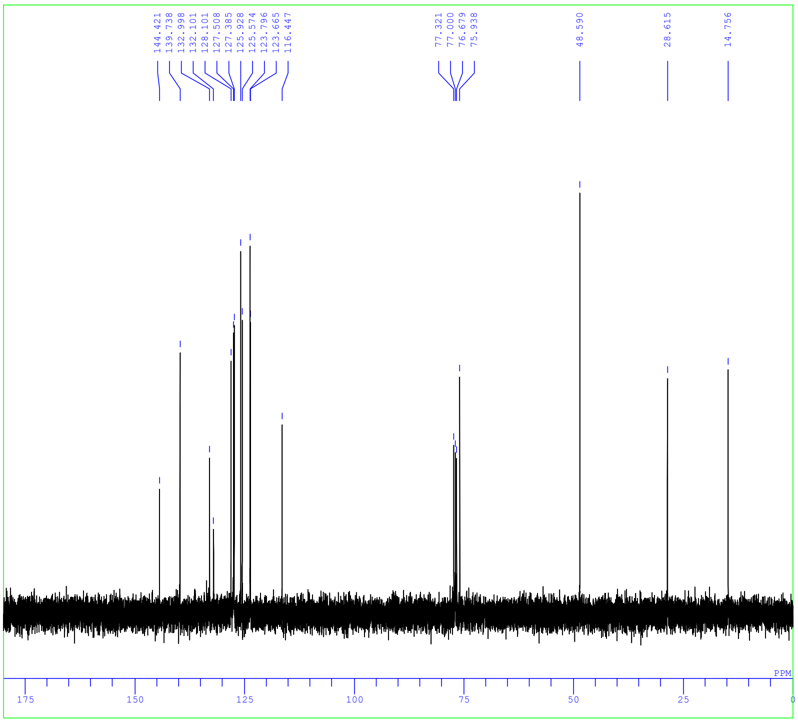

^{1}H NMR (minor isomer)

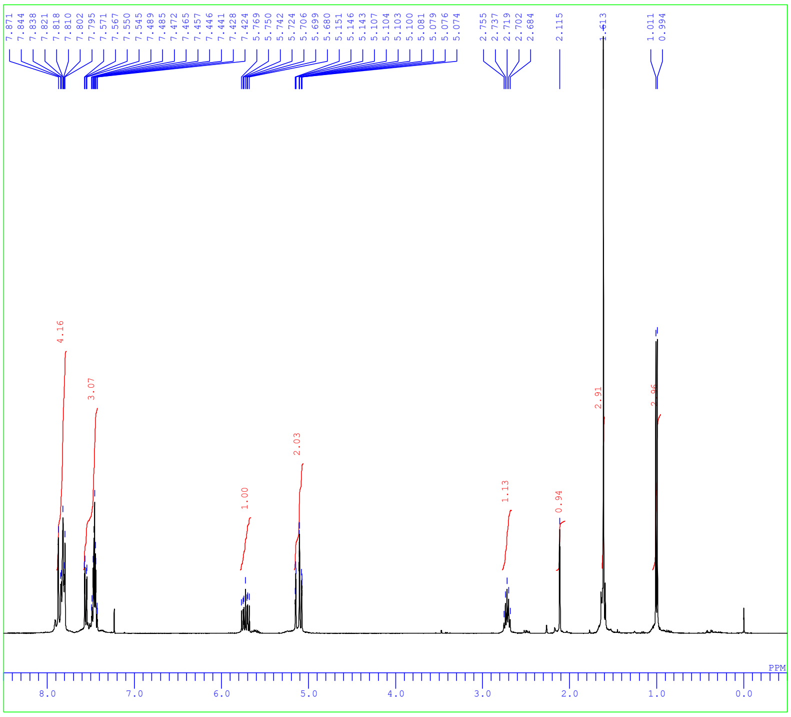

^{13}C NMR (minor isomer)

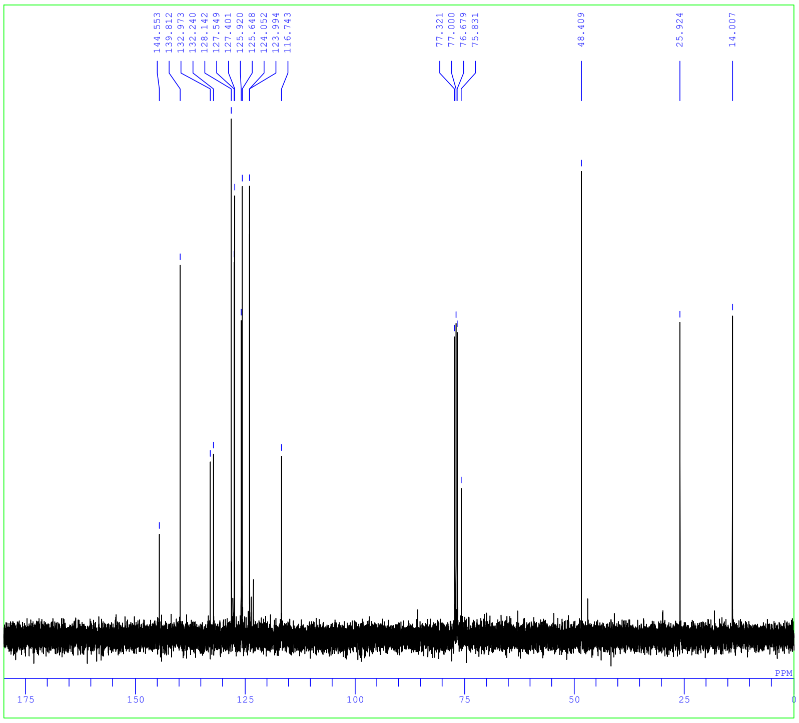

3,4-Dimethyl-2-phenylpent-4-en-2-ol (3ba)

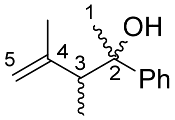

Major isomer

^{1}H-NMR ($CDCl_3$, 400 MHz) δ: 7.44-7.21 (m, 5H, Ph), 4.95 (s, 1H, <u>*H*</u>HC=CCH_3), 4.87 (s, 1H, H<u>*H*</u>C=CCH_3), 2.57 (q, *J* = 7.2 Hz, 1H, CH_3C<u>*H*</u>), 2.00 (s, 1H, OH), 1.72 (s, 3H, H_2C=CC<u>*H_3*</u>), 1.51 (s, 3H, C<u>*H_3*</u>COH), 0.88 (d, *J* = 7.2 Hz, 3H, C<u>*H_3*</u>CH)

^{13}C NMR ($CDCl_3$,100 Hz): δ147.9, 147.5, 127.8, 126.2, 125.0, 113.3, 75.7, 51.4, 29.4, 22.5, 14.4

MS (CI, 200 eV) m/z 173 (178), 121 (31), 174 (22)

HRMS calcd for $[C_{13}H_{18}O\text{-}OH]^+$: 173.1325, found: m/z 173.1332 (CI)

Minor isomer

^{1}H-NMR ($CDCl_3$, 400 MHz) δ: 7.41-7.12 (m, 5H, Ph), 4.87 (s, 1H, <u>*H*</u>HC=CCH_3), 4.77 (s, 1H, H<u>*H*</u>C=CCH_3), 2.61 (q, *J* = 7.1 Hz, 1H, CH_3C<u>*H*</u>), 2.31 (s, 1H, OH), 1.54 (s, 3H, H_2C=CC<u>*H_3*</u>), 1.39 (s, 3H, C<u>*H_3*</u>COH), 1.09 (d, *J* = 7.2 Hz, 3H, C<u>*H_3*</u>CH)

^{13}C NMR ($CDCl_3$,100 Hz): δ148.3, 148.0, 127.8, 126.4, 125.2, 113.3, 75.1, 51.3, 26.7, 23.5, 14.9

MS (CI, 200 eV) m/z 173 (689), 121 (138), 174 (104)

HRMS calcd for $[C_{13}H_{18}O\text{-}OH]^+$: 173.1325, found: m/z 173.1329 (CI)

^{1}H NMR (major isomer)

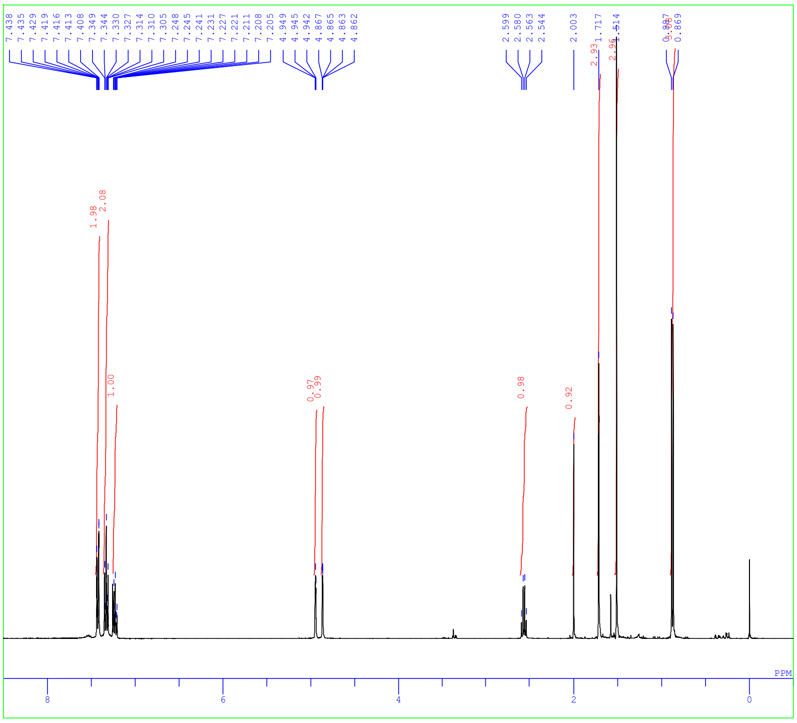

^{13}C NMR (major isomer)

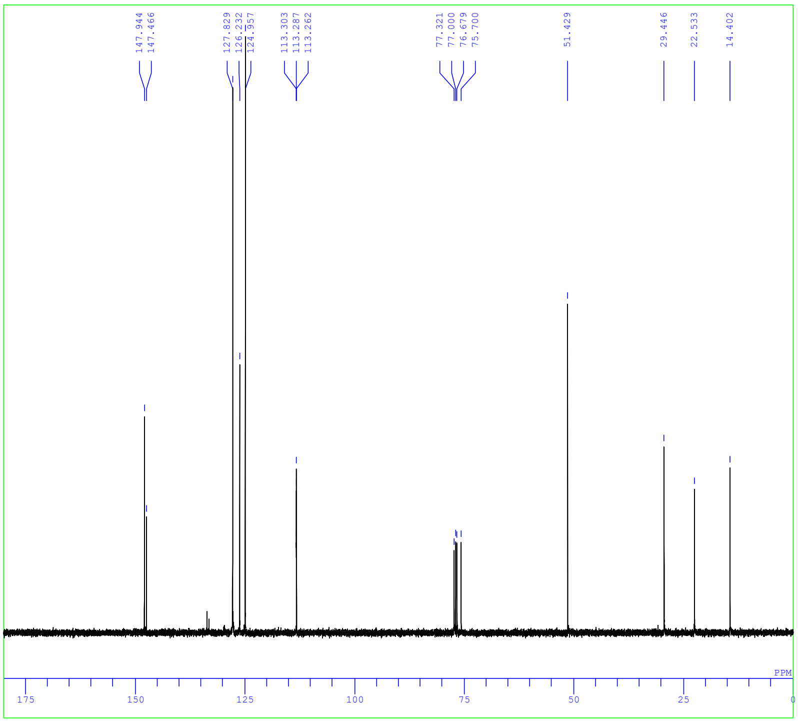

^{1}H NMR (minor isomer)

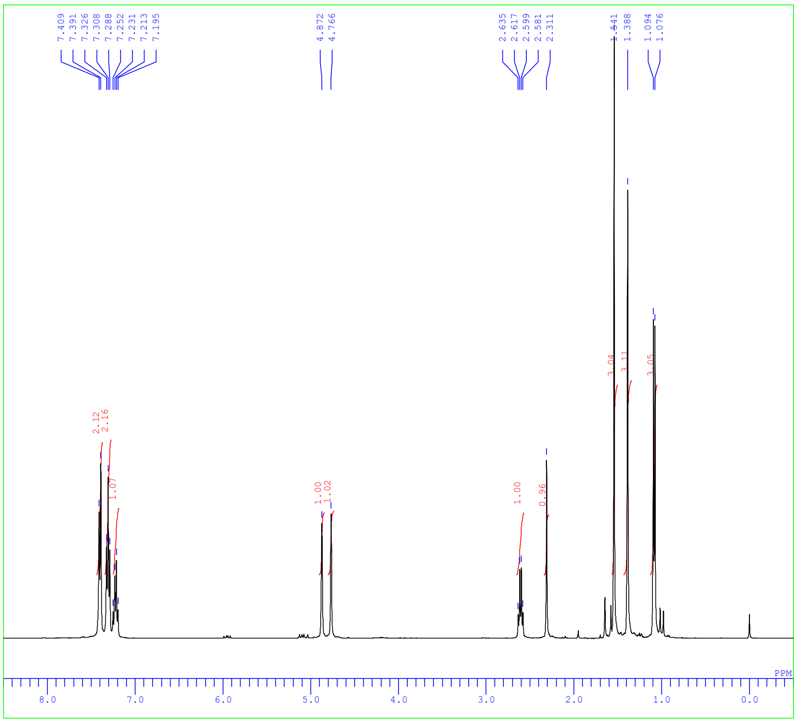

^{13}C NMR (minor isomer)

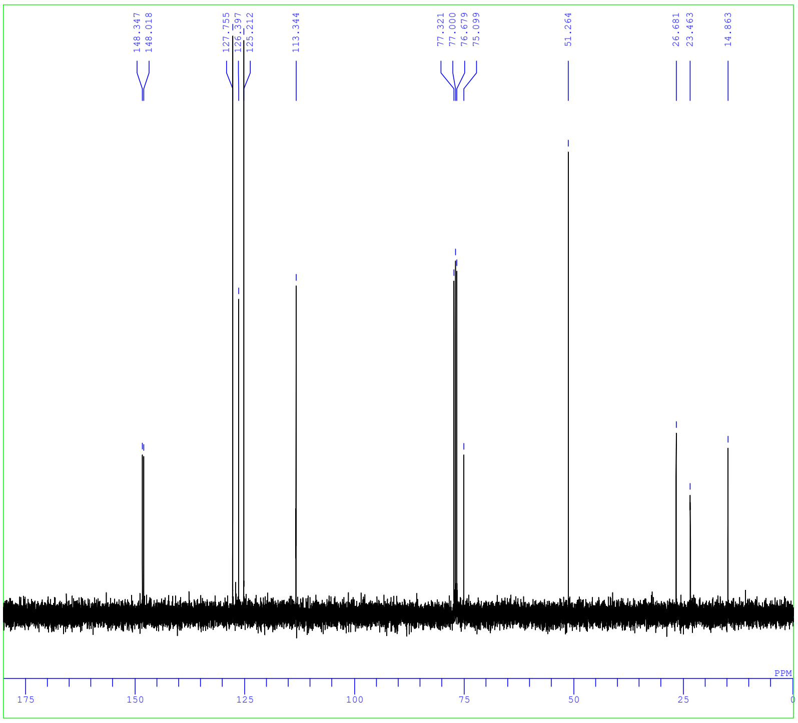

3,3-Dimethyl-2-phenylpent-4-en-2-ol (3ba')

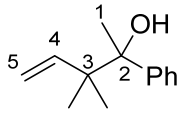

^{1}H-NMR ($CDCl_3$, 400 MHz) δ: 7.42-7.21 (m, 5H, Ph), 5.95 (dd, *J* = 17.5, 10.7 Hz, 1H, $H_2C{=}C\underline{\mathit{H}}$), 5.13-5.03 (m, 2H, $\underline{\mathit{H}}_2C{=}CH$), 1.94 (s, 1H, OH), 1.58 (s, 3H, $C\underline{\mathit{H}}_3COH$), 1.02 (s, 3H, $C\underline{\mathit{H}}_3CCH_3$), 0.98 (s, 3H, $CH_3CC\underline{\mathit{H}}_3$)

^{13}C NMR ($CDCl_3$, 100 MHz) δ 145.3, 145.1, 127.1, 127.0, 126.4, 113.8, 77.5, 44.3, 25.3, 22.7, 22.4

MS (CI, 200 eV) m/z 173 (1093), 174 (151), 121 (77)

HRMS calcd for $[C_{13}H_{18}O\text{-}OH]^{+}$: 173.1325, found: m/z 173.1331 (CI)

^{1}H NMR

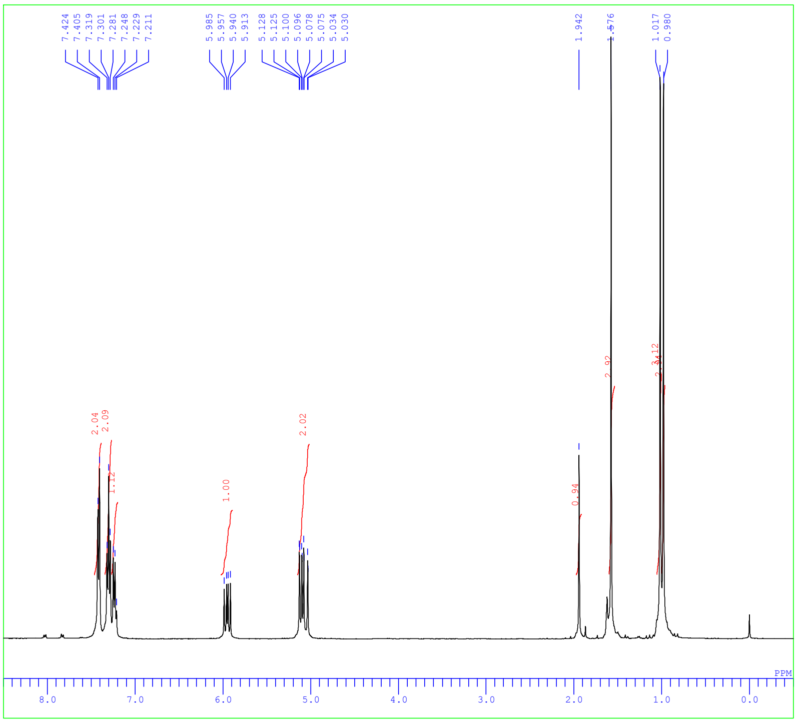

^{13}C NMR

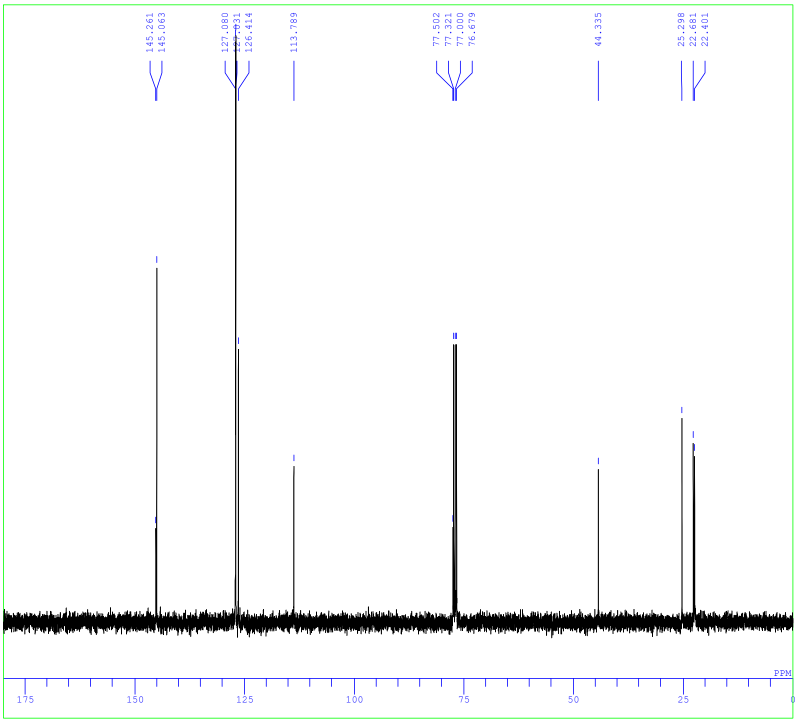
145.261
145.063
127.080
127.031
126.414
113.789
77.502
77.321
77.000
76.679
44.335
25.298
22.681
22.401
PPM
175
150
125
100
75
50
25
0

3,3,4-Trimethyl-2-phenylpent-4-en-2-ol (3ca)[8]

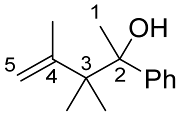

^{1}H NMR (CDCl$_3$, 400 MHz) δ7.43-7.40 (m, 2H, Ph), 7.31-7.20 (m, 3H, Ph), 5.05 (s, <u>*H*</u>HC=C), 4.91 (s, 1H, H<u>*H*</u>C=C), 2.44 (s, 1H, OH), 1.59 (s, 3H, H$_2$C=CC<u>*H*</u>$_3$ or C<u>*H*</u>$_3$COH), 1.57 (s, 3H, H$_2$C=CC<u>*H*</u>$_3$ or C<u>*H*</u>$_3$COH), 1.12 (s, 3H, C<u>*H*</u>$_3$CCH$_3$), 1.06 (s, 3H, CH$_3$CC<u>*H*</u>$_3$)

^{13}C NMR (CDCl$_3$,100 Hz): δ151.4, 145.6, 127.0, 126.9, 126.3, 114.1, 77.0, 46.5, 25.4, 24.3, 23.8, 23.3

MS (CI, 200 eV) m/z 187 (124), 121 (26)

^{1}H NMR

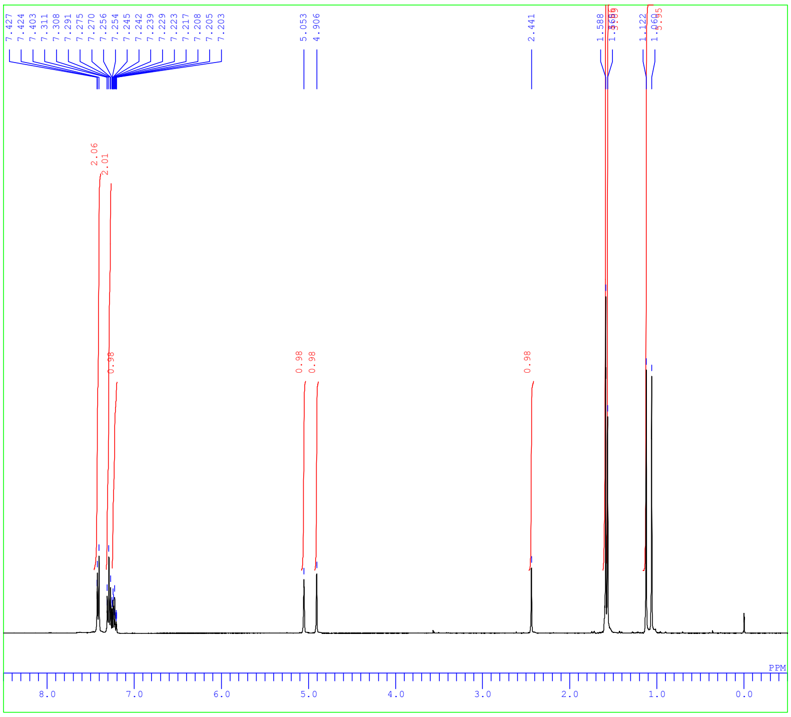

^{13}C NMR

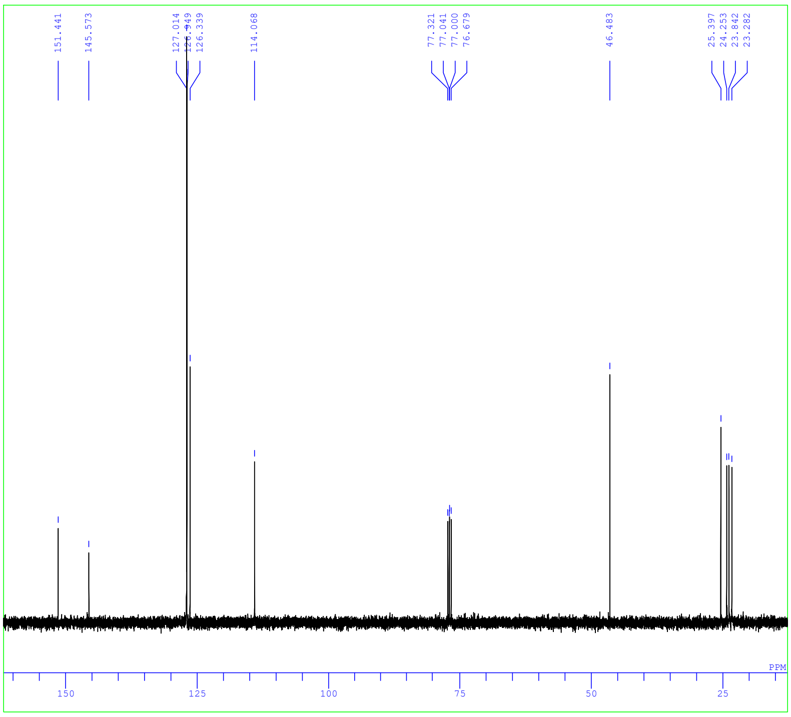
151.441
145.573
127.014
126.949
126.339
114.068
77.321
77.041
77.000
76.679
46.483
25.397
24.253
23.842
23.282
PPM
150
125
100
75
50
25

2-(4-Chlorophenyl)-3,3,4-trimethylpent-4-en-2-ol (3cb)

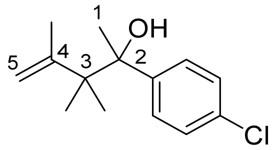

IR (neat) 3510 cm^{-1} (OH)   bp: 230 °C /1.3 torr

HRMS calcd for $C_{14}H_{18}ClO$: 237.10517, found: m/z 237.10486 (ESI, M^{-}-1,)

^{1}H NMR ($CDCl_3$, 400 MHz) : δ7.37-7.34 (m, 2H, Ph), 7.27-7.24 (m, 2H, Ph), 5.06 (s, 1H, <u>*H*</u>HC=C), 4.90 (s, 1H, H<u>*H*</u>C=C), 2.40 (s, 1H, OH), 1.61 (s, 3H, H_2C=CC<u>*H*</u>$_3$ or C<u>*H*</u>$_3$COH), 1.57 (s, 3H, H_2C=CC<u>*H*</u>$_3$ or C<u>*H*</u>$_3$COH), 1.11 (s, 3H, C<u>*H*</u>$_3$CCH$_3$), 1.04 (s, 3H, CH$_3$CC<u>*H*</u>$_3$)

^{13}C NMR ($CDCl_3$,100 Hz): δ151.1, 144.1, 132.3, 128.5, 127.1, 114.4, 77.0, 46.5, 25.6, 24.2, 23.9, 23.6

^{1}H NMR

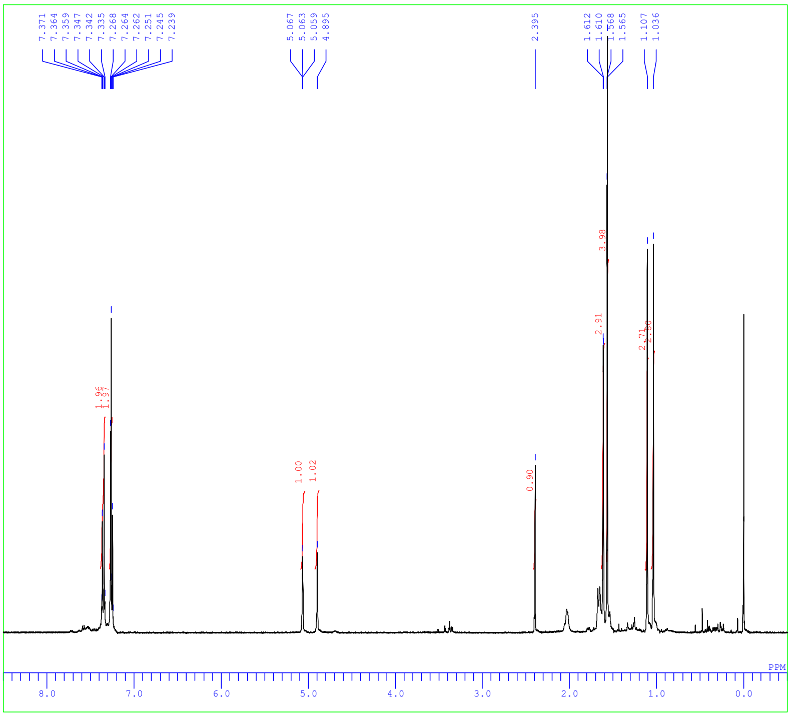

^{13}C NMR

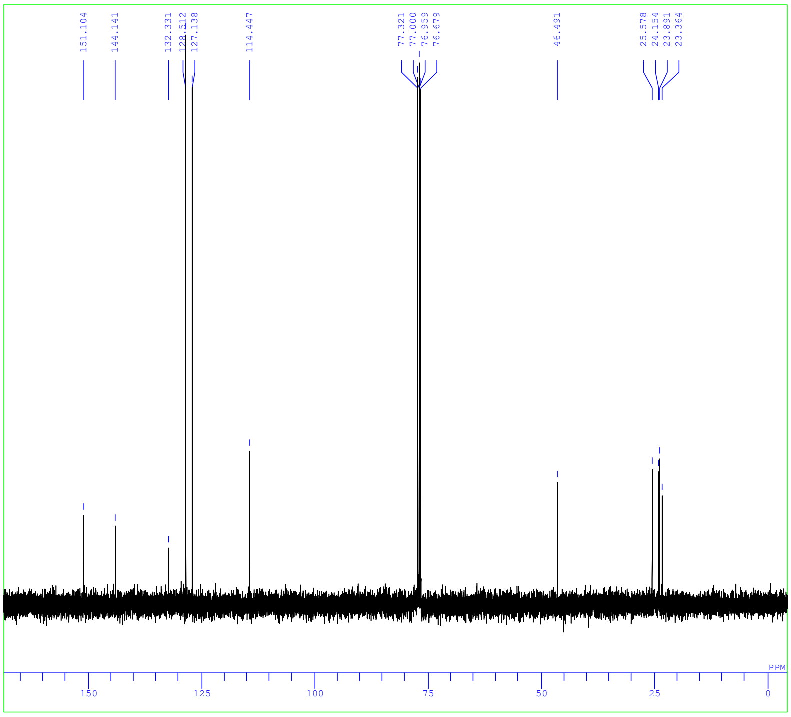
151.104
144.141
132.331
128.512
127.138
114.447
77.321
77.000
76.959
76.679
46.491
25.578
24.154
23.891
23.364
PPM
150
125
100
75
50
25
0

2-(4-Bromophenyl)-3,3,4-trimethylpent-4-en-2-ol (3cc)

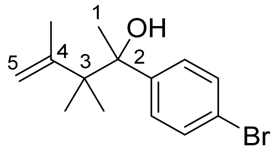

IR (neat) 3527 cm^{-1} (OH) bp: 250 °C /0.93 torr

Anal. calcd for $C_{14}H_{19}BrO$: C, 59.37; H, 6.76, found: C, 59.60; H, 6.97

^{1}H NMR ($CDCl_3$, 400 MHz): δ7.41–7.39 (m, 2H, Ph), 7.30–7.26 (m, 2H, Ph), 5.06 (s, 1H, *H*HC=C), 4.89 (s, 1H, H*H*C=C), 2.39 (s, 1H, OH), 1.61 (s, 3H, H_2C=CC*H*$_3$ or C*H*$_3$COH), 1.56 (s, 3H, H_2C=CC*H*$_3$ or C*H*$_3$COH), 1.10 (s, 3H, C*H*$_3$CCH$_3$), 1.03 (s, 3H, CH$_3$CC*H*$_3$)

^{13}C NMR ($CDCl_3$,100 Hz): δ150.9, 144.6, 130.0, 128.9, 120.5, 114.4, 77.0, 46.3, 25.4, 24.1, 23.8, 23.3

^{1}H NMR

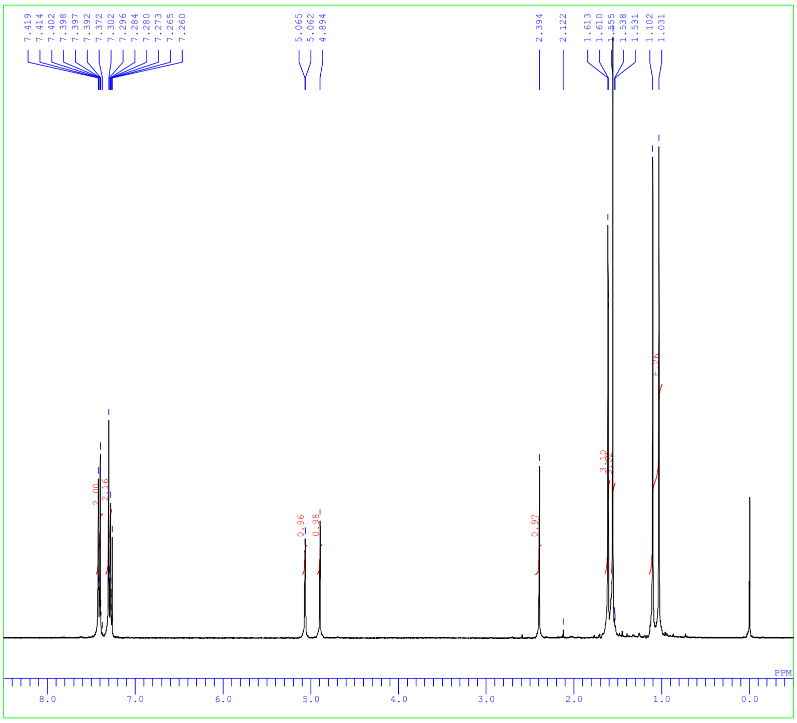

^{13}C NMR

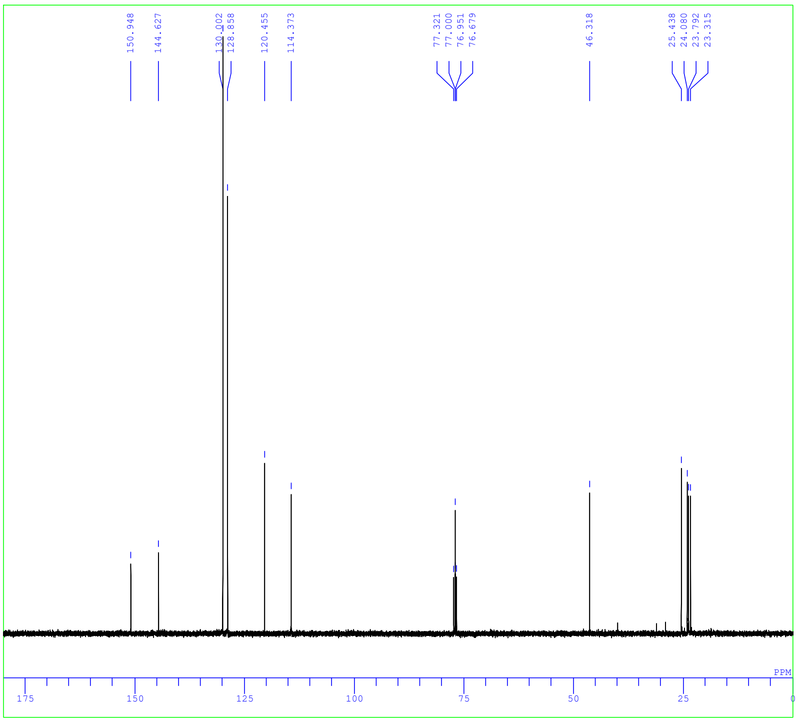
150.948
144.627
130.002
128.858
120.455
114.373
77.321
77.000
76.951
76.679
46.318
25.438
24.080
23.792
23.315
PPM
175
150
125
100
75
50
25
0

3-Hydroxy-4,4,5-trimethyl-3-phenylhex-5-enenitrile (3ci)

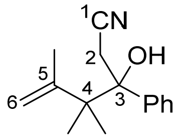

white solid

IR (neat) 3410 cm^{-1} (OH), 2263 cm^{-1} (CN) ; m.p. 117.0 °C

HRMS calcd for $C_{15}H_{20}NO$: 230.1545, found: m/z 230.1543 (CI, M^{+}+1, -0.2 mmu)

Anal. calcd for $C_{15}H_{19}NO$: C, 78.56; H, 8.35; N, 6.11, found: C, 78.39; H, 8.32; N, 5.96

^{1}H NMR ($CDCl_3$, 400 MHz) : δ7.40–7.30 (m, 5H, Ph), 5.10 (s, 1H, *H*HC=C), 4.91 (s, 1H, H*H*C=C), 3.26 (d, *J* = 16.7 Hz, 1H, C*H*HCN), 3.00 (d, *J* = 16.7 Hz, 1H, CH*H*CN), 2.69 (s, 1H, OH), 1.65 (s, 3H, H_2C=CCH_3), 1.09 (s, 3H, CH_3CCH_3), 1.07 (s, 3H, CH_3CCH_3)

^{13}C NMR ($CDCl_3$,100 Hz): δ150.0, 140.9, 127.8, 127.7, 127.0, 118.2, 115.1, 78.7, 46.2, 27.9, 24.2, 23.3, 22.9

^{1}H NMR

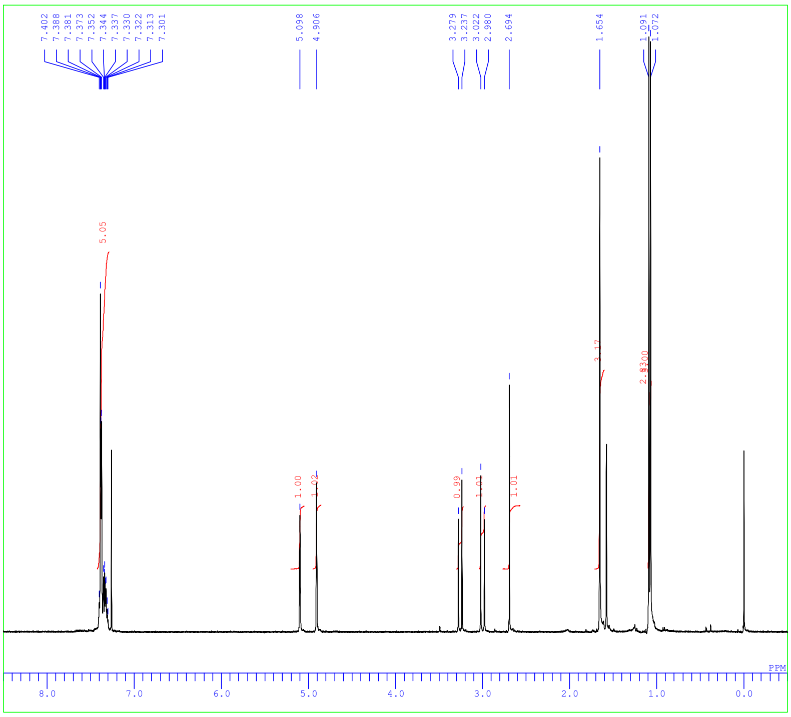

^{13}C NMR

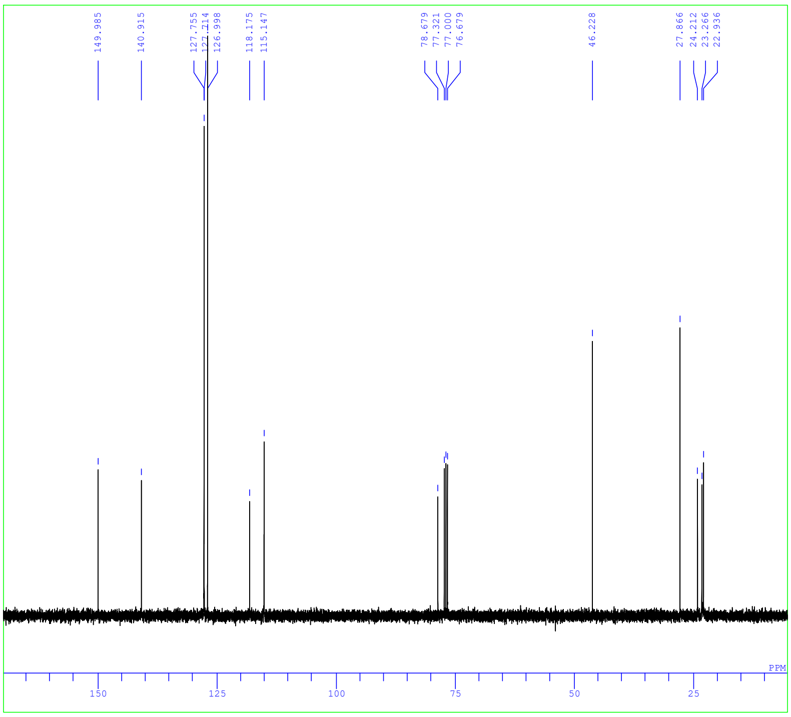
149.985
140.915
127.755
127.214
126.998
118.175
115.147
78.679
77.321
77.000
76.679
46.228
27.866
24.212
23.266
22.936
PPM
150
125
100
75
50
25

1-Bromo-3,3,4-trimethyl-2-phenylpent-4-en-2-ol (3aj)

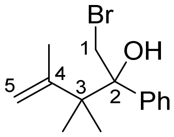

IR (neat) 3548 cm^{-1} (OH) b.p.: 140 °C/ 0.16 torr

HRMS calcd for $C_{14}H_{19}ONaBr$: 305.05115, found: m/z 305.05054 (ESI, M^{+}+Na)

^{1}H NMR ($CDCl_3$, 400 MHz) : δ7.38–7.22 (m, 5H, Ph), 5.01 (s, 1H, _H_HC=C), 4.82 (s, 1H, H_H_C=C), 4.27 (d, *J* =10.6 Hz, 1H, C_H_HBr), 4.01 (d, *J* =10.6 Hz, 1H, CH_H_Br), 2.57 (s, 1H, OH), 1.76 (s, 3H, H_2C=CC$\underline{H}_3$), 1.13 (s, 3H, C$\underline{H}_3$CCH_3), 1.08 (s, 3H, CH_3CC$\underline{H}_3$)

^{13}C NMR ($CDCl_3$,100 Hz): δ150.4, 141.7, 127.7, 127.2, 127.1, 114.4, 79.5, 46.0, 43.4, 24.7, 24.0, 23.0

^{1}H NMR

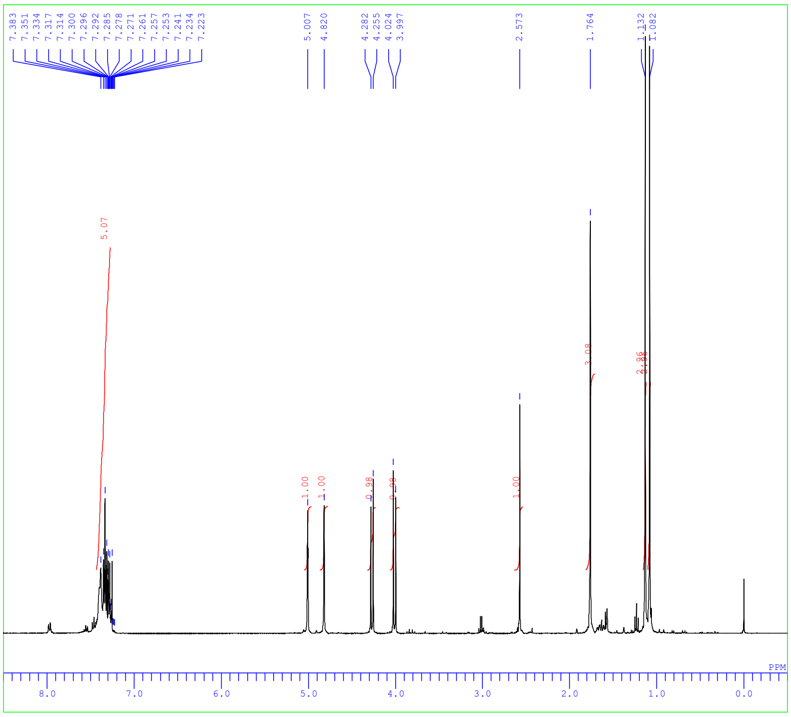

^{13}C NMR

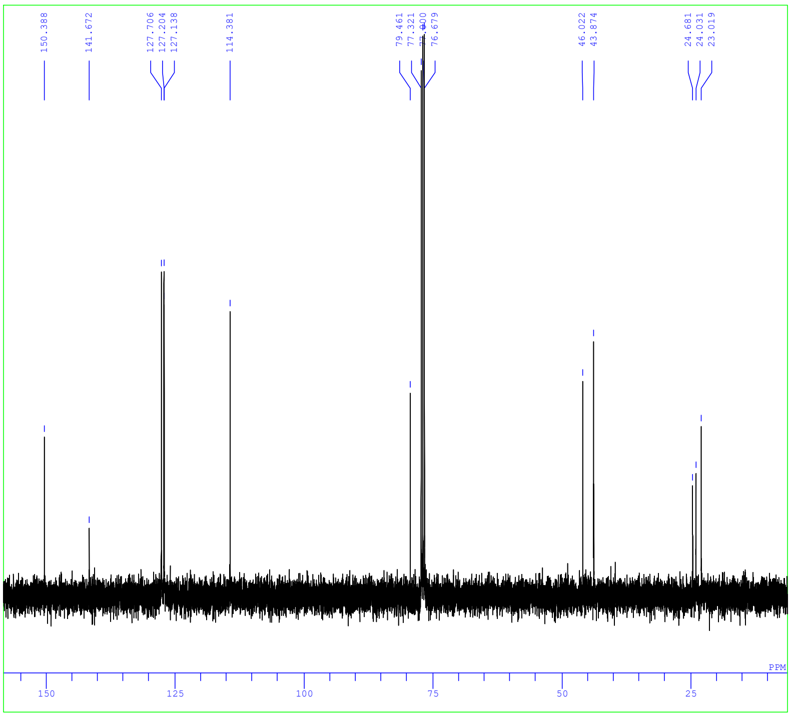

150.388
141.672
127.706
127.204
127.138
114.381
79.461
77.321
76.679
46.022
43.874
24.681
24.031
23.019
PPM
150
125
100
75
50
25

Diethyl 2-(2-(1-hydroxy-1-phenylethyl)but-3-en-1-yl)malonate (5a)

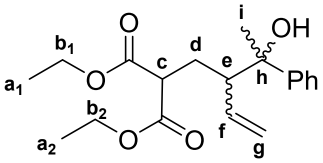

Major isomer

IR (neat) 3527 cm^{-1} (OH), 1724 cm^{-1} (C=O), b.p.: 230 °C/ 0.38 torr

^{1}H-NMR ($CDCl_3$, 400 MHz) δ: 7.40-7.21 (m, 5H, Ph), 5.71-5.62 (m, 1H, $H_2C=C\underline{H}$), 5.25 (dd, *J* = 10.3, 1.8 Hz, 1H, $\underline{H}$HC=CH), 5.11 (dd, *J* = 17.4, 1.7 Hz, 1H, H$\underline{H}$C=CH), 4.17-4.01 (m, 4H, $OC\underline{H}_2$ x 2), 3.28 (dd, *J* = 11.1, 3.9 Hz, 1H, $C\underline{H}(CO_2Et)_2$), 2.40-2.34 (m,1H, $H_2C=CHC\underline{H}$), 1.98-1.91 (m, 1H, $C\underline{H}HCH(CO_2Et)_2$), 1.78-1.71 (m, 1H, $CH\underline{H}CH(CO_2Et)_2$), 1.51 (s, 3H, $C\underline{H}_3COH$), 1.22-1.18 (m, 6H, OCH_2CH_3 x 2).

^{13}C NMR ($CDCl_3$,100 Hz):

δ169.6, 169.2, 146.5, 136.8, 128.0, 126.6, 125.0, 119.6, 75.7, 61.3, 61.1, 53.1, 50.1, 29.2, 27.9, 14.0

MS (CI, 200 eV) m/z 157 (1188), 317 (821), 318 (161), 158 (146), 121 (44), 319 (24)

HRMS calcd for $[C_{19}H_{26}O_5\text{-}OH]^+$: 317.1747, found: m/z 317.1749 (CI)

Minor isomer

IR (neat) 3524 cm^{-1} (OH), 1722 cm^{-1} (C=O), b.p.: 228 °C/ 0.46 torr

^{1}H-NMR ($CDCl_3$, 400 MHz) 1H-NMR ($CDCl_3$) δ: 7.42-7.22 (m, 5H, Ph), 5.57-5.48 (m, 1H, $H_2C=C\underline{H}$), 5.26 (dd, *J* = 10.1, 1.8 Hz, 1H, $\underline{H}$HC=CH), 5.15 (dd, *J* = 17.0, 1.8 Hz, 1H, H$\underline{H}$C=CH), 4.16-4.01 (m, 4H, $OC\underline{H}_2$ x 2), 3.27 (dd, *J* = 10.9, 4.1 Hz, 1H, $C\underline{H}(CO_2Et)_2$), 2.36-2.30 (m, 1H, $H_2C=CHC\underline{H}$), 2.23-2.16 (m, 1H, $C\underline{H}HCH(CO_2Et)_2$), 1.65-1.57 (m, 5H, $CH\underline{H}CH(CO_2Et)_2$ & $C\underline{H}_3COH$), 1.24-1.18 (m, 6H, $OCH_2C\underline{H}_3$ x 2).

^{13}C NMR ($CDCl_3$, 100 Hz):

δ169.6, 169.2, 145.6, 137.0, 127.9, 126.8, 125.7, 119.8, 75.3, 61.3, 61.2, 54.2, 50.1, 27.9, 26.6, 14.0, 13.9

MS (CI, 200 eV) m/z 317 (1151), 157 (591), 318 (238), 158 (80), 319 (49), 214 (48), 289 (27), 121 (25)

HRMS calcd for $[C_{19}H_{26}O_5\text{-}OH]^+$: 317.1747, found: m/z 317.1751 (CI)

^{1}H NMR (major isomer)

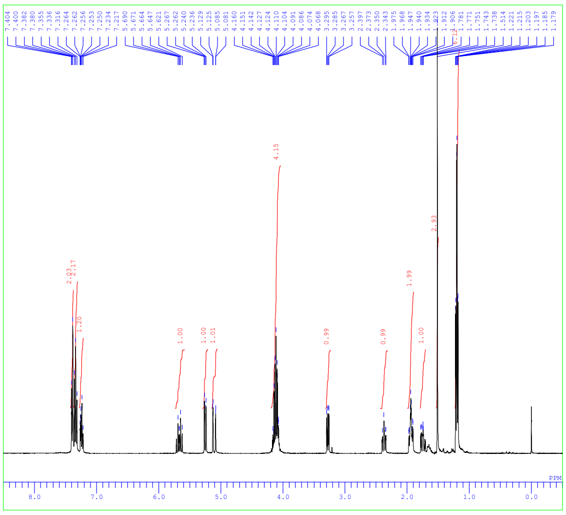

^{13}C NMR (major isomer)

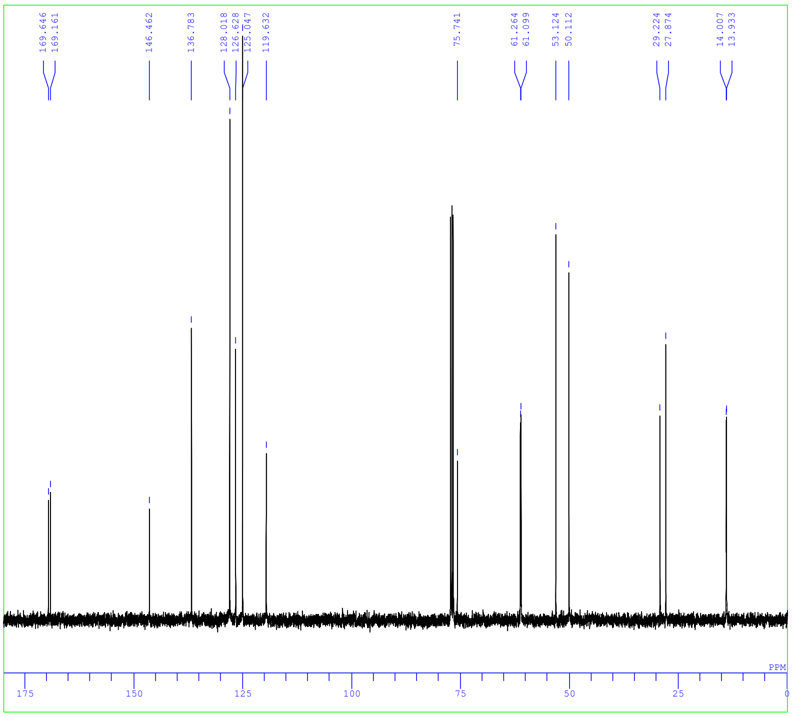

^{1}H NMR (minor isomer)

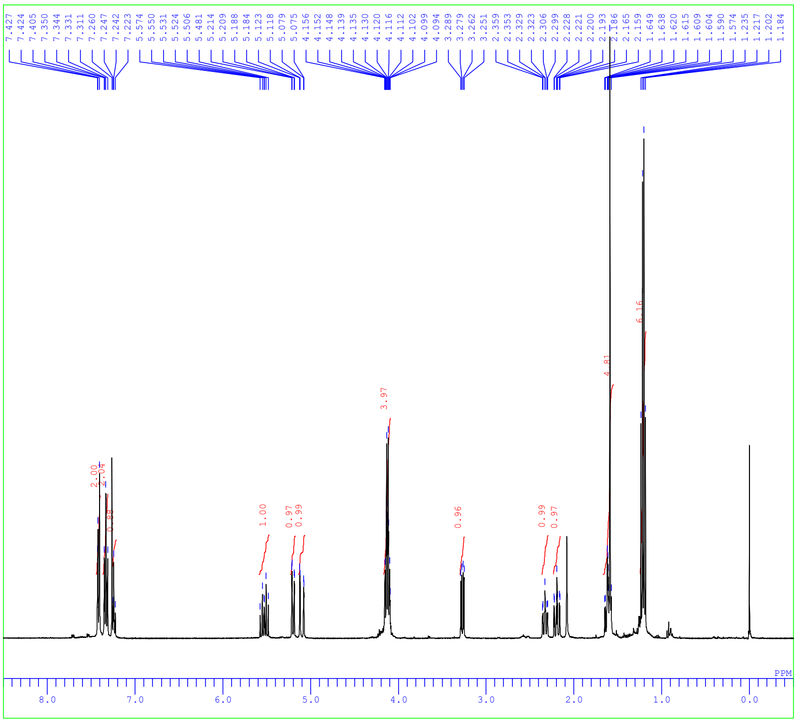

^{13}C NMR (minor isomer)

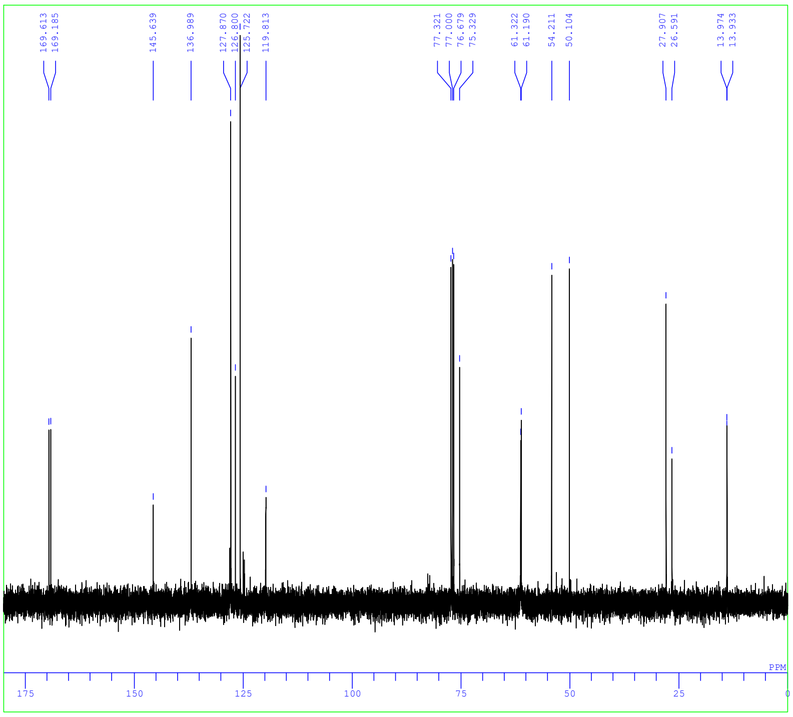

1 R. Ieki, Y. Kani, S. Tsunoi and I. Shibata, *Chem. Eur. J.*, 2015, **21**, 6295-6300.

2 C. Li, R. Y. Liu, L. T. Jesikiewicz, Y. Yang, P. Liu and S. L. Buchwald, *J. Am. Chem. Soc.*, 2019, **141**, 5062-5070.

3 P. Dey, M. Koli, D. Goswami, A. Sharma and S. Chattopadhyay, *Eur. J. Org. Chem.*, 2018, 1333-1341.

4 L. Jiaming, Z. Zhenggen, S. Lilin, Z. Yan and W. Zhiyong, *Chem. Lett.*, 2006, **35**, 498-499.

5 F. Nowrouzi, A. N. Thadani and R. A. Batey, *Org. Lett.*, 2009, **11**, 2631-2634.

6 Y. Yatsumonji, T. Nishimura, A. Tsubouchi, K. Noguchi and T. Takeda, *Chem. Eur. J.*, 2009, **15**, 2680-2686.

7 R. Wada, K. Oisaki, M. Kanai and M. Shibasaki, *J. Am. Chem. Soc.*, 2004, **126**, 8910-8911.

8 N. Hayashi, H. Honda, M. Yasuda, I. Shibata and A. Baba, *Org. Lett.*, 2006, **8**, 4553-4556.
